# Rules of engagement for condensins and cohesins guide mitotic chromosome formation

**DOI:** 10.1126/science.adq1709

**Published:** 2025-04-11

**Authors:** Kumiko Samejima, Johan H. Gibcus, Sameer Abraham, Fernanda Cisneros-Soberanis, Itaru Samejima, Alison J. Beckett, Nina Pučeková, Maria Alba Abad, Christos Spanos, Bethan Medina-Pritchard, James R. Paulson, Linfeng Xie, A. Arockia Jeyaprakash, Ian A. Prior, Leonid A. Mirny, Job Dekker, Anton Goloborodko, William C. Earnshaw

**Affiliations:** 1 Wellcome Centre for Cell Biology, Institute of Cell Biology, University of Edinburgh; Edinburgh, UK; 2 Department of Systems Biology, University of Massachusetts Chan Medical School; Worcester, USA; 3 Institute for Medical Engineering and Science and Department of Physics, Massachusetts Institute of Technology; Cambridge, USA; 4 Department of Chemistry, University of Wisconsin-Oshkosh; Oshkosh, USA.; 5 Gene Center Munich, Ludwig-Maximilians-Universität München; Munich, Germany; 6 Department of Molecular and Clinical Cancer Medicine, University of Liverpool; Liverpool, UK.; 7 Howard Hughes Medical Institute; Chevy Chase, USA; 8 Institute of Molecular Biotechnology; Vienna, Austria

## Abstract

We used Hi-C, imaging, proteomics and polymer modeling to define rules of engagement for SMC complexes as cells refold interphase chromatin into rod-shaped mitotic chromosomes. Firstly, condensin disassembles interphase chromatin loop organization by evicting or displacing extrusive cohesin. Secondly, condensin bypasses cohesive cohesins, thereby maintaining sister chromatid cohesion as sisters separate. Studies of mitotic chromosomes formed by cohesin, condensin II and condensin I alone or in combination reveal new models of mitotic chromosome conformation. In these models, loops are consecutive and not overlapping, implying that condensins stall upon encountering each other. The dynamics of Hi-C interactions and chromosome morphology reveal that during prophase loops are extruded in vivo at ~1–3 kb/sec by condensins as they form a disordered discontinuous helical scaffold within individual chromatids.

## Abstract (summary)

**Introduction:**

As cells enter mitosis, they transform their chromosomes from the decondensed interphase state into individual rod-shaped and compacted sister chromatid pairs that remain connected along their length until they segregate at anaphase. This transformation is largely driven by structural maintenance of chromosomes (SMC) complexes acting as molecular motors that actively extrude chromatin loops, alongside a second function of mediating sister chromatid cohesion. In mitosis, each sister chromatid folds as a bottlebrush-like nested array of consecutive loops, that tend to be helically arranged. This compact arrangement is generated through collective activity of four different SMC complexes: condensins I and II, loop-extruding cohesin, and cohesive cohesin linking sister chromatids.

**Rationale:**

The various SMC complexes acting on mitotic chromosomes must coordinate their activities to drive compaction and folding of each individual sister chromatid and erase interphase organization, while maintaining cohesion between sisters along their length. The rules governing this coordination between complexes are unknown. In this study we applied a combination of cell biology, imaging, proteomics, Hi-C, and polymer modeling to cells in which various combinations of SMC complexes were acutely depleted prior to synchronous entry into mitosis. Time-resolved analysis of the subsequent mitotic chromosome formation then allowed us to identify specific roles of each complex and reveal their interplay.

**Results:**

By measuring growth of loop size in cells expressing only a single condensin complex, we determined that condensin I and II extrude chromatin in loops with a speed of 1–3 kb per second in living eukaryotic cells. Through polymer modeling and testing results of simulations against experimental imaging and Hi-C data, we could then define “rules of engagement” that dictate what occurs when these fast-moving complexes encounter one another along the chromatin fiber. First, when a condensin runs into an extruding cohesin complex, cohesin is removed from the chromatin, and condensin continues its loop extrusion, thus “erasing” interphase organization. In the absence of condensin, this organization persists well into mitosis even though the chromosomes condense into amorphous structures. Second, when a condensin runs in a cohesive cohesin complex, it steps over it and keeps extruding. As a result, sister chromatids can separate their axes, while remaining cohesed between the tips of their loops. Third, when a condensin approaches another condensin it stalls. This produces arrays of consecutive loops.

Our data also allowed refinement of previous models of individual mitotic chromatids revealing, for example, that loops are separated from each other by small gaps, thereby lengthening the scaffold. The loop array follows a disorderly helical path inside each chromatid. This periodic structure is seen in Hi-C maps as multiple diagonals spaced apart by the average length of one helical turn. Condensins I and II act independently and their combined action results in a nested loop organization. Cohesion between loops of sister chromatids is consistent with such an organization, but constrains the helical winding, preventing each sister chromatid from forming a more regular helix. Due to the dynamic interactions and stochastic nature of binding and loop extrusion processes, mitotic chromosomes do not adopt a single, fixed 3D structure. Instead, they are disorderly structures with a common defined architecture.

**Conclusion:**

Our study identifies three rules of engagement for condensins and cohesin complexes. Applying these rules, we can begin to understand how SMC complexes acting on chromatin at the nanometer scale together convert organized interphase chromosomes into cohesed pairs of compacted and separated sister chromatids at the micron scale. Other rules of engagement may apply in different conditions (i.e., in interphase), in meiosis, and in different organisms to drive a range of chromosome conformations formed via the same underlying loop extrusion process.

## Graphical Abstract

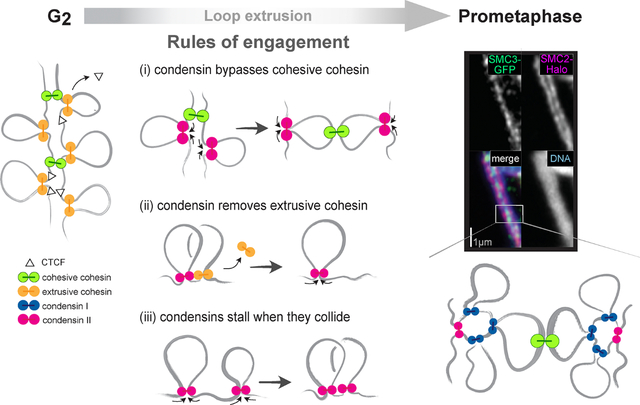

Print summary figure caption

Rules of engagement define interactions between SMC complexes during mitotic chromosome formation.

In prophase, loop-extruding condensins transform cohesin-shaped interphase chromosomes into mitotic chromosomes. This transformation involves interactions between cohesin and condensin dictated by three rules of engagement that we describe here. These rules explain how interphase organization is erased and chromosomes become highly compacted in bottle-brushes while staying cohesed along their length.

The structural maintenance of chromosomes (SMC) protein complexes cohesin and condensin are key determinants of chromatin organization in interphase and in mitosis (1–4). During prophase, a cohesin-dominated interphase organization with topologically associating domains (TADs) and compartments transitions into a condensin-dominated mitotic loop array (5, 6). Therefore, encounters between cohesins and condensins are likely to be frequent during this time. When bacterial SMC complexes collide, Hi-C and modeling studies (7) suggest they can bypass each other (8), consistent with single-molecule studies of yeast condensin (9). In contrast, the “rules of engagement” when condensins encounter cohesins and each other during mitotic chromosome formation in metazoa remain underexplored.

SMC complexes generate loops via ATP-dependent loop extrusion (other mechanisms may also contribute) (1, 10–14). Throughout interphase, extrusive cohesin complexes organize the genome into loops that help regulate gene expression (15). Additionally, cohesive cohesin complexes establish cohesion during S-phase, and maintain the pairing of sister chromatid arms until anaphase (16–23). Cohesin has also been suggested to antagonize chromatid axis formation in Xenopus extracts (24).

Most cohesin is released from chromosome arms during prophase (25, 26). Also, at this time, condensin II in the nucleus activated by CDK1 and Plk1 (27–29), associates stably with the chromatin, and begins to extrude a sequence-non-specific loop array. Thus, a dynamic exchange of SMC complexes occurs throughout prophase. Upon nuclear envelope breakdown, cytoplasmic condensin I associates with chromosomes, forming loops nested within the larger condensin II loops (6, 30). The resulting densely packed “bottlebrush-like” arrays of consecutive loops (corresponding to the chromonema (31)) produce the classical rod-shape of mitotic chromosomes (32) with paired sister chromatids aligned along their length (33–35).

Here we construct a series of single- and multiple-mutant degron cell lines to examine the action of specific combinations of cohesin, condensin I or condensin II complexes in chromosome formation during highly synchronous mitotic entry. Hi-C and microscopy data have (1) revealed that cohesive, but not extrusive, cohesin substantially influences the structure of mitotic chromosomes; (2) determined how SMC complexes engage with each other during chromosome formation; (3) allowed us to create models for chromosome organization formed by SMC complexes acting singly and in combination, and (4) allowed us to determine the speed of loop extrusion by condensins in vivo.

## RESULTS:

### Substantial levels of cohesin remain on prometaphase chromosomes

Live cell imaging of DT40 CDK1^as^ cells that undergo a highly synchronous mitotic entry (6, 36) (Fig. S1A,B) revealed that early prophase chromatin condensation starts about 3 minutes after release from G_2_ block (t= 0 min). Condensing chromatin is concentrated at the inner surface of the nuclear envelope and around nucleoli, where rod-shaped chromosomes became distinct at t= ~9 min (late prophase). At t= 10–12 min, the nuclear rim of lamin B1 becomes discontinuous (NEB), and individual chromosomes are released (prometaphase).

**Fig. 1:**
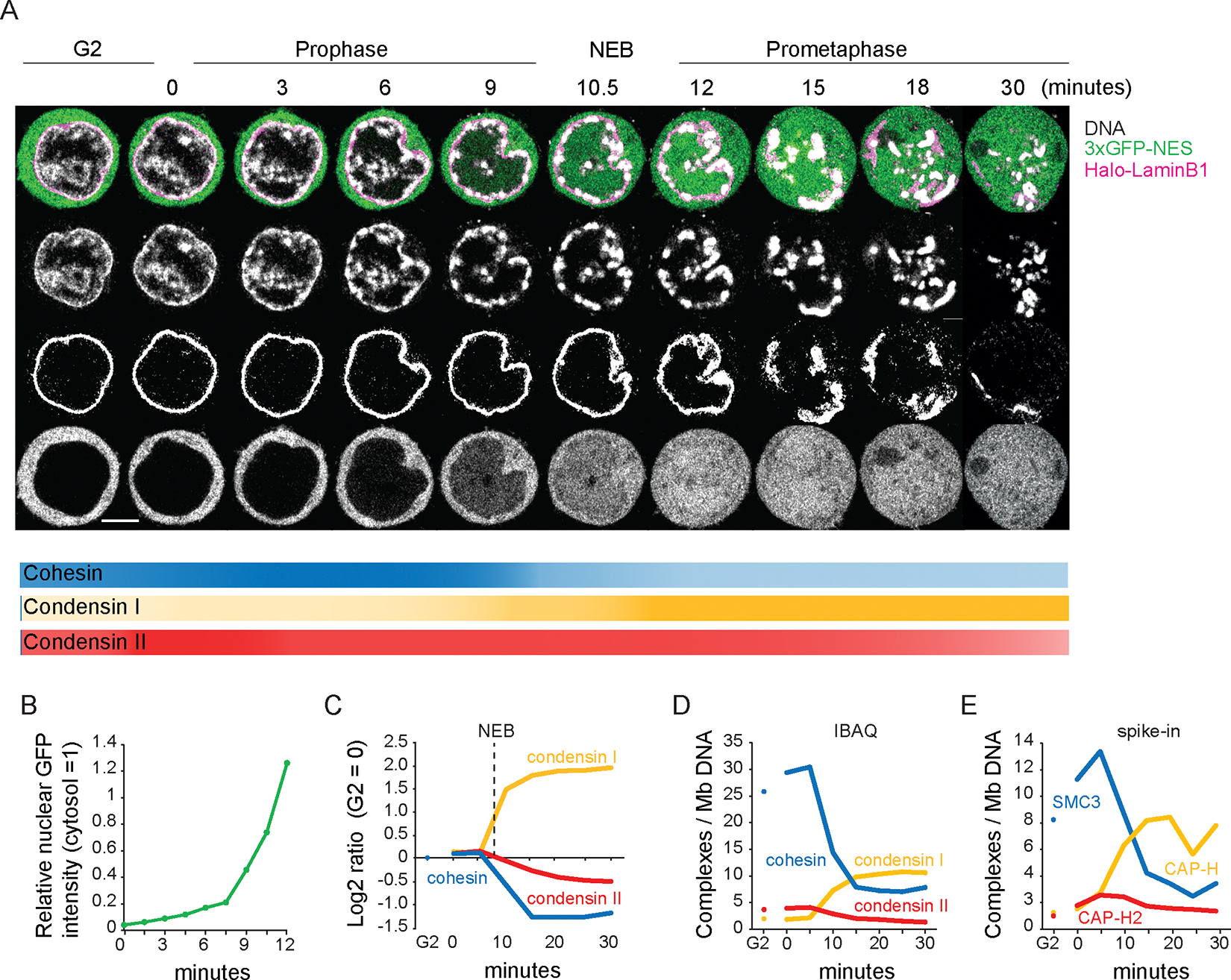
Dynamics of chromatin bound cohesin and condensin during mitotic entry. (**A**) Representative live cell imaging of a DT40 CDK1^as^_Halo-lamin B1_3xGFP-NES cell released from G_2_ block with 1NM-PP1. DNA:grey; Halo-lamin B1: magenta; 3xGFP-NES: green. Seven z-sections (1 μm interval) were taken every 1.5 min. A single section is shown for each timepoint. Scale bar = 5 μm. 3XGFP enters nuclei a few minutes prior to visible nuclear lamina disruption (NEB, t= 10–11 min). Intensity of lines under the images illustrates the relative amount of the indicated complexes on chromatin at each time point. Green arrow indicates cytoplasmic GFP entering the nucleus before nuclear envelope breakdown. (**B**) Relative nuclear GFP fluorescence intensity (cytosolic GFP intensity = 1) from experiment of Fig. 1A. (**C**) Chromatin enrichment for proteomics (ChEP) analysis of WT CDK1^as^ cells (SILAC analysis). Log_2_ SILAC ratio normalized against G_2_ is shown for cohesin (average of SMC1, SMC3, and RAD21), condensin I (CAP-H, CAP-G, and CAP-D2) and condensin II complexes (CAP-H2, CAP-G2, and CAP-D3). t= 0 is after completion of 1NM-PP1 washout, n= 6. (**D**) Estimated number of chromatin-associated cohesin, condensin I and condensin II complexes (per Mb DNA) during mitotic entry in wild type CDK1^as^ cells. Average iBAQ number from ChEP analysis for subunits as listed in C was normalized relative to values for Histone H4, n= 6. (**E**) Absolute quantification of SMC3 (cohesin subunit), CAP-H (condensin I subunit) and CAP-H2 (condensin II subunit) on chromatin (per Mb DNA). Protein numbers are calculated following ChEP analysis of corresponding Halo-tagged cell lines normalized using purified spike-in Halo-Histone H4 protein (n= 4).

Chromatin-bound cohesin detected using ChEP (chromatin enrichment for proteomics) was highly abundant in G_2_ (Fig. 1C–E, Fig. S2A). Bound cohesin levels increased slightly in early prophase, then decreased approximately 3-fold during late prophase and after NEB. Condensin II was nuclear during G_2_, with chromatin-associated condensin II increasing slightly in early prophase, then gradually decreasing by about 50% from prophase to late prometaphase (Fig. 1C–E, Fig. S2B). Condensin I, which is predominantly cytosolic during interphase (37, 38), accumulated on chromatin gradually during late prophase and then rapidly after NEB, ultimately reaching a level about 5x that of condensin II. Chromatin accumulation of condensin I paralleled behavior of 3xGFP-NES (three GFP domains fused to a nuclear export domain), which is too large to diffuse through nuclear pores. GFP intensity increased gradually in the nucleus several minutes prior to NEB and then increased dramatically during and after NEB (Fig. 1A: 9 min, and 1B) (39). Quantitative spike-in-normalized proteomic analysis revealed that late prometaphase chromosomes contained substantial amounts of residual cohesin and newly bound condensin I (~3–6 and ~10 complexes per megabase, respectively - Fig. 1D,E). Proteomic analysis cannot distinguish arm from centromeric cohesin, nor distinguish extrusive from cohesive cohesin.

These data show that condensin I accesses chromatin as early as mid-late prophase, and that cohesin remains abundant on chromatin till late prometaphase (Fig. 1). Co-occurrence of the various SMC complexes suggests that their interactions, including potential clashes, influence mitotic chromosome formation and the removal of interphase chromatin structures. Thus, cohesin could have a previously unsuspected role in mitotic chromosome formation.

### Condensin drives the disassembly of interphase chromatin structures

As noted previously (6) and analyzed quantitatively here using Hi-C, loss of interphase features is condensin dependent. Features lost include TADs, dots and stripes formed by loop-extruding cohesin and global compartments. Here, we quantitiated this loss during prophase using new CDK1^as^ cell lines expressing OsTIR1 plus a series of single and multiple homozygous auxin-inducible degron alleles of cohesin, condensin I and II (Table S1). This enabled us to mechanistically examine the role of condensin during prophase and test our hypotheses using polymer simulations.

**Fig. 2.**
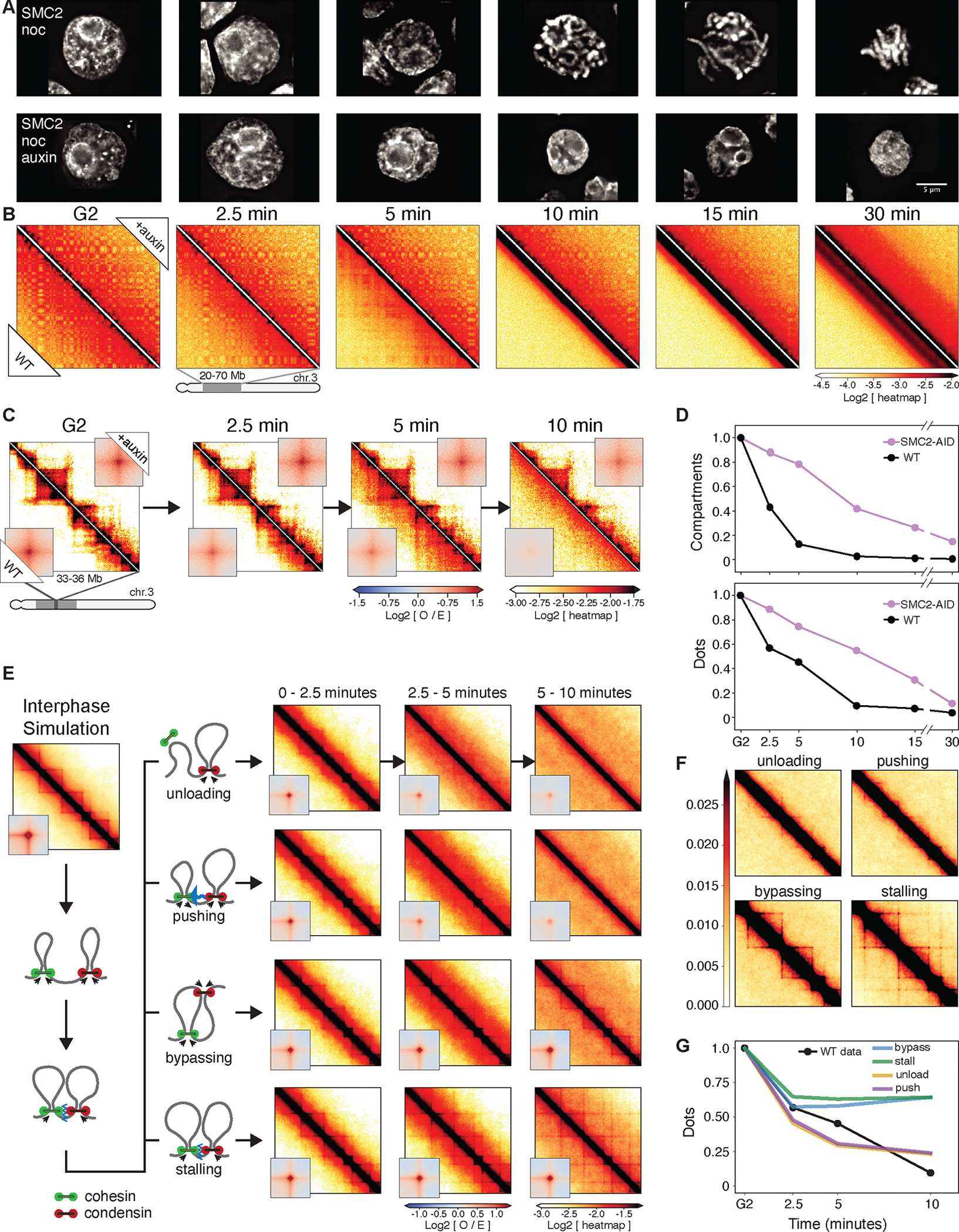
Condensin facilitates disassembly of interphase chromatin organization during mitotic entry. (**A**) Representative images of DAPI-stained SMC2-AID cells used to prepare Hi-C samples. Nocodazole was added 30 minutes prior to 1NM-PP1 washout. Top row: cells from control culture (no auxin treatment). Bottom row: cells from culture treated with auxin during the G_2_ block and release into mitosis. Scale bar = 5 μm. (**B**) Hi-C contact maps of wild-type cells (WT) and SMC2-AID cells treated with auxin (+auxin) from cell populations shown in panel A. A region from chromosome 3 (position 20–70 Mb) is shown. Bottom triangle of each Hi-C map displays Hi-C data obtained with control cells; top triangle displays Hi-C data obtained with auxin treated cells. The checkerboard pattern, readily observed in G_2_ cells, reflects compartmentalization. Compartments disappeared quickly in WT cells but remained in SMC2-depleted mitotic cells. (**C**) Same as in panel B, but zoomed in for chromosome 3 (region 33–36 Mb). Insets show the distance-normalized contact enrichment at detected dots genome-wide. TADs and dots persist in SMC2-depleted mitotic cells. (**D**) Quantification of features shown in panel B (compartments) and panel C (dots). Compartment and dot strength were normalized to their values in G_2_-arrested cells, which was set at 1. (**E**) Outline of four possible simulated scenarios of collisions between cohesin and condensins in prophase (left) and the corresponding simulated Hi-C maps (right, on log scale). (**F**) Same simulated Hi-C maps as in panel E, but in linear scale to better emphasize cohesin-dependent features including lines and dots. (**G**) Quantification of dots in panel E as predicted by the 4 simulated scenarios shown in panel E and in comparison to WT experimental Hi-C data. Dot strength was normalized to G_2_, which was set at 1.

Mitotic cells depleted of SMC2 (i.e. lacking both condensin I and II) entered mitosis with normal kinetics (Fig. S1D), but rod-shaped chromosomes did not form even at late prometaphase (Fig. 2A), as previously reported (6, 14, 40–42). Nonetheless, the chromatin underwent a ~3-fold reduction in volume by prometaphase (t= 30 min) (Fig. 2A, Table S2). This is comparable to the volume decrease observed in normal mitotic cells (41, 43–45). This confirms previous conclusions that mitotic chromosome formation involves condensin-dependent rod formation and condensin-independent volume compaction (6, 41, 46).

Quantitative analysis of chromosome organization from Hi-C data at high temporal resolution (2.5’, 5’, 7.5’, 10’, 15’, 30’ after synchronous release from G_2_) reveals that condensins actively promote disassembly of cohesin-dependent interphase features. In WT cells, the strength of compartments (Fig. 2B,D), dots (i.e., CTCF-CTCF loops), and TADs (Fig. 2C,D) decayed rapidly in the first 10 minutes after release from the CDK1^as^ block, becoming undetectable by the onset of NEB. In contrast, in condensin-depleted cells, these features persisted until late prometaphase (Fig. 2C,D). Thus, condensin can disrupt compartmental associations, possibly analogous to the weakening of compartmentalization by cohesin loop extrusion (47–49). Additionally, condensin disrupts cohesin-mediated loops. The prolonged presence of dots in Hi-C maps, i.e., positioned loops at CTCF-bound sites into prometaphase in condensin-depleted cells reveals that both cohesin and CTCF remain bound. ChEP data confirm that CTCF and cohesin loss from chromosomes is delayed in the absence of condensin (Fig. S2A,C).

We used polymer simulations to explore four possible interactions between condensins and loop-extruding cohesins: when the two complexes meet each other, cohesin is (1) unloaded, (2) pushed along the chromosome by condensin, (3) bypassed by condensin, or (4) both complexes stall until cohesin unbinds. We simulated these 4 scenarios and quantified the loss of cohesin-mediated features. Our analysis clearly revealed that only unloading and pushing can displace dot-forming CTCF-stalled cohesins and reproduce the fast loss of interphase features (Fig. 2E,F). A slower condensin-independent loss of TADs and dots, observed in SMC2-depleted cells, is explained by a “background” pathway, possibly involving gradual loss of CTCF and cohesin as seen in the proteomics (ChEP) data (Fig S2A,C). In wild type cells, the much faster condensin-driven loss obscures the slower-acting “background” pathway.

Thus, the rapid disassembly of interphase chromosome folding during mitotic entry can be explained by disruption of cohesin loops by condensins. In addition to stalling and bypassing observed in prior studies (9), our data and simulations suggest that interactions between the complexes can lead to selective unloading and/or pushing of extrusive cohesin. Selective depletion of either condensin I or II shows that both condensins contribute to this disassembly of interphase features.

### Cohesin localizes between sister chromatids away from condensin

**Fig. 3.**
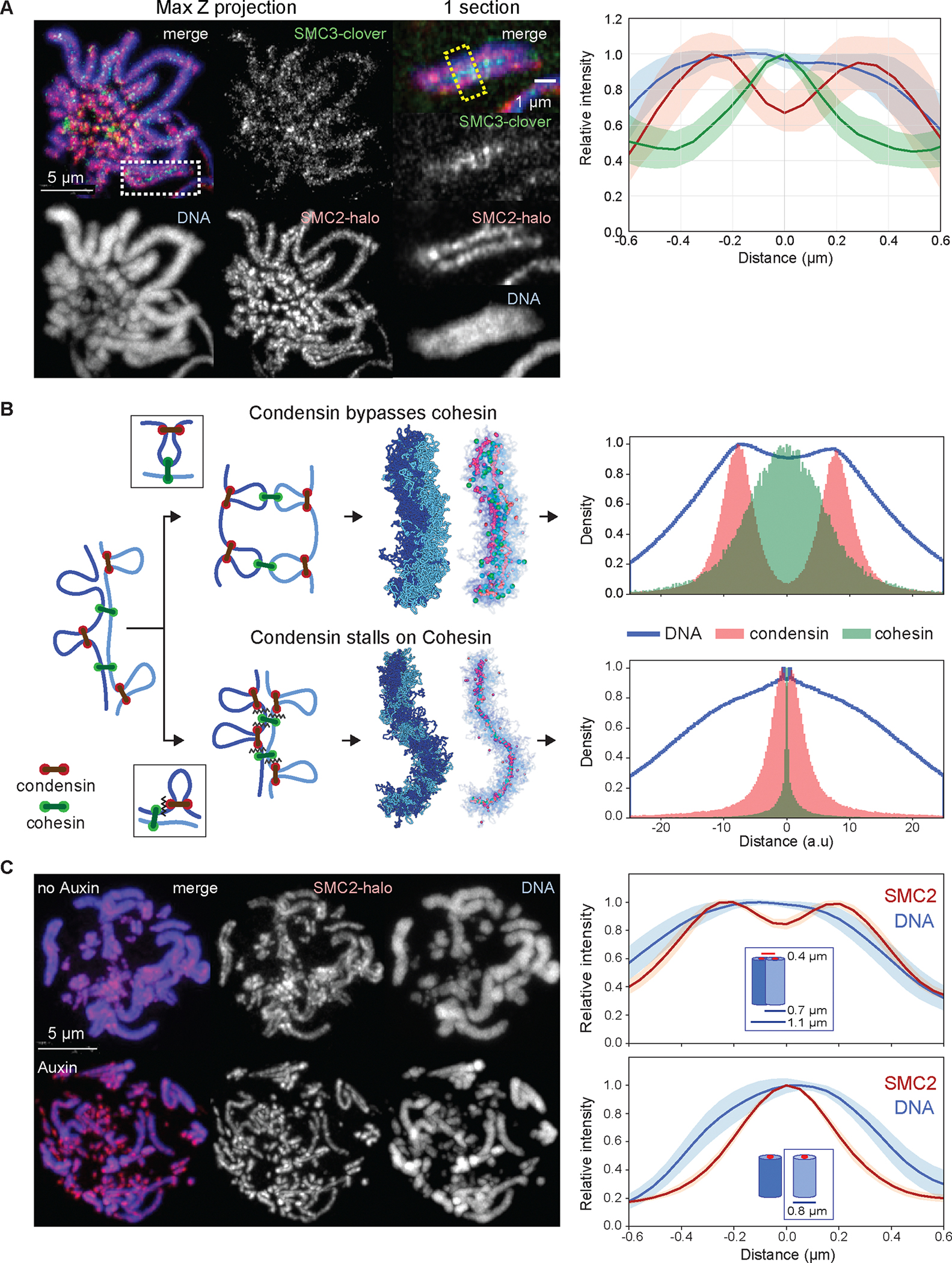
Condensin bypasses cohesin to establish separated but cohesive sister chromatids. (**A**) Localization of cohesin (SMC3) and condensin (SMC2) in prometaphase knock-in SMC3-clover/SMC2-Halo CDK1^as^ cells 30 minutes after release from G_2_ block. JFX646-Halo (1/1,000) was added to visualize SMC2-Halo. To remove chromatin-unbound SMC3, cells were fixed with 4% formaldehyde plus 0.5 % Triton. (left), Maximum projection of the full stack of z-sections. (right), Zoom of region in the white rectangle (single z-section). Line scans across chromosomes in the single z-section images (yellow box) were used to quantify the relative fluorescence intensities of SMC3-clover, SMC2-Halo, and DNA (plot to the right). Plots were centered around the position with the highest SMC3 intensity. Data obtained with 10 chromosomes each for two replicate experiments were averaged. Shaded envelopes around main lines represent standard deviations. (**B**) Polymer models of chromatid compaction of pairs of cohesive sister chromatids through condensin-mediated loop extrusion. Left: two mechanisms of interactions between condensin and cohesive cohesins are modeled (top: condensins bypassing cohesive cohesins; bottom: condensins stalling at cohesive cohesins). Polymers of sisters are shown in shades of blue, cohesive cohesins in green, and extruding condensins in red. Middle: simulated outcomes of configurations of sister chromatids obtained with bypassing (top) or stalling models. Right: histograms present the localization of cohesin, condensin, and DNA for cross-sections of pairs of sister chromatids (as in panel A, right). (**C**, left) Representative images of prometaphase SMC3-AID/SMC2-Halo cells without or with auxin treatment to remove cohesin prior to mitotic entry. Cells at t= 30 min after release from G_2_ block were fixed with formaldehyde and SMC2-Halo was visualized with JFX549-Halo (1/10,000). DNA was stained with Hoechst 33542. Max projections of z sections are shown; scale bar = 5 μm. (right) Line-scan quantification of relative fluorescence intensities of DNA and SMC2 across pairs of sister chromatids (no auxin) or single chromatids (+ auxin). Positions with lowest SMC2 intensity (no auxin, marks the point where sister chromatids touch) or highest (auxin, the midpoint of a single chromatid) were aligned in the middle. n = 10 chromosomes for each condition in two replicate experiments. Shadow colors show standard deviation. The calculation of the chromatid/chromosome width is described in the Method section.

Cohesin did not co-localize with condensin on prometaphase chromosomes, yet localized all along the chromosome arms between the sister chromatids, away from the condensin axis of each chromatid (Fig. 3A - see also Rhodes et al. (50)). This distribution is consistent with cohesive cohesin (51) forming links between the distal portions of chromatin loops anchored by axial condensin (Fig. 3A) (51).

### Condensin must bypass cohesive cohesins

We developed polymer models to explore possible interactions between condensin and cohesive cohesin. In our model, two polymers represent the sister chromatids in G_2_ linked by cohesive cohesin complexes (for simplicity we omitted extrusive cohesins) (32). We explored two possible interactions once condensins start extruding loops during prophase (Fig. 3B): 1) bypassing, where condensins step over cohesive cohesins, and 2) stalling and/or pushing, where condensins accumulate at cohesive cohesins or push them ahead along the DNA.

The two mechanisms gave rise to different organizations of sister chromatids. Firstly, condensins that cannot bypass cohesive cohesin stall at those sites, resulting in tight colocalization of both SMC complexes, with sister chromatids sharing a single axis, in clear disagreement with microscopy (Fig. 3B). In contrast, if condensins can bypass cohesive cohesins, the two sister chromatids would individualize, with each forming a bottlebrush of loops having condensins enriched along its axis and cohesins linking loops of the two sisters at the interface connecting the two bottlebrushes (Fig. 3B). This distribution is in agreement with cohesin and condensin localisation seen in cells (Fig. 3A).

We considered the alternative model that sister chromatid axes could separate even if condensin does not bypass cohesive cohesin simply as a result of the prophase decline in cohesive cohesin levels on chromosome arms. The following observations make this model less likely. Firstly, during prometaphase, we clearly detect cohesin all along the arms at the interface between sisters (most likely cohesive cohesin - Fig. 3A). Secondly, our proteomics data show that levels of cohesin exceed those of condensin II well into prometaphase. Thirdly, to test this model directly, we performed two further experiments. We created a Wapl-AID cell line in which levels of Wapl become undetectable after addition of 5Ph-IAA. This results in a substantial increase in cohesin levels on chromosomes throughout mitosis. Under these conditions we clearly observe the separation of condensin axes in the middle of each sister chromatid flanking cohesive cohesin (Fig. S3). Thus, increasing cohesin levels does not prevent sister chromatid axis separation. We also performed a simulation in which cohesin levels were decreased in the absence of condensin bypassing. In this scenario, sister axes can separate at regions without cohesin, but at locations where cohesive cohesin remains, condensins co-localize with cohesin. During prometaphase, we do not observe such co-localization. Considering this evidence from multiple orthogonal approaches, we propose that when condensin II encounters cohesive cohesin, it bypasses it. (9)(7)

### Cohesin depletion significantly alters mitotic chromosome organization

As expected, depletion of cohesin in G_2_ resulted in sister chromatid separation in the subsequent prometaphase (Fig. 3C). Quantification of chromatin-associated proteins by ChEP confirmed that the core cohesin subunits (SMC1/3 and Rad21) were all depleted from chromatin (Fig S2A). In contrast, the amounts and localization of other chromatin-associated key scaffold proteins, including condensins, TopoIIα, and KIF4A, resembled those of control cells when visualized by conventional and super-resolution light microscopy (Fig. S2C, Fig. S7, S8) (46). Cohesin depletion in late G_2_ did not alter the kinetics of mitotic entry (Fig S1D).

**Fig. 4.**
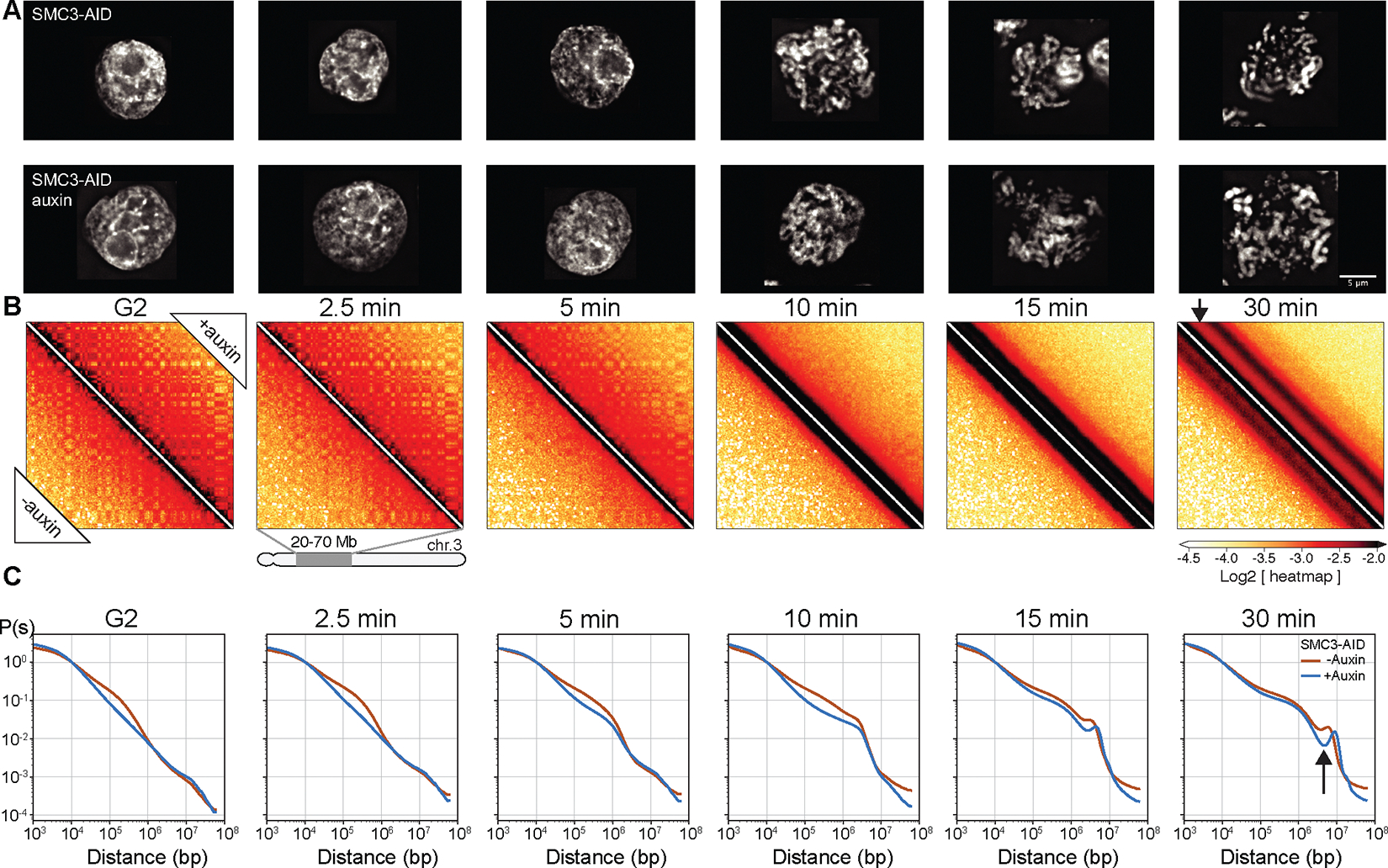
Cohesin impedes helical coiling of mitotic chromosomes. (**A**) Representative images of DAPI-stained SMC3-AID cells used to prepare Hi-C samples. Top row: cells from control culture (no auxin treatment). Bottom row: cells from culture treated with auxin during the G_2_ block and release into mitosis. Scale bar = 5 μm. (**B**) Hi-C contact maps for SMC3-AID CDK1^as^ cells treated as in panel A. Chromosome 3, position 20–70 Mb is shown. Compartmentalization (checkerboard pattern in Hi-C maps) was stronger in G_2_ cells, and the second diagonal band appeared sharper, and positioned at larger genomic distance in prometaphase, in SMC3-depleted CDK1^as^ cells (black arrow) compared to those of non-depleted control cells. (**C**) Quantification of Hi-C data shown in panel B: contact frequency *P* plotted as a function of genomic separation (*s*). *P*(*s*) curves reveal position and prominence of the second diagonal band visible in Hi-C maps (arrow) from all chromosome arms greater than 100 Mb.

G_2_-arrested DT40 cells depleted of SMC3 lost cohesin-mediated features (TADs, dots, and stripes), and exhibited much stronger compartmentalization (47, 48)(Fig. 4A, 4B, left panel; 5B, middle panels). Global chromatin organization revealed by *P*(*s*) [contact probability *P* as a function of genomic separation (*s*)] changed upon SMC3 depletion. The characteristic “shoulder” at ~100 kb indicative of cohesin-mediated loops (52, 53)) was lost. Furthermore, the slope of *P*(*s*)~*s*^−1^ revealed a fractal globule folding of chromatin over more than two decades of genomic separation (*s*= 10^4^-10^7^ bp) (Fig. 4C, Fig. S4B).

By prometaphase, substantial differences in Hi-C interaction maps were observed. Maps from wild type prometaphase cells revealed a second diagonal, reflecting a helical loop arrangement (6, 54) (Fig 4C, lower left of each panel). In cohesin-depleted cells, this second diagonal was substantially more prominent (Fig 4C, upper right), confirming that it reflects helical organization of individual chromatids and is not due to inter-sister interactions. Furthermore, the second diagonal moved to larger genomic distances (from ~6 to ~8 Mb) (Fig. 4C). Thus, the normal helical pattern is altered by the presence of cohesin-mediated interactions.

SMC3-depleted late prometaphase chromosomes (t= 30 min) were wider and shorter than chromosomes in control cells (Fig. 4A, 6C–E), consistent with the increased size of each helical turn revealed by Hi-C (Fig. 4C). Loose association between sister chromatids remained, probably due to residual catenations (55–58). Wild-type chromosomes with two tightly cohesed sister chromatids had an overall width of 1.1 μm, consistent with a sister chromatid width of 0.55 μm. However, the diameter of individual chromatids measured in the absence of cohesin is 0.8 μm (Fig. 3C, 6C). Thus, cohesive cohesin apparently compresses the paired sister chromatids by about 0.3–0.5 μm.

Together, these data show that cohesin has a previously unappreciated role in modulating condensin-driven mitotic chromosome architecture.

### Cohesive, but not extrusive cohesin, shapes mitotic chromosomes

**Fig. 5.**
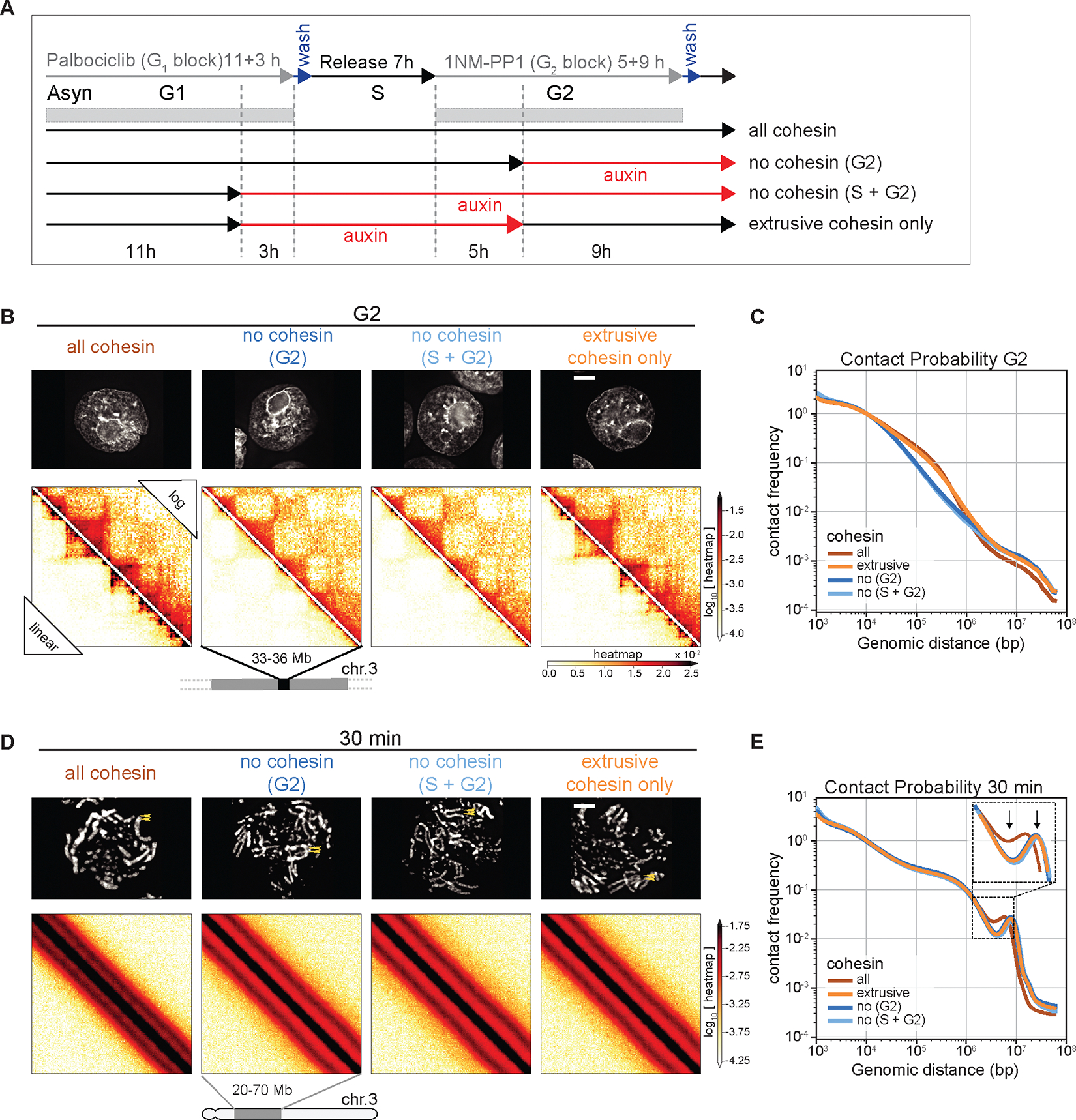
Cohesive and not extrusive cohesin impedes helical coiling of mitotic chromosomes. (**A**) Double synchronization procedure for SMC3-AID CDK1^as^ cells. Cells were collected and cross-linked in G_2_ or at 5, 15, 30 minutes after release from G_2_ block. FACS-sorted cells were used for subsequent analysis. GFP-positive cells (labeled “all cohesin” and “extrusive cohesin only”) contained those respective SMC3 populations. In GFP -negative cells (“no cohesin, G_2_” and “no cohesin, S + G_2_”) SMC3 was depleted in the indicated cell cycle phases. (**B**, **D**) Images of DAPI-stained SMC3-AID cells used to prepare Hi-C samples and corresponding Hi-C interaction maps at G_2_ (B) and t= 30 min (D). Scale bar = 5 μm. Hi-C interaction maps are shown in log (top right) and linear scales (bottom left). (**C**, **E**) Contact frequency *P*(*s*) vs. genomic separation (*s*) for maps shown in B and D. Inset in (**E**) shows the magnified view of the region of *P*(*s*) corresponding to the second diagonal band visible in Hi-C interaction maps. Arrows point the difference in the position and prominence of the second diagonal band.

We next determined whether the unexpected role of cohesin in mitotic chromosome architecture is carried out by cohesive or extrusive forms of cohesin, or both. Based on observations that cohesion is only established during S-phase in yeast (51), we established a double synchronization protocol combining the CDK4/6 inhibitor palbociclib with 1NM-PP1 to obtain four populations of G_2_ and mitotic cells with distinct cohesin states (Fig. 5A):

1. Cohesin present during S and subsequent mitosis (both cohesive and extrusive cohesin present during mitosis);
2. Cohesin present during S and absent during subsequent G_2_ / mitosis;
3. Cohesin absent during S and also during subsequent G_2_ / mitosis;
4. Cohesin absent during S and restored during subsequent G_2_ / mitosis (extrusive cohesin only during mitosis).

All 4 cultures progressed through S phase and entered mitosis with essentially identical kinetics, forming rod-shaped chromatids after 1NM-PP1 washout (see Fig. 5D, S5D). This confirms earlier observations that cohesin is not essential for normal S phase progression, entry into mitosis or for the formation of rod-shaped chromosomes.

Control cultures 1–3 all behaved as predicted (Fig. 4). In G_2_, TADs and dots (CTCF-CTCF loops) observed in Hi-C maps in controls were absent when cohesin was depleted (Fig. 5B,C). As expected (51), cells in condition 4 (cohesin absent during replication) failed to re-establish sister chromatid cohesion when cohesin was restored in G_2_ (Fig. 5D,E, S5D). In contrast, TADs and dots reformed when cohesin was restored during G_2_ (Fig. 5B). Thus, extrusive, rather than cohesive cohesin, is important for formation of TADs, dots and stripes in G_2_.

By late prometaphase (at t= 30 min), Hi-C data obtained from all three cultures lacking cohesive cohesin (cultures 2, 3, 4) were indistinguishable regardless of whether extrusive cohesin was present. Specifically, the second diagonal appeared much more pronounced (Fig. 5D,E) and ran at a larger distance compared to control cells (see Fig. 4). Furthermore, the separated sister chromatids were shorter and wider than chromosomes in control cells in all cultures lacking cohesive cohesin, regardless of extrusive cohesin re-loading in G_2_ (Fig. S5).

Thus, cohesive, not extrusive, cohesin alters the helical conformation and dimensions of prometaphase chromosomes, presumably by linking sister chromatids and limiting the ability of each sister to form an independent helical condensin loop array.

### Roles of individual condensin complexes in mitotic chromosome assembly

Previous studies had examined cell lines lacking condensin I, II, but all in the presence of cohesin (6, 37, 59–62). We therefore created three additional cell lines to disentangle the roles of single SMC complexes in chromosome assembly. SMC5/6 complexes were omitted here as their depletion in mitosis was reported to have no effect on mitotic chromosome structure (63). These allowed us to analyze the following conditions:

1. Condensin II only - SMC3-AID/CAP-H-AID cells allow co-depletion of cohesin and condensin I;
2. Condensin I only - SMC3-AID/CAP-H2-AID cells allow co-depletion of cohesin and condensin II;
3. No cohesin or condensin - SMC3-AID/SMC2-AID cells allow co-depletion of cohesin and both condensins.
4. Condensin I & II, no cohesin - SMC3-AID cells provide a baseline with both condensin I and II active, and cohesin depleted.

In G_2_, microscopy (DNA staining) and Hi-C data for all the above cell lines lacking cohesin, were indistinguishable from one another (Fig. S6). Hi-C maps showed loss of cohesin-mediated features (TADs, dots, and stripes), *P*(*s*) plots lacked the characteristic shoulder at s~100 kb produced by cohesin-mediated loops (Fig. 5C, Fig. S4). Thus, we confirm and extend earlier reports that condensin I and II lack major roles in G_2_ chromatin organization (6, 64).

**Fig. 6.**
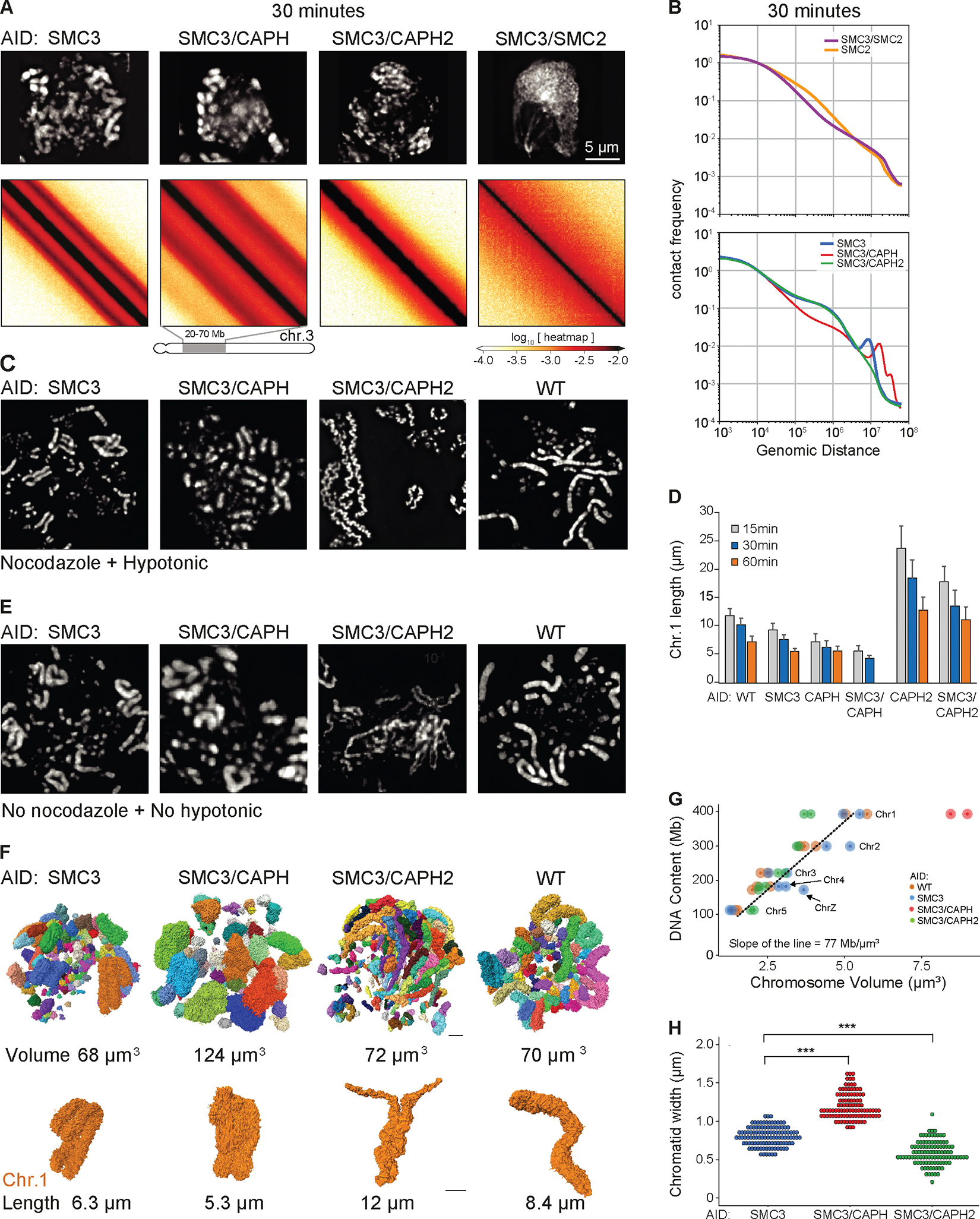
Structure of chromosomes assembled by single SMC complexes. (**A**) SMC3-AID, SMC3-AID/CAP-H-AID, SMC3-AID/CAP-H2-AID, SMC3-AID/SMC2-AID cells treated with auxin during G_2_ arrest were released into mitosis and DAPI-stained cells imaged at t= 30 min (prometaphase). Scale bar = 5 μm. Hi-C was performed on the same cultures at t= 30 min. Hi-C interaction maps for a portion of chromosome 3 is shown for each cell line. (**B**) Plots showing contact frequency *P* as a function of genomic separation (*s*) for Hi-C data shown in A, as well as for SMC2-depleted cells. (**C**) Chromosome spreads of DAPI-stained WT and SMC3-AID, SMC3-AID/CAP-H-AID, SMC3-AID/CAP-H2-AID cells plus auxin. Cells in G_2_ block were treated with 0.5 μg/ml nocodazole for 30 minutes prior to release into mitosis for an additional 30 min. Cells were harvested and hypotonically swollen with 75 mM KCl for 10 minutes prior to ice-cold methanol-acetic acid (3:1) fixation and spreading. (**D**) Length of the longest chromosome (Chr 1) of each cell line treated with auxin in the previous G_2_ measured using cells processed at t= 30 min post release from G_2_ (prometaphase) as shown in panel C. n ≥16 chromosomes or chromatids measured in 3 independent experiments. Average and standard deviation are shown. (**E**) As in (C) but cells were rinsed with PBS prior to ice-cold methanol-acetic acid (3:1) fixation and spreading (no nocodazole, no hypotonic treatment). (**F**) 3D Reconstruction of prometaphase chromosomes of WT, SMC3-AID, SMC3-AID/CAP-H-AID and SMC3-AID/CAP-H2-AID cells treated with auxin in G_2_, and then released into mitosis (t= 30 minutes). Each image represents a 3D reconstruction of an entire mitotic cell obtained by SBF-SEM. Each chromosome is represented as a different color. Chromosome 1 of each cell line is shown in orange. The total volume of the chromosomes and the length of chromosome 1 are indicated. Scale bar = 2 μm. (**G**) Correlation between DNA content vs. chromosome volume, derived from images shown in panel G. Chromosomes 1–5 and Z of each cell line are annotated in the graph. The slope of the line is calculated for WT, SMC3-AID, and SMC3-AID/CAP-H2-AID. (**H**) Chromatid width quantification of large chromosomes in each mutant. Each point represents a measurement, and 10 measurements were taken per chromosome. n= 10 chromosomes, *** p<0.001.

In contrast, mitotic chromosome morphologies and Hi-C data obtained after co-depleting combinations of SMC complexes in G_2_ cells exhibited substantial differences (Fig. 6, A and B). Mitotic chromosomes lacking condensins had no compact rod-shaped morphology and appeared clumped together at late prometaphase. Centromeric chromatin was often stretched by kinetochore-microtubule interactions (41). This morphology was unaltered by depletion of cohesin (e.g. SMC2-AID versus SMC3-AID/SMC2-AID). No second diagonal band was observed in Hi-C analysis of these cells in prometaphase (Fig. 6B).

The contact frequency *P*(*s*) curve for SMC2-AID cells (with cohesin, but without condensin) shows the presence of cohesin-mediated loops, seen as a characteristic shoulder at s~ 100 kb that persists deep into prometaphase (t= 30 min) (Fig. 6B). This feature is absent from SMC3-AID/SMC2-AID cells (no cohesin, no condensin). Thus, cohesin-mediated loops persist in the absence of condensin.

Prometaphase chromosomes formed by condensin I are long and “wiggly” compared to WT (47, 65). This phenotype was exaggerated in chromosome spreads when cohesin was also absent (Fig. 6C - SMC3-AID/CAPH2-AID). In contrast, these same chromosomes when fixed under more native conditions without nocodazole or hypotonic treatment were disorganized with highly variable width (Fig. 6E), and resembled chromosomes in 3-dimensional reconstructions of whole cells imaged by electron microscopy (Fig. 6F). Thus, (and often overlooked in other studies) the structural parameters of chromosomes observed by traditional microscopy methods are extremely sensitive to the exact protocols used for sample preparation. Despite this variability, chromosomes assembled by condensin I only are reproducibly shorter than the same chromosomes assembled in the presence of cohesin (Fig. 6D, Fig. S7A).

Chromosomes formed by condensin II only (lacking cohesin) were rod-shaped and exhibited a relatively “dumpy” and fragile appearance by light microscopy (SMC3-AID/CAPH-AID in Fig. 6A,C,E). As previously described by others (37, 59, 60), these chromosomes are shorter and fatter than chromosomes with both condensins (CAPH-AID vs WT and SMC3-AID/CAPH-AID vs SMC3-AID in Fig. 6D). Consistently, the additional removal of cohesin made chromosomes even shorter and less well defined in shape (CAPH-AID vs SMC3-AID/CAPH-AID in Fig. 6D, S7A).

The lack of cohesin also exaggerated differences in the Hi-C data for condensin I-only and condensin II-only chromosomes. Hi-C data from condensin I-only chromosomes lack the second diagonal, indicating the absence of helical organization (Fig. 6A, S4D). This confirms our previous conclusion that condensin II is required to form a helical array of chromatin loops (6). Hi-C data indicate that these chromosomes consist of an array of ~100 kb loops reflected by the shoulder on the *P*(*s*) curve for s~100 kb (Fig. 6B, S4D). This is followed by a region of steady decay between 2–8 Mb with a slope of −1.5, indicative of the array adopting a random walk (66). This behavior is consistent with the long wiggly chromosomes observed in microscopy of native spreads (Fig. 6A,E,F).

Condensin II-only chromosomes yielded Hi-C maps in which the second diagonal band was flanked by robust third, and fainter 4^th^ diagonal bands. The position of the second diagonal appeared around 16 Mb, about twice that of chromosomes formed by condensin I and II. This re-location of the second diagonal to larger genomic distances revealed that each helical gyre contains more DNA, consistent with the shorter and wider morphology of these chromosomes, as compared to the same chromosomes in the presence of cohesin (compare Fig. 6A,C,E with Fig. 6D,H).

The second diagonal in Hi-C has been explained by interactions between adjacent gyres of chromatids in a helical configuration (6). The third and fourth diagonals were positioned at multiples of 16 Mb: 32 and ~40–50 Mb, suggesting that chromatin in one gyre interacts with chromatin two or three gyres above or below. Careful inspection revealed that a very weak third diagonal band was also visible in Hi-C data from cells lacking condensin I but containing both condensin II and cohesin (Fig. S4E). This strongly suggests that the additional diagonals are caused by condensin II-mediated chromatin loops, and that the presence of cohesin weakens these helical features in Hi-C analysis.

### Measurement of chromosome parameters for polymer modeling

Creation of accurate polymer models to explain our Hi-C data requires precise volumes and shapes for the various mutant chromosomes. Such measurements cannot be obtained from light microscopy due to (1) variations in chromosome morphology as a result of the spreading protocol, (2) differences in cell fixation and (3) the limited resolution of the light microscope. We used Serial Block Face Scanning Electron Microscopy (SBF-SEM) coupled with a labeling method that selectively enhances the contrast of DNA (Methods).

The total volume of all chromosomes in WT, SMC3-AID, and SMC3-AID/CAPH2-AID cells was similar, with values of 68 μm^3^, 72 μm^3^, and 70 μm^3^, respectively. Chromosomes 1 – 5 and Z can be unambiguously identified in WT and SMC3-AID cells. Knowing the volume and DNA content of each yields an average DNA density of 77 Mb/μm^3^ (Fig. 6G, Movies S1–S8). However, the fuzzy-looking chromosomes in SMC3-AID/CAPH-AID mutants had a substantially larger volume of 124 μm^3^ (Fig. 6F), corresponding to an average DNA density of 44 Mb/μm^3^ (Fig. 6G).

### Structural models for the roles of condensins in mitotic chromosome formation

We modeled the folding of individualized mature (t=30 min) chromatids formed by single condensin complexes. We simulated a 100 Mb chromatid at a single nucleosome resolution. which was folded by condensins into consecutive loops and packed into a cylindrical body. The resulting chain of loops was weakly “nudged” to follow a helical path (see the Materials and Methods). The model used two experimentally measured parameters, the volume density (Fig 6G) and the helical turn (from Hi-C), and four free parameters: average sizes of loops and gaps between them, helical pitch, and Hi-C contact radius. We found that chromatid morphology and *P*(*s*) curves were sensitive to all 4 parameters and therefore by fitting experimental data, *P*(*s*) and reproducing morphologies from microscopy, we can estimate the values of those parameters. (Fig. S9A,B)

**Fig. 7.**
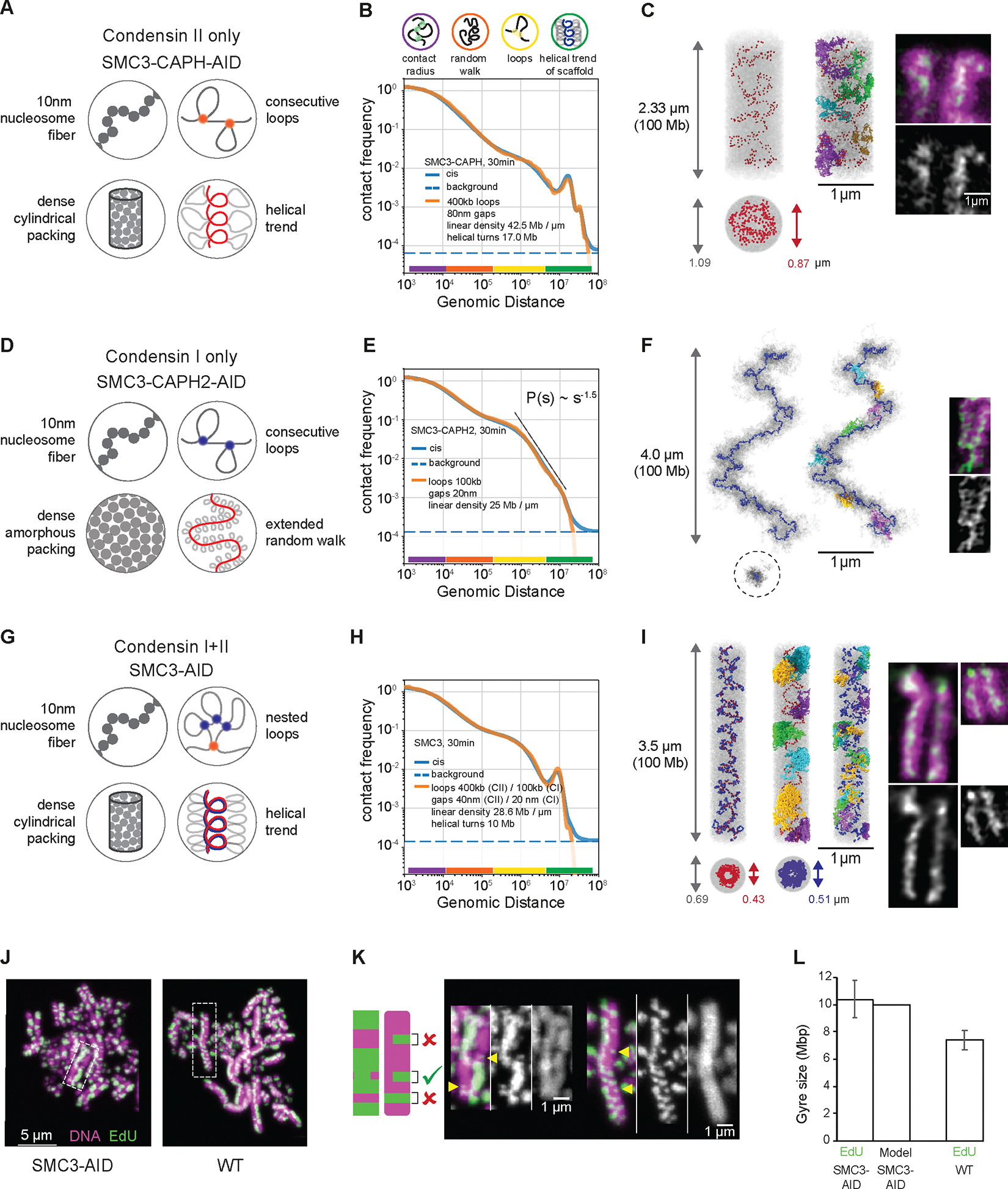
Polymer modeling reveals the internal organization of chromosomes built by single condensin complexes. (A-C) Model of SMC3-AID/CAPH-AID (condensin II-only) prometaphase (t=30 min) chromosomes. (**A**) The four modeling assumptions. (**B**) Contact frequency *P* as a function of genomic separation *s* for the best-fitting model (orange line, model parameters are listed in the plot), in comparison to experimental data (blue line). Dotted line indicates upper limit of the background interaction frequency in experiments, estimated from the average inter-chromosomal interaction frequency. The colored circles (top) and color bars (bottom) indicate levels of genomic organization and their characteristic sizes in base pairs. (**C**) Left: Simulated chromosome conformation in one modeling replicate in longitudinal projection (top) and a cross-section (bottom). DNA is shown in gray, condensins II shown as red spheres. Gray and red arrows indicate diameter of the chromatid and the condensin scaffold respectively. Middle: Same, but with a few selected loops stained in different colors. Right: A microscopy image of SMC3-AID/CAP-H-AID/Halo-CAP-H2 chromosome. Halo-JFX549 (green) is added to the medium >30 min prior to 1NM-PP1 washout to stain Halo-tagged proteins. Cells were treated with 1NM-PP1 for 13 h and fixed with formaldehyde 30 minutes after 1NM-PP1 washout, and DNA was stained with Hoechst (magenta). Scale bar = 1 μm. (**D-F**) Same as (A-C) but for SMC3-AID/CAPH2-AID (condensin I-only) prometaphase chromosomes. In (F), the two left images show positions of condensins I as blue spheres. In the middle image, a few selected loops are colored. Right: Microscopy images of SMC3-AID/CAP-H2-AID/SMC2-Halo chromosomes, stained with DAPI and Halo. (**G-I**) Same as (A-C) but for SMC3-AID (condensin I+II) chromosomes. In (I), SMC3-AID/SMC2-Halo (left) shows DNA plus all condensins, SMC3-AID/Halo-CAP-H2 (right) shows DNA plus only condensin II. (**J**) SMC3-AID and WT chromosome spreads in which EdU is incorporated into one sister chromatid after two cycles of DNA replication. The harlequin appearance is caused by sister-chromatid exchanges. EdU (green), DNA (magenta). Scale bar = 5 μm. (**K**) Enlarged images from (J). EdU (green), DNA (magenta). Scale bar = 1 μm. Cartoon shows the criteria used to select partial exchanges to measure the height of an EdU-labeled gyre. Arrow heads indicate gyres matching criteria (**L**) Size of gyre calculated from EdU height in wild type and SMC3-AID (cohesin depleted) sister-chromatid exchanges and the average gyre size used for modeling. n=4, total 92 measurements (SMC3-AID), n=3, total 81 measurements (WT).

For condensin II-only chromosomes (SMC3-AID/CAPH-AID), our best-fit model reproduced the Hi-C *P*(*s*) across ~4.5 decades of genomic separation (Fig. 7B). In this model, condensins II formed 400 kb loops, with adjacent condensins separated by 80 nm gaps, and organized into an irregular helix (Fig. 7C) with a pitch of 400 nm containing 17 Mb per helical turn. Individual chromosomes clearly differ in the 3D position of condensins and the folding of individual loops, reinforcing the notion that there is no single structure of mitotic chromosomes (Fig. S9C,D).

Best-fit models reproduced both the third diagonal at 34 Mb and weak fourth diagonal at ~40–50 Mb observed in Hi-C. These third and fourth diagonals represent contacts between large loops separated by two and three helical turns (Fig. S9E). This explains why these extra diagonals run at multiples of the 17 Mb second diagonal. Thus, even weakly imposed helicity of bottlebrush chromosomes can naturally produce multiple periodic diagonals visible in Hi-C.

Condensin I-only chromatids (SMC3-AID/CAPH2-AID) required a fundamentally different model, as (a) they do not form regular cylinders, but rather “wiggling noodles” with variable width along the chromatid (Fig. 6C,E,F) and (b) Hi-C shows no periodic patterns (i.e., no extra diagonals) and P(s) follows ŝ-1.5 for s~2–8 Mb. Together, these observations suggest that the bottlebrush of loops follows a random-walk trajectory at these length scales. Thus, in modelling, we omitted a cylindrical constraint and instead allowed the chromatid to fold into a random walk, stretched to a specified end-to-end linear distance (Fig. S10A). To maintain the high chromatin density, we imposed periodic boundary conditions (Fig. 7D).

The best-fit model accurately reproduced the Hi-C across over four decades of genomic separation (Fig. 7E, S10B, Methods) and was consistent with microscopy. In the model, condensin I folds chromosomes into bottlebrushes of ~100 kb loops, separated by 20 nm gaps (Fig. S10B). At the scale of multiple loops, this bottle-brush followed a random walk weakly stretched to ~4 μm per 100Mb and did not form a helix (Fig. 7F, S10B,C).

We could reconstruct chromatids formed by both condensins (SMC3-AID) by combining the two models described above. In this combined model, longer condensin II loops were split into a second layer of shorter nested loops by condensins I (Fig. 7G–I, Methods) (6). Using the same parameters as models above (with only minor changes in gap sizes), the resulting integrated model accurately reproduced experimental Hi-C across >4 decades of genomic separation (Fig. 7H, S10D, Methods), and generated a narrow chromosome scaffold as observed with anti-KIF4A (Fig. S8). The loop sizes (400 kb for condensin II, 100 kb for condensin I) were consistent with the results of quantitative proteomics (Fig. 1D; 2–3 condensins II and ~10 condensins I per Mb). This integrated model suggests that the two condensin complexes function additively on the same chromatin substrate at the same time.

We validated these models by comparing their structural predictions against two independent approaches, light and electron microscopy. Firstly, the chromatid width, length, and 3D (“crow flies”) distance between chromatid ends observed in chromosome spreads and in intact cells correspond closely to the corresponding distances predicted by the models (Table S3). Secondly, it has been reported that gyre size can be estimated by measuring the dimensions of a class of sister chromatid exchange events detected by EdU labeling ((54) and Methods, Fig. 7J–K). Using this approach allowed us to obtain independent measurements of gyre sizes. The height of these partial exchange events measured experimentally corresponds nearly exactly to the pitch of the helix in the model calculations for SMC3-depleted cells and to the genomic spacing of the second diagonal in Hi-C of SMC3-depleted and wild-type cells (Fig. 7J–K, Fig. S8, Tables S4 and S5).

Finally, we used polymer modeling to test the effect of inter-sister cohesion on the internal structure of individual chromatids. To simulate the effects of inter-sister connections on the loop conformations, we forced the tips of several randomly chosen loops to be positioned on one side of the cylinder, where they contact the other sister chromatid (Supplemental Materials). These models showed that cohesion at a realistic frequency of ~1 / 1Mb reduces but does not fully erase the second Hi-C diagonal (Supp Fig 10H), consistent with the Hi-C data (Fig. 4, 5). This confirms that cohesed chromatids can coil despite being linked.

### In vivo estimation of the extrusion speed

Our system with highly synchronous entry into prophase in the presence of a single extruder, alongside models that infer loop sizes from Hi-C data, provides a unique opportunity to estimate the speed of extrusion of individual condensins in vivo.

Loop sizes can be estimated from the *P*(*s*) curves. While in interphase, where the loop density is low, the loop size corresponds to genomic distance at the peak of the *P*(*s*) derivative (52, 53), its position in the dense mitotic loop array is harder to infer. Our models show that the loop size defined in a model has a characteristic position somewhat to the left of the peak on its *P*(*s*) derivative (Fig. S9A, arrowhead). This allows us to estimate loop sizes as a function of time.

For chromosomes formed only by condensin II, at t= 2.5 min, *P*(*s*) reveals a lower density of loops and suggests an average loop size of ~200–300 kb (Fig. S4C). We thus estimate the speed of extrusion for individual condensin II complexes as 200–300 kb/2.5 min, giving an extrusion speed in vivo of ~1.3–2 kb/sec. At t= 5 min, the loop size is 400 kb (Fig. S4C), again yielding an extrusion speed of ~1.3 kb/sec. These values are approximations because we cannot know the exact time when condensin II is activated during reversal of the 1NM-PP1 block. In an independent approach, by measuring the change in loop size between t= 2.5 and 5 minutes in a single time course, we estimate the extrusion speed to be at least (400 minus 200–300) = 100–200 kb in 2.5 min, or 0.5–1.3 kb/sec.

Microscopy provides a third way to estimate the speed of extrusion by condensin II. By t~10 minutes, chromosomes in SMC3-AID/CAPH-AID cells acquire the flexible rod-like shape that is characteristic of a dense loop array with no gaps (Fig. S6C). Theory shows that to close most gaps, condensins need to extrude ~4–5 times the average loop size (Methods). Using the average loop size from the *P*(*s*) at t= 10 minutes (400 kb), we calculate an extrusion speed of 400 kb*(4–5) / 600 sec=2.5–3 kb/sec.

Altogether, our analysis in SMC3-AID/CAPH-AID cells yields an extrusion velocity of condensin II as 1–3 kb/sec in living DT40 cells.

This approach also allows us to quantify loop extrusion by condensin I. In condensin-I only chromosomes, the *P*(*s*) reveals loop formation between t= 5 and 10 minutes, i.e., even before nuclear envelope breakdown (Fig. S4D). Together with the ChEP data, this argues for the presence of active nuclear condensin I at these early time points (60). Comparison of the *P*(*s*) for t= 2.5 minutes (no loops) and 7.5 minutes (~300–400 kb loops) (Fig. S4D), reveals that these nuclear condensins I extrude loops at ~1 kb/sec.

We can also directly observe the formation of nested loops by condensin I in the presence of condensin II in SMC3-AID cells. We observe formation of large (400 kb) loops before NEB (by t= 10 minutes, largely by condensin II) (Fig. S4B). The loops then abruptly become smaller (~100 kb) upon NEB when the bulk of condensin I gets access to chromosomes (Fig. S4B). This drop in the loop size strongly supports a nested loop organization of the mitotic chromosome where each ~400 kb condensin II loop is split into several ~100 kb condensin I loops.

### Helical dynamics

Analysis of the *P*(*s*) curves at different time points yields information on the period of the chromatid helix as revealed by the second diagonal, i.e., the first peak on the *P*(*s*) curve (Fig. S9A, S4A,B,D). In wild type cells, the period grows from 4.0 to 6.1 Mb between t= 15 and 30 minutes (~2.3 kb/sec). Helix growth correlates with an overall shortening of the chromosome (Fig. 6D). In SMC3-depleted cells, the growth of the helix over this period rises to ~3.5 kb/sec. For chromosomes built solely by condensin II, the period changes from ~6.6 Mb to 16.4 Mb between 15 and 30 minutes. This yields the growth rate of 650 kb/min = 10.9 kb/sec over 15 minutes, i.e. almost an order of magnitude higher than the speed of condensin loop extrusion. Moreover, the *P*(*s*) between these two time points shows almost no change in average loop size. Therefore, the growth of the helical gyre, though dependent on condensin II, appears to be driven by processes other than extrusion of the 400 Kb loops that has been completed by the end of prophase. This additional role of condensin II in driving helix formation is restrained by cohesive cohesin and condensin I (Table S5).

## DISCUSSION:

During prophase, two types of cohesin and two types of condensin act on chromosomes. Our study reveals how collisions between these complexes are resolved, defining three “rules of engagement” when an actively extruding condensin complex runs into another SMC complex: bypassing, collision-facilitated removal, and stopping/blocking. (66). Application of such rules explains how the local action of SMC complexes and their interactions leads to the formation of condensed mitotic chromatids that are cohesive via interactions between their loops. Furthermore, as we discuss below, our temporal analysis in DT40 cells entering highly synchronous mitosis also allows us to calculate the speed of loop extrusion by condensin complexes in living vertebrate cells.

### Rules of engagement when condensin and cohesin encounter each other

#### Rule 1: Condensins bypass cohesive cohesins

Our data provide strong evidence for the ability of condensins to bypass cohesive cohesins in vivo. Cohesins that connect arms of sister chromatids end up at the tips of condensin-extruded loops by t= 30 min into prometaphase. Since those cohesins were loaded during S-phase, before condensin-mediated extrusion begins in prophase, they must be bypassed to end up at the tips of the condensin loops, as shown by our simulations. If condensins failed to bypass cohesive cohesins but instead either pushed or stalled on them, then cohesins would accumulate at the bases of condensin loops. The result would be a structure with two sister chromatids connected at the bases of the condensin loops by cohesins, thus forming a single axis, contrary to what is observed. However, a single axis could form transiently if condensins pause before bypassing cohesive cohesins. The ability of loop-extruding SMCs to bypass each other (9) or large DNA-bound obstacles (67) has been observed in single-molecule experiments, and in vivo in bacteria (7, 68). In contrast, extrusive cohesins appear not to bypass cohesive cohesins in *S. cerevisae* (69). Here we demonstrate using multiple orthogonal approaches that vertebrate condensins can bypass other SMC complexes, and the structures that they tether. When condensins bypass cohesive cohesin, they are effectively bypassing the entire other sister chromatid.

#### Rule 2. Condensins mediate removal of extrusive cohesins

In contrast to the situation with cohesive cohesin, encounters between condensin and extrusive cohesin are apparently resolved by removal of cohesin (e.g., the prophase pathway (25, 26)). Our Hi-C data indicate that cohesin loops are normally removed by late prometaphase. At 30 minutes, *P*(*s*) obtained for CAPH-AID cells (which lack condensin I-mediated loops that would obscure the detection of cohesin loops) is identical to the *P*(*s*) of SMC3-CAPH-AID (which also lack cohesins) at s < 1Mb (Fig. S11A). This loss of cohesin-mediated loops is condensin II-dependent, since 100 kb cohesin loops are readily seen in the *P*(*s*) of prometaphase SMC2-AID cells at 30 minutes (Fig. S11A, black arrow).

The cohesin loops that remain until late prometaphase in the absence of condensins (SMC2-AID cells) are not positioned at CTCF sites, since we do not see an enrichment of CTCF-CTCF interactions (dot score at 30 minutes, Fig.2D, S4C). The likely explanation is that another condensin-independent pathway removes positioning cues for cohesin loops. This background process is likely driven by progressive unloading of CTCF (70). The dominant rapid condensin-mediated removal of cohesin acts in parallel with this background process.

Modeling suggests that condensin removes cohesin loops by either pushing cohesin aside by facilitating unloading of cohesin, which does not rebind. Neither pushing of roadblocks nor facilitated unloading of other SMCs by condensins have been observed in in vitro single-molecule experiments (67). Furthermore, it appears that pushing would require some force, and condensins are relatively weak motors (71, 72), making pushing a less likely scenario. In support of unloading, our ChEP data show that cohesin is lost more rapidly during prophase in the presence of condensin. It thus appears that two processes disassemble interphase cohesin-mediated structures: condensin-mediated unloading of cohesins, and a condensin-independent loss of CTCF.

#### Rule 3. When condensins encounter one another, they stall.

Two lines of evidence argue that condensin II complexes do not bypass each other and instead stall when they encounter each other. First, the average loop size in condensin II-only chromosomes stabilizes and stops growing after 5 minutes in prophase (see below), suggesting that they extrude all available chromatin into loops and then stall (Fig. S4C).

Second, microscopy shows that in condensin II-only chromatids, condensin is concentrated in the interior of the chromatid (Fig. 7C). While models with consecutive loops, i.e. non-bypassing condensins, can reproduce such a localized condensin scaffold distribution (Fig. 7C, S7B), models where loops overlap, i.e. extruding condensins freely bypass other condensins, end up with condensins dispersed evenly throughout the chromatid (Fig. S9F–H).

Our study combines genetics, microscopy, proteomics, Hi-C and polymer modeling to define rules of engagement that dictate the outcome when condensins encounter other condensins and extrusive or cohesive cohesins during mitotic chromosome formation. These rules allow chromosomes to transition from largely cohesin-organized interphase chromatin to condensin-compacted rod-shaped paired sister chromatids. Cohesive cohesin tethering loops between sister chromatids limits the ability of the loop array in each chromatid to adopt a more regular helical folding. We find that mitotic chromosomes are disorderly helices with chromatin loops distributed throughout the body of the chromosome and organized by a discontinuous scaffold.

## Materials and Methods

### Cell culture

#### Cell Culture, transfection to establish stable cell lines, and drug treatments.

Type Chicken DT40 (B-cell lymphoma) cells were cultured in RPMI 1640 medium supplemented with 10% fetal bovine serum and 1% chicken serum in a humidified 39°C incubator with 5% CO_2_ in air. When specified, PIPES-NaOH (0.5 M, pH 7) was added to the culture medium at final concentration 25 μM.

Stable transfection was achieved by two different methods. For random integration of exogenous DNA, a Biorad electroporator was employed following a previously described protocol (73). To insert tags (AID-GFP/Clover, Halo, GFP), the Neon transfection system (Neon1, Thermofisher Scientific) was utilized. Typically, 2–4 million cells suspended in R-buffer (Thermofisher Scientific) were mixed with 2 μg of a rescue plasmid containing a desired tag and a resistance cassette, flanked by ~500 bp homology arms, and 6 μg of a plasmid encoding hCas9 and guide RNA (pX300, addgene 42230). The mixture was then electroporated at setting 24. Approximately 24 h later, serially diluted cells were plated into 6 X 96 well dishes containing the corresponding antibiotics (final concentration 1 mg/ml hygromycin B, 1.5 mg/ml Geneticin).

Indole-3-acetic acid, auxin (57330, Merk, stock 100 mM in ethanol, final conc. 125–150 μM) or auxin derivative 5-Ph-IAA (HY-134653, MedChemExpress, stock 1 mM in DMSO, final conc. 1 μM) was added to the culture to induce rapid degradation of AID-tagged proteins. To depolymerize microtubules, nocodazole (Sigma-aldrich, stock 1mg/ml in DMSO, final conc. 0.5 μg/ml) or colcemid (Thermofisher Scientific, stock 10 μg/ml, final conc. 100 ng/ml) was added to some cultures 30 minutes prior to 1NM-PP1 washout or just after washout, respectively. Custom made 1NM-PP1 (stock 10 mM in DMSO, final conc. 2 μM) was added to cultures to accumulate cells at the G_2_/M boundary. Palbociclib (CDK4/6 inhibitor, stock 1mM in water, final conc. 240 μM) was added to cultures to accumulate cells at the G_1_/S boundary. To prevent mitotic exit, the cell-permeable proteasome inhibitor MG132 was added to WT cultures that were harvested 60 or 90 minutes after release from G_2_ arrest (74)

### Cell lines

All the cell lines used in this study are CDK1^as^ cells. The establishment of DT40 CDK1^as^ cells and expression of OsTIR1 were described previously (6). A list of cell lines, genomic modifications, rescue constructs, and the sequences of guide RNAs used in this study can be found in Tables S6 and S7. Knock-in constructs contain tags such as AID-Clover/GFP, Clover, or Halo, resistance cassettes (hygromycin or geneticin) with loxP sequences, and 2x ~500 bp homology arms. These plasmids were assembled by Dr. Kumiko Samejima (using restriction digests) or by the Edinburgh Genome Foundry (75). Plasmids encoding a guide RNA and hCas9 were assembled by inserting double-stranded oligos into a plasmid pX330-U6-Chimeric_BB-CBh-hSpCas9 which was a gift from Feng Zhang (Addgene plasmid # 42230, (76, 77). Plasmids pMK290 and pAID2.3C were kind gifts from Professor Kanemaki (NIG, Japan). A detailed protocol, all the plasmids (rescue constructs, guide RNAs/Cas9), and cell lines are available upon reasonable request to Dr. Kumiko Samejima. The expression of exogenous proteins and/or homozygous integration of tagged proteins by CRISPR/Cas9 technologies were confirmed by western blot analysis, by genomic DNA sequencing, or by both. Throughout this manuscript, the AID tag refers to the miniAID tag (78). For AID cell line names, “*protein name*-AID” was used for simplicity (excluding GFP/Clover). Unless otherwise specified (e.g. “no auxin”) all cells expressing AID-tagged proteins were treated with auxin or 5Ph-IAA (for wapl-AID cells)(Fig. S13).

### Cell Synchronization and time course experiments

#### CDK1^as^ synchronization and time course experiments

G_2_ synchronization and time-course experiments were performed as described previously (6). In brief, asynchronously growing DT40 CDK1^as^ cells were treated with 1NM-PP1 for 13 hours. As the doubling time of the CDK1^as^ cells is ~10 h, almost all (if not all) cells accumulate at G_2_/M boundary after this treatment. To deplete an AID-tagged protein just prior to mitotic entry, auxin was added to the culture for the final 3 hours of 1NM-PP1 treatment and maintained until harvest. Cells were collected just before 1NM-PP1 washout (G_2_) and typically at 2.5 minutes, 5 minutes, 7.5 minutes, 10 minutes, 15 minutes, and 30 minutes after 1NM-PP1 washout. Cells were fixed in media with 1% formaldehyde (Thermo Fisher Scientific) for 10 minutes at room temperature. Next, 2.5 M glycine was added (final concentration 125 μM) to quench excess formaldehyde for 5 minutes at room temperature and subsequently for 15 minutes on ice. Then, those cells were collected by centrifugation and rinsed with PBS once (or TBS for ChEP analysis). Cell pellets were either frozen on dry ice for Hi-C sample preparation or with liquid nitrogen for protein analysis and kept in −80°C. Characteristics and treatments for each set of cells that served as input for Hi-C can be found in Table S1. For microscopy analysis, small amounts of cells were taken from formaldehyde-fixed cell pellets and stored in methanol/acetic acid (3:1) at −20°C. Those cells were applied on slides, air-dried at room temperature before DNA staining with DAPI in Vectashield Plus antifade mounting medium (H-2000, Vectashield). Images were taken with a wide-field Deltavision Elite microscopy system (Olympus iX71 Inverted Fluorescence Microscope, SoftWoRx software 7.0.0 (Applied Precision Inc, Image Solutions UK Ltd), PCO edge 4.2 sCMOS camera, a X 100 UPLX-Apochromat objective/1.45 Oil). After deconvolution, 3D datasets were visualized and analyzed using Fiji. Representative images of chromatin morphologies (Fig. S1A) and the ratio of cell cycle stages present at each time point of WT CDK1^as^ cells (n=5, >100 cells counted/time point, Fig S1B) confirmed that cells reproducibly entered mitosis in a synchronous manner. Depletion of AID-tagged proteins from auxin-treated cells was confirmed by flow cytometry analysis (GFP/clover protein) and/or mass spectrometry analysis. When AID-tagged protein depletion was not homogeneous in culture, formaldehyde-fixed cells were subjected to a cell sorter (BD FACSAria) to collect GFP/Clover-negative cells with the same level of (background) fluorescence as wild-type cells that do not express GFP/Clover.

#### Double synchronization experiment

SMC3-AID cells were treated with Palbociclib for 14 h to synchronize them at the G_1_/S boundary. Auxin was added for the last 3 h to the cultures of “extrusion only” and “no cohesin (S+G_2_)” conditions to deplete SMC3 throughout S phase. After Palbociclib washout, cells were allowed to proceed through S phase for 7 h in the presence or absence of auxin in the medium supplemented with 25 μM PIPES. Next, 1NM-PP1 was added to the culture for the second synchronization at the G_2_/M boundary. After 3 h incubation with 1NM-PP1, auxin was added to the “no cohesin (G_2_)” culture and auxin was washed out from “extrusive cohesin only” cells. All cells were further treated with 1NM-PP1 for 9 h. Cells were collected at the end of 1NM-PP1 treatment (G_2_) and at 5 min, 15 min, and 30 minutes after 1NM-PP1 washout. Treatments for protein analysis, Hi-C and microscopy were identical to the CDK1-as synchronization protocol (previous section). Additionally, small amounts of cells were released into medium containing colcemid (final conc. 100 ng/ml) and collected at t= 30 min. Those cells were treated with 75 mM KCl for 10 minutes at room temperature (RT) prior to ice-cold methanol/acetic acid (3:1) fixation to prepare conventional chromosome spreads. The expression level of SMC3-AID-clover proteins was detected by flow cytometry analysis. Clover-positive or negative formaldehyde-fixed cells were collected using a cell sorter (BD FACSAria) to remove any dead cells and cells that still expressed detectable levels of SMC3 for no cohesin samples.

#### Measuring the timings of nuclear envelope breakdown

DNA fragments encoding 3x superfolder GFP or mCherry (Addgene plasmids 75385 and 75387 digested with BamHI/XhoI) and double-stranded oligos encoding BP-NLS or NES from Gg cAMP-dependent kinase inhibitor alpha were ligated into pcDNA3 (digested with BamHI/ApaI). These plasmids were randomly integrated into the genome of wild type cells, SMC2-AID cells, and SMC3-AID cells. The expression of GFP or mCherry was confirmed by flow cytometry/microscopy analysis. Wild type cells expressing Halo-laminB1 were described previously (79).

#### Live cell imaging of 3xGFP-NES Halo-Lamin B1 WT cells and its analysis

3xGFP-NES Halo-Lamin B1 WT cells were treated with 1NM-PP1 for ~13 hours in normal media, then transferred to polylysine-coated glass bottom dishes (p35G-1.5–10-C, MatTek) and incubated with SiR-DNA650 (1/1000, Spirochrome) and Halo-JF549 (1/20,000) for ~1 hour. Just prior to image acquisition, the cells were rinsed twice with live cell imaging media (Leibovitz L-15 medium supplemented with 10% FBS and 1% Chicken serum). Images were acquired every 1.5 minutes using Airyscan mode on a Zeiss LSM 880 confocal microscope with a 100x alpha Plan-Apochromat objective/1.4 Oil DIC. Seven sections were taken (1 μm interval). 3D datasets were visualized and analyzed using Fiji. Presented images show a single section of 3D data stacks. The average intensity of GFP signal within a circle drawn at nuclei, cytoplasm, and outside of cells (background) of the single section are measured. The ratio of the intensity (nuclei - background) divided by the intensity (cytoplasm – background) is shown.

#### Fixed cell imaging of GFP or mCherry-NES expressing cell lines and its analysis

3XGFP-NES Halo-lamin B1 WT, 3XmCherry-NES SMC2-AID, 3XmCherry-NES SMC3-AID cells were synchronized with 1NM-PP1 for 10 hours and then further treated with 1NM-PP1 in the presence or absence of auxin for 3 hours. After 1NM-PP1 washout, cells were fixed with a 4% formaldehyde solution at the corresponding time points and DNA was stained with DAPI in Vectashield Plus. Images were acquired at a 0.2 μm interval using a wide-field Deltavision Elite microscopy system and processed as above. Images show a single section of 3D data stacks. The ratio of GFP/mCherry signal intensity was measured and calculated as described above in the previous section. For each of 2 replicate experiments, twenty cells were measured for each time point.

### Light microscopy

#### (Native) chromosome spreads, DNA, Topoisomerase IIalpha and SMC2 staining, fixed cell imaging with Deltavision microscopy, and measurement of Chromosome 1 length and telomere to telomere length

Chromosome spreads were prepared with cells 30 minutes after release from the 1NM-PP1-block at G_2_. To prepare native chromosome spreads, prometaphase cells were rinsed with PBS and then fixed with methanol/acetic acid (3:1) at −20°C. Fixed cells were dropped onto coverslips and air-dried prior to DNA staining with DAPI in Vectashield Plus. For conventional chromosome spreads, cells were treated with nocodazole (0.5 μg/ml) starting from 30 minutes prior to 1NM-PP1 washout. The cells were then collected by centrifugation, suspended in 75 mM KCl, and kept at room temperature for 10 minutes before being fixed with methanol/acetic acid (3:1) at −20°C. Fixed cells were dropped onto coverslips and air-dried prior to DNA staining with DAPI in Vectashield Plus or subjected to indirect immunofluorescence with antibodies against Topo IIα (custom-made antibody in Guinea pig 2B2, (80), 1/1000 dilution) and SMC2 (custom-made antibody made with rabbit 997, (81), 1/500 dilution) or KIF4 (custom-made antibody made with rabbit 1403, (46)1/500 dilution), in TEEN buffer (1mM Triethanolamine:HCl pH 8, 0.2 mM NaEDTA, 25 mM NaCl) for 1 hour. Coverslips were rinsed with KB-buffer (10 mM Tris:HCl pH 7.7, 150 mM NaCl, 0.1% BSA) for 3 × 5 minutes, incubated with anti-guinea pig and anti-rabbit secondary antibodies for 30 minutes, and then rinsed with KB-buffer for 3 × 5 minutes prior to DNA staining with DAPI in Vectashield Plus.

KIF4 stained chromosome spreads were imaged using a Zeiss Elyra 7 lattice SIM microscope with a 63× 1.4NA oil immersion objective. Acquisition settings were: 35 z-planes (91nm interval), G4 lattice (32μm) 13 phases, laser powers: 561nm (500mW) at 4% and 405nm (50mW) at 5%, channel-switching method: frame-fast. Emitted light was captured on PCO edge 4.2M sCMOS cameras using an LP560 emission filter and camera adapter duolink (red light camera 1, blue light camera 2), exposure time 100ms. Processing was by SIM^2^ using “standard fixed” settings of input SNR medium, iterations 20, regularisation 0.025, input and output sampling set to x2, median filter. Grating period was 718.33nm and resultant xy scaling 0.031μm.

DNA Images were acquired at 0.2 μm intervals using a wide-field Deltavision Elite microscopy system, processed as above and analyzed using Fiji. Images with Topoisomerase IIalpha, SMC2 and DNA staining were taken using Airyscan mode on a Zeiss LSM 880 confocal microscope, with a 100x alpha Plan-Apochromat objective/1.4 Oil DIC. Whole cells were imaged with a 0.13 μm interval. After airyscan processing, 3D datasets were visualized and analyzed using Fiji. Images show either a single section of 3D data stacks or max projection as stated in Figure legends.

The length of chromosome 1 was measured by drawing a line along the center of the longest chromosomes within conventional chromosome spreads using Fiji. Telomere-to-telomere length was measured by drawing a straight line between the ends of the longest chromosomes.

#### Imaging of Halo/GFP-tagged protein in formaldehyde-fixed cells with an airyscan microscope, and measurements of sister chromatid widths or immuno stainedwith anti SMC3 antibody

To achieve synchronized mitotic entry, WT and AID-cells were treated with 1NM-PP1 for 10 hours followed by an additional 3 hours with auxin. More than 30 minutes prior to 1NM-PP1 washout Halo dyes were added to the medium in the following dilutions:, Halo-JF646 at 1/1000 dilution (Fig 3A), Halo-JF549 at 1/10,000 dilution (Fig 3C), or Halo-JFX549 at 1/10,000 dilution (Fig 7C, F, I Fig S7B) s. Thirty minutes after 1 NM-PP1 washout, the cells attached to polylysine-coated coverslips were rinsed with pre-warmed PBS and fixed with 4% formaldehyde, except for the SMC3-clover/SMC2-Halo cells in which cells were fixed with 4% formaldehyde in the presence of 0.5% Triton (Fig 3A). DNA was stained with DAPI in Vectashield Plus. Images were taken using Airyscan mode on a Zeiss LSM 880 or 980 confocal microscope, with a 100x alpha Plan-Apochromat objective/1.4 Oil DIC. Whole cells were imaged with a 0.13 μm interval. After airyscan processing, 3D datasets were visualized and analyzed using Fiji. Images show either a single section of 3D data stacks or max projection as stated in Figure legends.

The width of individual sister chromatids or chromosomes (paired sister chromatids) was measured by drawing a straight line perpendicular to the long axis of the sister chromatids on single section images where the intensities of SMC2-Halo were highest. The width of the single sister chromatids in the SMC3-AID cells with auxin or the chromosomes in the SMC3-AID cells without auxin was determined where the intensity of the DNA signal dropped to 50% of the maximum intensity on the line. In case of SMC3-AID cells without auxin, the distance between the SMC3-Halo and the outside edge of sister chromatid (DNA stain) was also measured to estimate the width of sister chromatids. Wapl-aid/SMC2-Halo treated with 5Ph-IAA were fixed as SMC3-clover/SMC2-Halo cells (supple Fig 12B), immunostained with anti-SMC3 antibody (Abcam ab9263 1/500) in PBS plus 3% BSA and visualized with anti-rabbit antibody. DNA was stained with DAPI in Vectashield Plus. Images were acquired at 0.2 μm intervals using a wide-field Deltavision Elite microscopy system, processed as above and analyzed using Fiji.

#### EdU or F-ara-EdU incorporation to label single sister chromatids and measurement of gyre size or staining of condensins

DT40 cells were treated with Palbociclib (final concentration 240 μM) for 11–14 hours. The cells were then rinsed three times with fresh media to wash out Palbociclib, and EdU (final concentration 0.1 μM) or F-ara-EdU (final concentration 10 μM) was added to the medium (final concentration 0.1 μM). After ~11 hours, 1NM-PP1 was added to the medium for 5 hours. Subsequently, auxin was added to the medium for an additional 3 hours to deplete AID-tagged proteins. After washout of 1NM-PP1, cells were further treated with colcemid (0.1 μg/ml) for 30 minutes. The cells collected by centrifugation were subjected to 75 mM KCl for 10 minutes and then fixed with ice-cold methanol/acetic acid (3:1). Cells were then dropped onto coverslips and air-dried. Incorporated EdU or F-ara-EdU was visualized with the Click-it EdU Imaging Kit Plus Alexa Fluor 488 or 555 (Thermo Fisher Scientific) following the manufacturer’s protocol. DNA was stained with Hoechst 33342 and sealed with Prolong Gold Antifade Mountant (Thermo Fisher Scientific). Images were taken using Airyscan mode on a Zeiss LSM 980 confocal microscope, with a 100x alpha Plan-Apochromat objective/1.4 Oil DIC (Z: 0.13 μm interval). After Airyscan processing, 3D datasets were visualized and analyzed using Fiji.

To measure the size of single gyres, EdU incorporated areas which satisfy the following three criteria were selected (see (54)): (1) the EdU signal does not cover the whole width of a sister chromatid, (2) some EdU signal can be seen on the other sister chromatid, (3) and the length of chromosome 1 can be measured within the same chromosome spread (See Fig 7K and (54)). The height of these single gyres was measured by drawing a straight line parallel to the axis of the sister chromatids. The length between the points was measured where the intensity of the EdU signal dropped to 50% of the maximum intensity on the line. The amount of genomic DNA within the single gyre was calculated considering the length of chromosome 1 (196 Mbp) in the same chromosome spread. It was noticed that even though these chromosome spreads were prepared from synchronized cell culture (30 minutes after release from G_2_ block), the measurement of single gyres tends to be more frequent with chromosome spreads which exhibit shorter than average length of chromosome 1. Therefore, a regression analysis between the length of chromosome 1 and the calculated gyre size (Mbp) was performed for each experimental dataset rather than simply calculating the average size. A functional linear regression model and the average length of chromosome 1 (WT: 10.18 μm, SMC3-AID: 7.67 μm, Fig 6D) were applied to obtain the final gyre size in WT and SMC3-AID cells.

### Electron microscopy (EM)

#### EM sample preparation, SBF-SEM imaging, and data acquisition

DT40 cells were attached to a MatTek dish coated with poly-L-lysine and fixed with 2.5% EM grade Glutaraldehyde in 0.1 M sodium cacodylate buffer for 1 hour (pH 7.4). They were then blocked for 15 minutes using a blocking buffer (10 mM glycine, 10 mM potassium cyanide in 0.1 M sodium cacodylate buffer). Cellular DNA was stained with DRAQ5 (10 μM) in 0.1 M cacodylate buffer for 30 minutes, followed by bathing the cells in 2.5 mM diaminobenzidine tetrahydrochloride (Sigma) in 0.1 M sodium cacodylate buffer.

To identify the cells and proceed with photooxidation, the fixed cells were placed on a wide-field DeltaVision Elite (Applied Precision) microscope with a PCO edge 4.2 sCMOS camera and a 100× NA 1.45 UPLX Apochromat objective with oil immersion (refractive index = 1.514) and a Cy5 filter set (640 ± 30) using SoftWoRx 3.6 (Applied Precision) software. The cells were photooxidized by continuous epi-fluorescence illumination for 2 minutes.

Next, the cells were rinsed 5 times for 2 minutes each with 0.1 M sodium cacodylate buffer and then stained for 1 hour with 2% osmium tetroxide and 1.5% potassium ferrocyanide. Subsequently, 1% tannic acid was added to the cells as a mordant for 20 minutes at room temperature, followed by the second osmification step adding 2% osmium tetroxide for 1 hour. Finally, Walton’s lead aspartate (0.02 M lead nitrate in 0.03 M L-aspartic acid, adjusted to pH 5.5) was added. The cells were rinsed 5 times for 2 minutes each with ddH2O between each step. The cells were dehydrated in a graded ethanol series of 30%, 50%, 70%, and 90% in ddH2O for 5 minutes each, followed by 2 rounds of 5 minutes in 100% ethanol, infiltrated with Agar 100 Hard Premix resin (Agar Scientific) at a 1:1 ratio (resin:100% ethanol), and then resin-only for 30 minutes. The cells were embedded in 2 mm of 100% fresh resin and cured for 48 hours at 60°C.

To prepare the blocks, the resin was removed from the MatTek dish using pliers. The photo-oxidized cells and the coordinates on the coverslip were identified using a stereoscopic microscope, and the block was cut with a junior hacksaw and glued to a cryopin (cell side up) using silver epoxy resin. Then, the block was trimmed using a microtome to select the region of interest. The samples were covered with 10 nm gold/platinum before SBF-SEM imaging.

SBF-SEM images were acquired using the Gatan 3View serial block-face system (Gatan, Pleasanton, CA) installed on a FEI Quanta 250 FEG scanning electron microscope (FEI Company, Hillsboro, OR). Images were collected with the following parameters: a pixel size of 0.004 X 0.004 X 0.06 nm, a dwell time of 4 seconds, magnification 5,400, with a chamber pressure of 70 Pa at 3.2 kV.

#### 3D Reconstruction, modeling and segmentation of SBF-SEM images

The electron microscopy images were reconstructed and annotated using AMIRA software (FEI). The selection of the chromosomes was done by setting up a threshold to differentiate between the cytoplasm and the chromosomes. Then, interactive thresholding masks and the Magic Wand tool were used in the segmentation environment to select the chromosomes. The chromosomes were separated using the “separate objects” tool with a 3D interpretation and a neighborhood criterion of 26 connected elements. Chromosomes were identified based on size, length, and centromere position; only chromosome 1 to 5 and chromosome Z could be identified. The Label Analysis module was used to measure geometrical values such as the volume of all identified chromosomes. Surface renders were generated using unconstrained smoothing at level 5. Chromosome length and width were calculated using two methods: 1) using the skeleton tool to draw a line along the chromosomes and measure the length, and 2) using the measuring tool to draw 10 lines along and across the chromosomes.

To stain condenins, cells on coverslips treated with Click-it EdU Imaging Kit Plus Alexa Fluor 555 (Thermo Fisher Scientific) were subjected to indirect immunofluorescence with antibodies against SMC2 (custom-made antibody made with rabbit 997, (82), 1/500 dilution) or CAP-D3 (custom-made antibody PMID 22344259, 1/500 dilution) in TEEN buffer (1mM Triethanolamine:HCl pH 8, 0.2 mM NaEDTA, 25 mM NaCl) for 1 hour. Coverslips were rinsed with KB-buffer (10 mM Tris:HCl pH 7.7, 150 mM NaCl, 0.1% BSA) for 3 × 5 minutes, incubated with anti-rabbit secondary antibodies for 30 minutes, and then rinsed with KB-buffer for 3 × 5 minutes prior to DNA staining with DAPI in Vectashield Plus. Images were acquired at 0.2 μm intervals using a wide-field Deltavision Elite microscopy system, processed as above and analyzed using Fiji.

### Proteomics

#### Chromatin Enrichment for Proteomics (ChEP) and protein quantification based on iBAQ number provided by mass spectrometry

Cells were fixed with 1% formaldehyde for 10 minutes. To inactivate the formaldehyde, 1/20 volume of 2.5 M glycine was added and incubated for 5 minutes before harvesting cells. The fixed cells were washed with TBS (50 mM Tris pH 7.5, 150 mM NaCl), and snap-frozen in liquid nitrogen for storage at −80°C. Once thawed on ice, cells were processed according to the ChEP protocol (79, 82, 83). In brief, formaldehyde-crosslinked cells were lysed in lysis buffer (25 mM Tris pH 7.5, 0.1% Triton X-100, 85 mM KCl). Chromatin was extracted with SDS buffer (50 mM Tris pH 7.5, 10 mM EDTA, 4% SDS), and was washed twice under denaturing conditions (6 M Urea and 1% SDS), followed by a wash with SDS buffer. The DNA content of the chromatin fractions was measured using a Qubit with HS DNA QuantIT (Thermo Fisher Scientific) according to the manufacturer’s instructions.

ChEP chromatin was processed for mass spectrometry by in-gel trypsin digestion. A detailed procedure is described in (39). Following digestion, samples were acidified (pH<3) and spun onto StageTips as described (84). Peptides were then eluted in 40 μL of 80% acetonitrile in 0.1% TFA and concentrated down to 1 μL by vacuum centrifugation (Concentrator 5301, Eppendorf, UK). The peptide samples were then prepared for LC-MS/MS analysis by diluting each one to 5 μL by 0.1% TFA.

LC-MS analyses were performed on a Q Exactive and on an Orbitrap Exploris^™^ 480 Mass Spectrometer (both from Thermo Fisher Scientific, UK) both coupled on-line to Ultimate 3000 HPLCs (Dionex, Thermo Fisher Scientific, UK). Peptides were separated on a 50 cm (2 μm particle size) EASY-Spray column (Thermo Scientific, UK), which was assembled on an EASY-Spray source (Thermo Scientific, UK) and operated constantly at 50oC. Mobile phase A consisted of 0.1% formic acid in LC-MS grade water and mobile phase B consisted of 80% acetonitrile and 0.1% formic acid. Peptides were loaded onto the column at a flow rate of 0.3 μL min-1 and eluted at a flow rate of 0.25 μL min-1 according to the following gradient: 2 to 40% mobile phase B in 180 min and then to 95% in 11 min. Mobile phase B was retained at 95% for 5 minutes and returned back to 2% a minute after until the end of the run (220 min).

For the samples on the Q Exactive, Data Dependent Acquisition (DDA) was used, and the instrument parameters were the same as described (79). On Orbitrap Exploris^™^ 480, for DDA, survey scans were performed at 120,000 resolution with scan range of 350–1500 m/z, normalized AGC target of 3.0E106 and injection time of 50ms. MS2 scans were performed with an isolation window of 1.4 Thomson, and with orbitrap resolution of 15,000. We used HCD fragmentation with normalized collision energy that was set to 30% (85), normalized AGC target at 8.0E104, and maximum injection time at 60ms. The cycle time was set at 3 seconds and only ions with charges between 2 and 7 were chosen for MS2.

For Data Independent Acquisition (DIA) Survey scans were recorded at 120,000 resolution (scan range 350–1650 m/z) with an ion target of 5.0e6, and injection time of 20 ms. MS2 was performed in the orbitrap at 30,000 resolution with a scan range of 200–2000 m/z, maximum injection time of 55 ms and AGC target of 3.0E6 ions. We used HCD fragmentation with stepped collision energy of 25.5, 27 and 30. We used variable isolation windows throughout the scan range ranging from 10.5 to 50.5 m/z. Shorter isolation windows (10.5–18.5 m/z) were applied from 400–800 m/z and then gradually increased to 50.5 m/z until the end of the scan range. The default charge state was set to 3. Data for both survey and MS/MS scans were acquired in profile mode.

All mass spectrometry raw files are available upon request and will be deposited to the ProteomeXchange Consortium (http://proteomecentral.proteomexchange.org) via the PRIDE partner repository. The raw files were processed by MaxQuant version 2.4.9.0 (86) and peptide searches were conducted against the chicken reference proteome set (UP000000539) of UniProt database (Release 2024_01) with additional sequences from our in-house database of chicken proteins, using the Andromeda search engine (87).

Quantification of key proteins associated with chromatin is based on ChEP SILAC analysis data of WT cells with no auxin (n=3), WT cells with auxin (n=3), SMC2-AID cells plus auxin (n=3), and SMC3-AID plus auxin (n=2–4) cells. Note that the n differs because CAP-H, CAP-H2, CAP-G2 proteins were undetected in some of the SMC3-AID samples. Data were normalized against the values of Histone H4. Log 2 ratio against G_2_ samples of the same cells with no auxin (shown as “con” in Fig S2A) (Fig 1C. Fig S2).

The number of condensin I, II, and cohesin complexes on chromatin (per Mb DNA) shown in Fig 1D was calculated as follows. The average iBAQ number of corresponding subunits (CAP-G, CAP-H, CAP-D2 for condensin I; CAP-G2, CAP-H2, CAP-D3 for condensin II; and SMC1, SMC3, RAD21 for cohesin) and Histone H4 were obtained from WT cells (n=3 no auxin, n=3 with auxin). The number of condensin I, II, and cohesin complexes on chromatin (per Mb DNA) was deduced by assuming 2 Histone H4 proteins are equivalent to 180 bp of DNA.

#### Chromatin Enrichment for Proteomics (ChEP) and protein quantification with halo-tagged spike-in control protein by mass spectrometry

Absolute quantification of SMC3, CAP-H, and CAP-H2 on chromatin was performed with a heavy amino acid labeled Halo tagged Histone H4 proteins as a spike-in control. Halo-H4 fusion protein was expressed in E. coli and purified as described below. The ChEP chromatin with the spike-in was processed by filter-aided sample preparation (FASP) (88) to generate tryptic peptides which were analyzed by a mass spectrometer by data-independent acquisition (DIA). The output spectra were analyzed by DIA-NN (version 1.8.1) (89, 90).

The number of corresponding proteins (SMC3, CAP-H, CAP-H2) on chromatin are calculated as follows. First, total MS1 intensities of peptides originating from each protein (SMC3, CAP-H, CAP-H2, Halo tag, histone H4) are obtained. As MS1 intensities are influenced by the composition of peptides, the MS1 (precursor ions) intensity values are adjusted using the Halo-tag protein as a standard protein. The Histone H4 to Halo tag adjustment value was obtained using recombinant Halo-Histone H4. The SMC3, CAP-H, CAP-H2 to Halo tag adjustment values were obtained using the ChEP data of SMC3-Halo, CAP-H-Halo, Halo-CAP-H2 cells, respectively. Finally, the number of SMC3, CAP-H, CAP-H2 on chromatin (per Mb DNA) was deduced by assuming 2 Histone H4 proteins are equivalent to 180 bp of DNA.

#### Expression and purification His_6_-Halo-HistoneH4 of protein and absolute quantification of Halo-tagged proteins

A plasmid encoding the His_6_-Halo-GgHistoneH4 protein was constructed by inserting a synthesized Histone H4 gene into a pH6HTN His6Halotag T7 vector (Promega), which has a TEV cleavage site after the Halo tag. The plasmid was then transformed into an E. coli mutant strain BL21 (DE3) ΔlysA ΔargA, generously provided by Professor Ronald Hay of the University of Dundee, UK (91). The E. coli cells were cultured in M9 medium supplemented with 0.2% glucose, 0.1% thiamine, 100 μg/mL ampicillin, 1 mM MgSO4, 50 μg/mL 13C6, 15N4-arginine, 50 μg/mL 13C6, 15N2-lysine, and a non-essential amino acid mix. Protein expression was induced by adding IPTG to a final concentration of 0.1 mM and incubating at 37°C for 3–4 hours. The E. coli pellets were then snap-frozen in liquid nitrogen and stored at −80°C. The His_6_-Halo-HistoneH4 protein (shown in Fig S1C) was subsequently purified from the inclusion bodies using a Dounce glass/glass homogenizer as described before (92). Briefly, inclusion bodies were solubilized by soaking in 500 ml of DMSO and resuspension in unfolding buffer containing 7 M Guanidine, 20 mM Tris pH 7.5, and 10 mM DTT. After solubilization, a three-step dialysis against Urea buffer (7 M Urea, 100 mM NaCl, 10 mM Tris pH 8.0, 1 mM EDTA, and 5 mM β-mercaptoethanol) was performed. The sample was then loaded onto a 5 ml HiTrap SP cation exchange column (Cytiva) and eluted using a linear gradient from 100 mM to 1 M NaCl in 7 M Urea, 10 mM Tris pH 8.0, 1 mM EDTA, and 1 mM DTT. The fractions containing the pure histone were pooled, aliquoted, and snap-frozen before storage at −80°C. For mass spectrometry analysis to achieve absolute quantification, 0.16–0.4 μg of purified His6-Halo-HistoneH4 protein was mixed with 30 μg of ChEP sample of SMC3-Halo, CAP-H-Halo, or Halo-CAP-H2 cells.

### Hi-C protocol

Chromosome conformation capture (Hi-C 2.0) was performed as described previously (93). Briefly, ~5×106 crosslinked, frozen cells were thawed on ice and lysed for 15 minutes in ice-cold lysis buffer (10 mM Tris-HCl pH8.0, 10 mM NaCl, 0.2% Igepal CA-630) in the presence of Halt protease inhibitors (Thermo Fisher, 78429). Cells were disrupted with pestle A for 2× 30 strokes, centrifuged at 2500 xg, washed, and resuspended in NEBuffer 3.1 (NEB, B9200S). Chromatin was solubilized in 0.1% SDS at 65°C for 10 minutes, quenched by 1% Triton X-100 (Sigma, 93443), and digested with 400 units of DpnII (NEB, R0543M) overnight at 37°C in a total volume of 475 μl. DpnII was inactivated at 65°C for 20 minutes. Digested overhangs were filled in in the presence of 250 nM biotin-14-dATP (Invitrogen, 19524–016) using the large Klenow fragment of DNA polymerase I, (NEB, M0210) for 4 hours at 23°C. Blunted ends were ligated in situ with 50 units of T4 DNA ligase (Invitrogen, 100004817) for 4 hours at 16°C (1200 μl total volume). Cross-links of ligated chromatin were reversed overnight at 65°C using 2 batches of 50 μl of 10 mg/ml proteinase K (Invitrogen, 25530–031). DNA was isolated with 1:1 phenol:chloroform, followed by desalting with an Amicon Ultra-0.5 Centrifugal Filter (EMD Millipore, UFC500396) and 30 minutes of RNase A incubation (Thermo Scientific, EN0531). Biotin was removed from unligated ends by incubation with 15 units of T4 DNA polymerase in the presence of 25 nM dATP/dGTP in NEBuffer 2.1 at 20°C for 4 hours. DNA was sheared using an E220 evolution sonicator (Covaris) and size selected with Agencourt AMPure^®^ XP (Beckman Coulter) to 150– 350 bps. DNA ends were repaired in a mixture of T4 polynucleotide kinase (25 units; NEB, M0201), T4 DNA polymerase (7.5 units; NEB, M0203L) and the large Klenow fragment of DNA polymerase I (2.5 units; NEB, M0210) at 20°C for 30 minutes. The enzymes were inactivated for 20 minutes at 75°C before A-tailing with dATP using 15 units of exonuclease deficient Klenow (Klenow 3’ → 5’ exo-) (NEB, M0212L) at 37°C for 30 minutes. Biotinylated DNA was bound to 12 μl of streptavidin-coated myOne C1 beads (Life Technologies, 650.01). Illumina paired-end adapters were ligated to bead-bound DNA using T4 DNA ligase for 2 hours at room temperature. To determine the minimal number of PCR cycles for Hi-C library generation, a PCR titration was performed before the production PCR (primers PE1.0 and PE2.0). Primers were separated from the 350–550 base pair library using Ampure size selection prior to 50 bp paired-end sequencing on an Illumina HiSeq4000 sequencer.

### Hi-C data analysis

#### Mapping sequenced Hi-C reads

Hi-C paired-end fastq files (BioProject number PRJNA1091327, GEO GSE262525) were mapped to bGalGal1.mat.broiler.GRCg7b (NCBI RefSeq assembly GCF_016699485.2) using the distiller-nf pipeline (https://github.com/open2c/distiller-nf/), as described previously (94, 95). Briefly, reads were aligned with bwa mem (96), and resulting alignments were parsed into Hi-C pairs with the pairtools package (97). We then removed PCR duplicates and kept pairs of uniquely mapped alignments. Pairs with phred scores ≥ 30 for both sides were aggregated into binned contact matrices in the cooler format (98). Contact matrices were normalized using iterative correction balancing after excluding the first two diagonals (99). Bins with extreme coverage were excluded using the MADmax filter on genomic coverage (48). Multi-cooler files and their mapping statistics were submitted to GEO (GSE262525).

#### Contact frequency (*P*(*s*)) curves from pairs of interactions.

To calculate the functions of contact frequency *P*(*s*) vs genomic separation s from Hi-C pairs at bp-level resolution, we used pairtools. Briefly, we split all genomic distances *s* between 1 kb and 1 Gb into bins of exponentially increasing widths, with 128 bins per 10-fold change in s. For every such separation bin, we identified the number of observed cis-interactions within this range of separations (numerator) and the number of all loci pairs separated by such distances in cis (denominator). We then used the built-in functionality of pairtools to smooth the numerator and denominator separately with a Gaussian kernel in the log10-s space with the width of log10_sigma = 0.03. The contact frequency *P*(*s*) was calculated as the ratio of smoothed numerator and denominator. Finally, for these analyses, we only used pairs of loci located on chromosomal arms that were longer than 100 Mb.

#### Contact frequency (*P*(*s*)) curves for binned data

Separately, we also computed the *P*(*s*) curves from binned and balanced Hi-C maps at all used binning resolutions using cooltools (v0.5.035) (100). The *P*(*s*) curves produced by cooltools were used to normalize Hi-C maps for distance-dependent decay of contact frequency (i.e. to calculate observed-over-expected signal, OE) and enable compartment detection, insulation, and loop analysis.

#### Generating a high-resolution “merged G_2_” dataset

The limited sequencing depth of individual libraries did not allow de-novo loop calling independently for each sample. Therefore, we merged 9 Hi-C libraries from all samples at the G_2_ time point that still contained cohesin (i.e., we excluded all SMC3-AID libraries that were induced with auxin addition):

1. SMC3-AID-G2-S1-R1-spna
2. SMC3-CAPH-AID-G2-S2-R1-spna
3. SMC3-CAPH2-AID-30min-S2-R1-spna
4. SMC3-AID-G2-S4-R1-AN-SPnAa
5. SMC3-AID-G2-S4-R1-NN-SPnaa
6. CAPH-AID-G2-S3-R5-spnA
7. CAPH2-AID-G2-S3-R5-spnA
8. SMC2-AID-G2-S4-R5-spNA
9. WT-G2-S4-R5-spna

We generated “merged G_2_” coolers at 1kb, 5kb, 10kb, and 100 kb resolutions and balanced them using the *merge_coolers* and *balance_cooler* functions of the *cooler* library.

#### Loop analysis

Loops (dots) were called from the merged G_2_ cooler at both 5 kbp and 10 kbp resolution using the modified HICCUPS approach via *cooltools.dots* (https://cooltools.readthedocs.io/en/latest/notebooks/dots.html) with the following settings: max_loci_separation=1Mbp, clustering_radius=30kbp, tile_size=5Mbp, lambda_bin_fdr=0.15, max_nans_tolerated=2. Loop calls from both resolutions were combined into a single file containing all loop locations.

We used KDTrees (*scipy.spatial.cKDTree.query_pairs*) to identify and remove dots within a radius of 30kbp (same as clustering radius using the dot calling). For each subset of duplicates, one instance was arbitrarily selected to serve as the representative of the subset.

To quantify the strength of these dots in other conditions and time points, we then applied *cooltools.pileup* to extract chunks of Hi-C maps around each dot (referred to as Hi-C snippets), at a resolution of 1 kb (flank_bp=50 kb). We then averaged these snippets to create an average Hi-C contact map for each dot, also known as a “pileup”. The dot enrichment was calculated as the mean distance-normalized signal (observed/expected) within a 3×3 pixel window around the pileup’s center.

#### Insulation analysis (TADs)

The following protocol was used to create TAD pileups for each Hi-C library:

1. Insulating boundaries were called at 10 kb resolution on the “merged G_2_” cooler using cooltools.api.insulation.calculate_insulation_score (window= 200 kb). Only galGal7b chromosome arms larger than 10 Mb were considered. Boundaries with strength < 0.1 were filtered out to avoid spurious calls.
2. TADs were defined as regions between adjacent strong boundaries. A list of TADs was generated, and only TADs between 100–700 kb in size were selected for further analysis.

The TADs detected in the “merged G_2_” cooler in steps 1–2 were then visualized for each individual library in steps 3–5:

- For each Hi-C library, cooltools.pileup was used to create a stack of regions (referred to as snippets) extending +/− 1 Mb around the center of each TAD. Choosing a 2 Mb window guaranteed that flanks would be sufficiently large for TADs of all selected sizes.
- The stack of snippets was then iterated through, and for each snip, a region of +/− the size of the associated TAD was extracted. Each extracted region was rescaled to a fixed size (100×100 pixels) using mirnylib.numutils.zoomArray.
- The scaled snippets were averaged to produce the “average TAD pileup”, representing the contact footprint of an “average” TAD for each condition and time point.

#### Compartment analysis

The strength of compartmentalization in the G_2_ libraries was computed in two steps.

First, we extracted the first eigenvector (EV1) of all cis Hi-C contact maps from the “merged G_2_” cooler. The largest eigenvector of the Hi-C matrix was computed using *cooltools.eigs_cis* at 100 kb and oriented to have a positive correlation with the GC content of the galGal7b reference. When computing the eigenvector, only galGal7b chromosome arms larger than 10 Mb were retained. Only “cis” eigenvectors were calculated. Separately, the first 3 eigenvectors were derived from each cooler, but not used in this section’s analyses. From this analysis, EV1 was submitted to GEO (GSE262525).

Second, we generated saddleplots for each Hi-C library, providing 100 kb coolers for individual libraries, their associated *P*(*s*), and the “merged G_2_” eigenvector as input (see (101)). This saddleplot showed interactions between 10 groups of genomic bins, sorted and grouped according to their eigenvector values; bins with eigenvector values below the 2.5th percentile and above the 97.5th percentile were ignored. In these plots, AA interactions are shown in the bottom right corner, BB interactions are in the top left corner, and AB interactions are in the top right corner. Compartmentalization scores were then calculated as the ratio of these corner pixels: (AA+BB)/(AB+BA). All scores were normalized to the G_2_ value for the condition set.

#### Simulations of cohesin displacement by condensins

To model the transition between loosely packed interphase chromatin (formed by cohesin) and the condensed mitotic state (formed by condensin II) during prophase, we combined the polymer models of interphase (1) and mitotic loop extrusion (32). Simulations were performed using the open2c/polychrom library (102), similar to previous studies (103). This library utilizes OpenMM (104) for GPU-accelerated molecular dynamics.

We represented chromatin as a chain of 1 kb monomers (10 nm diameter), with each simulation modeling 20 Mb and incorporating three system copies. Lengths are expressed in monomer units, and energies in kT. The model incorporated the following forces:

- Bead Connectivity: Harmonic bonds (*polychrom.forces.harmonic_bonds* with bondLength=1.0, bondWiggleDistance=0.1) linked monomers into a polymer chain.
- Polymer Stiffness: Angle forces (*polychrom.forces.angle_force* with k=1.5) provided polymer rigidity.
- Excluded Volume: Repulsive forces (*polychrom.forces.polynomial_repulsive* with trunc=1.5, radiusMult=1.05) prevented spatial overlap, simulating real polymer behavior.

Periodic boundary conditions maintained a density of 0.224 monomers per cubic nanometer, and a variable Langevin thermostat (collision rate: 0.01 [1/picosecond], error_tol=0.01) controlled temperature.

Our kinetic polymer simulation consists of two stages. First, we modeled interphase chromatin with active cohesin loop extrusion, optimizing parameters to generate a steady-state ensemble of polymer conformations representing a population of cells. Second, we selected 400 conformations from this interphase ensemble. Each conformation served as the starting point for simulations modeling the initial interphase-to-mitotic transition within a single nucleus. Averaging these 400 simulations approximates the population-level behavior observable in our time-resolved Hi-C data.

#### Polymer model of late interphase/prophase cohesin extrusion

Our prophase simulations with loop extrusion follow the framework established in (49). Simulations are initialized with random walk conformations, and we assess equilibration by determining the convergence of contact probability scaling curves. This convergence occurred around 3.5e8 molecular dynamics steps.

The dynamics of cohesin loop extrusion in late interphase and prophase were modeled via a parallel 1-dimensional non-equilibrium Monte-Carlo simulation with the following rules:

- **1D extrusion dynamics**: the polymer chain is represented as a 1-D lattice. Here, a loop-extruding factor (LEF) was conceived and implemented as a walker with a pair of legs that move probabilistically in opposite directions. The location of the legs represents either end of the loop that the LEF extrudes. LEFs load at randomly chosen lattice sites. They extrude loops for a period of time before unbinding, a process that continues throughout the simulation.
- **Collisions and pausing**: When the legs of two LEFs collide, they stall until one LEF unbinds.
- **CTCF barriers**: CTCF sites act as bidirectional barriers. Upon encountering a CTCF barrier, LEFs have a 90% capture probability. Captured LEFs remained at CTCF barriers until unbinding from chromatin. The CTCF barriers were located at the following positions on each of the 3 lattices:
  [0, 55, 201, 239, 516, 551, 666, 717, 806, 1366, 1714, 1905, 2050, 2214, 2386, 2456, 2586, 2713, 2829, 3088, 3315, 3875, 4196, 4480, 4593, 4845, 4972, 5008, 5131, 5228, 5437, 5593, 5626, 5686, 5786, 5840, 5895, 6198, 6250, 6427, 6602, 6818, 6950, 7632, 7898, 8175, 8328, 8423, 8646, 9028, 9395, 9505, 9571, 9770, 9920, 10182, 10231, 10422, 10684, 10853, 10912, 10991, 11287, 11412, 11570, 11631, 12107, 12203, 12388, 12459, 12729, 13180, 13499, 13554, 13634, 13769, 13930, 13959, 14143, 14241, 14315, 14463, 14497, 14619, 14650, 14800, 15100, 15177, 15321, 15460, 15492, 15520, 15620, 15692, 15747, 15837, 16245, 16415, 16569, 16737, 16844, 17029, 17181, 17189, 17254, 17684, 17725, 17837, 17885, 17961, 18072, 18146, 18261, 18544, 18721, 18950, 19184, 19258, 19414, 19528, 19570, 19610, 19753, 19809, 19999]

LEFs from the 1D simulation were then implemented as additional harmonic bonds within the 3D polymer model. These bonds were updated in the 3D simulation to track with the LEFs positions in the 1D simulation.

The loop extrusion process can be characterized by processivity (λ, the amount of chromatin extruded by LEF over its lifetime in the absence of obstacles), separation (d, average spacing between extruders), and extrusion speed (49). From this previous work, we adopted established optimal values for processivity and separation (λ = 100 kb, d = 300 kb). To determine the extrusion speed, we relied on the previous modeling (*1*, *49*)) and iFRAP data (105). These studies estimated the average cohesin loop size at ~100kb and the residence time at ~10 minutes. Together, this implied an effective cohesin extrusion velocity of 10 kb/min, which we used in our interphase model.

Finally, we found the conversion ratio between the time units in simulations and the real world. Live-cell imaging data (106) showed that a 515 kb chromatin region has a Rouse time of ~120 minutes. Our own polymer simulations without cohesin yielded a Rouse time of 6e6 MD steps. Matching the time scales of observed and simulated polymer diffusion yields the time unit conversion ratio. Together, this allowed us to map the real-world cohesins that extruded 100 kb loops at 10 kb/min to model LEFs with λ= 100 kb and LEF step intervals of 10^4 MD steps.

#### Polymer model of prophase condensin extrusion

We selected 400 conformations from the interphase ensemble. Each served as the starting point for a simulation modeling the initial interphase-to-mitotic transition within a single nucleus. On top of active cohesin LEFs, we introduced extra condensin loop extruders to these conformations, using a method similar to cohesin extruders (1D Monte-Carlo coupled to the 3D polymer). Key differences between condensin and cohesin LEFs:

- **Loading and stable binding**: Condensins loaded synchronously and remain bound throughout the simulation.
- **CTCF Interaction:** Condensins bypassed CTCF barriers. This is a reasonable assumption given experimental evidence that CTCF-bound elements do not block loop extrusion by condensins (107).

Cohesin extrusion persisted during this phase. We explored various potential interaction mechanisms upon collision between condensin and cohesin LEF legs (as described in the main text):

- **Stalling:** Identical to interactions between two condensins or two cohesins (remain stalled until unbinding).
- **Bypass:** condensin LEF continued past the cohesin LEF.
- **Push:** condensin LEF displaced the cohesin LEF.
- **Unload:** condensin LEF removed the cohesin LEF.

Importantly, these interactions occur regardless of whether the cohesin was trapped at a CTCF barrier. For condensin LEFs, we used the following parameters: infinite lifetime, average separation d= 700 kb, speed of extrusion = 120 kb/min (see below, chapter “Estimation of loop extrusion speed”, and Discussion).

#### Simulations of cohesion

Our tethered sister chromatid simulations built upon the previously established methodology (32). Crucially, unlike the prophase scenario, which aimed to recapitulate the dynamic process of cohesin loop removal, our simulations of cohesion were designed to sample a dynamic steady state. As above, simulations were conducted using *open2c/polychrom* with the same parameters, unless specified. We modeled two chains of 1-kb monomers (10 nm diameter), each representing an 18 Mb chromatin fiber. Polymer bonds and lengths remained identical to the prophase simulations. To induce sister chromatid separation, we omitted periodic boundary conditions and confinement, simulating good solvent conditions.

Loop extrusion mechanics were largely consistent with the prophase simulations described above. Key distinctions lie in the two additional SMC factors acting on the polymers:

- Condensin I: we modeled a population of LEFs that represent condensins I. In contrast to Condensin-II, Condensin I LEFs are abundant and highly dynamic. The extrusion parameters were adjusted (λ= 800, d= 50) to reflect this and produce the dense extrusion regime required for mitotic chromosomes (25). As with condensins II, condensin-I leg collisions induced stalling.
- Cohesive cohesin: we implemented cohesive cohesins as two-legged walkers (LEFs) positioned on separate chromosomes. The legs of cohesin LEFs stepped forward or backward in steps with equal rates, resulting in random diffusion along the chromosome. Within the 3D simulation, these manifested as bonds between the polymer chains. Cohesins bound randomly (average 300 kb spacing) at the simulation start and remained bound throughout the simulation. Collisions between cohesins’ legs also induced stalling.

Bypassing and stalling interactions between condensins and cohesive cohesins were implemented in the same way as those in the prophase simulations described above. To ensure steady-state LEF distributions, simulations ran for 7.5e6 MD steps. Both cohesin and condensin steps occurred every 750 MD steps, and conformations were saved every 7500 MD steps.

#### Generation of synthetic fluorescence profiles.

Out of the 7.5e6 MD steps in these simulations, 500 conformations from the second half of the simulation were saved and used to extract a radial density profile of the monomers. Three different categories of monomers were considered:

1. Condensin monomers: monomers associated with a condensin LEF at that step of the simulation.
2. Cohesin monomers: monomers associated with cohesive cohesin at that step of the simulation.
3. Generic monomers: all monomers, regardless of whether they are associated with cohesin or condensin.

The radial profile was constructed in the following manner:

1. For each conformation, the spine of each individual sister chromatid is extracted:
  - The polymer conformation was represented as a graph (using the *networkx* Python package) where monomers were nodes and edges represented the bonds that connected monomers.
  - In addition to the polymer bonds of each chromatid, bonds were added between monomers at which a condensin LEF resides.
  - The shortested path between the first and last monomer of each chromatid is calculated (using *networkx.shortest_path*).
2. Monomers from the spines of each sister chromatid were paired to define sections.
  - The distance of each spine monomer to every monomer on the **other** spine was calculated.
  - Every spine monomer of the first chromatid was paired with the monomer on the other spine that was closest to it in 3D space.
  - The above step was repeated for every spine monomer of the second chromatid.
  - The two lists of pairing were compared, and only pairs present in both lists (i.e. spine monomer pairs that were mutually nearest-neighbor) were considered.
  - To mimic perpendicular sections of the sister chromatid from experimental fluorescence, a line was drawn through these pairs.

1. For each monomer category (generic, condensin & cohesin), monomer positions were assigned to sections and collapsed onto the lines.
  - For each line section, the midpoint of the line was assigned as the origin, and all positions were recalculated around that origin.
  - The projection and rejection of each position vector onto the section line were calculated. The projection referred to the footprint (dot product) between the position vector and the section line vector, and the rejection represented the perpendicular distance of that monomer from the line.
  - Monomers were assigned to the nearest line section (i.e. the line section that minimized the rejection), and the associated section projection was chosen for that monomer.
  - Once this was done for every monomer (within a category), a histogram of projection values was computed to represent the density profile obtained from a single conformation.
  - This process was repeated for all 500 conformations, and the counts associated with each histogram were aggregated and visualized (as shown in the Fig. 3B, right panels)

### Polymer models of mitotic chromosomes

Our model was built on four simple principles (Fig. 7A):

- The nucleosome fiber is modeled as a 100 Mb chain of connected 10 nm/200 bp beads that move by random Brownian motion.
- This fiber is folded by condensins into an array of consecutive loops separated by small gaps.
- The resulting bottle-brush of chromatin loops is tightly packed into a cylindrical chromatid body that effectively models condensin-independent condensation of mitotic chromatin (41, 108, 109).
- This chain of loops is weakly “nudged” to follow a helical path by pinning two ends to the opposite caps of the cylinder and fixing the number of turns that it makes around the axis of the cylinder.

The resulting models for chromatids formed by a single condensin complex have three key free parameters (Fig. S9A): (1) average loop size (bp), (2) gap size between condensin complexes (nm), and (3) the linear density of chromatin along the cylinder axis (Mb/μm). Two other parameters (Fig. S9A) were determined from experimental data directly: (1) the length of a turn in Mb (evident from the position of the second diagonal in Hi-C maps), and (2) the volume density of chromatin (measured by SBF-SEM, Fig. 6G). This framework produces a class of individual structural models, whose morphology and contact frequency *P*(*s*) curves are sensitive to changes of each parameter (Fig. S9A). We explored the space of these parameters (see Methods, Fig. S9B), matching the *P*(*s*) curve predicted by each model against that from Hi-C data (Methods).

#### The design of molecular dynamics simulations of chromosomes

In order to provide structural interpretation to our Hi-C data of prometaphase chromosomes in key studied mutants, we built polymer models of chromosomes using molecular dynamics simulations. Overall, our approach was roughly based on those from (5, 6), but included several major novel modifications.

The models and molecular dynamics simulations were implemented in HOOMD-blue v. 4.0.0 (110), a simulation package that provided a rich collection of simulation methods and a flexible and high-performance Python interface.

In all simulations, we modeled 10 nm ‘beads-on-string’ chromatin fiber of nucleosomes as a chain of particles, each representing one nucleosome with 200 bp of DNA. In HOOMD, simulations use a normalized system of units, where particles have a mass of 1.0 [mass] and a diameter of 1.0 [length], where one unit of length corresponds to 10 nm in real coordinates. The particles were connected by harmonic springs (equilibrium length L_bond_ = 1 [length], stiffness = 10 k_B_T/[length]^2. To prevent spatial overlap and model chromatin’s excluded volume, particles interacted via the standard dissipative particle dynamics (DPD) repulsion potential (111):

$${{\vec{F}}_{rep}}^{DPD}=A_{rep}(1-\left|{\vec{r}}_{ij}\right|){\vec{e}}_{ij},r\leq 1.0,\;where\;{\vec{e}}_{ij}={\vec{r}}_{ij}/\left|{\vec{r}}_{ij}\right|$$

We used a repulsion coefficient A_rep_ = 3.0 k_B_T. This prevented overlap while still allowing some strand passing for efficient topological equilibration.

To model the thermal equilibration of chromatin conformation, we subjected the system to the DPD thermostat (111) (target temperature T=1 [temperature], friction coefficient γ=1.0 [mass / time]). Each simulation contained 500,000 particles (representing 100 Mb of chromatin) and ran for at least 1.5e7 steps of dt= 0.05 [time] to ensure thermal equilibration. Three replicates were conducted for each tested set of simulation parameters. Each simulation replicate was executed for 2 days on one NVIDIA GPU (P100, V100, RTX 6000, or A100), provided by the CLIP cluster of Vienna Biocenter. The resulting sets of particle coordinates were analyzed with Python and visualized with Matplotlib (112) and Blender 4.0 Python API (https://www.blender.org).

#### Simulations of SMC3-AID/CAPH-AID chromosomes

To reconstruct the structure of chromosomes formed by condensin II only (SMC3/CAPH-AID cells) we used the model of chromatin fiber described above (which we refer to as model principle (i)) and imposed three additional model principles:

(ii) Formation of loop arrays by Condensin II:

- The chromatin fiber was compacted into an array of consecutive loops separated by small gaps (6, 12). The size of each loop was drawn randomly and independently from an exponential distribution with an average of l_loop_.
- The pair of particles corresponding to the two “anchors” of one loop were connected by a harmonic bond (equilibrium length L_loopbond_ = 1.0 [length], stiffness coefficient = 10 k_B_T/[length]^2).
- Each pair of consecutive loops had their anchors separated by a short “gap” chain segment containing n_gap_ particles. A harmonic force stretched this gap (equilibrium length, L_gap_ = n_gap_+1 [length], stiffness coefficient = 10 k_B_T/[length]^2).
- For each computational replicate, the loop array was generated randomly and independently.

(iii) Condensin-Independent Condensation (108, 113, 114):

- The resulting bottle-brush of chromatin loops was constrained by a cylindrical wall force. We used the built-in HOOMD-blue Gaussian wall potential (epsilon = 1.0 k_B_T, sigma = 0.5 [length]).
- Cylinder radius R_cyl_ and length L_cyl_ were calculated based on the target linear and volume density of chromatin (see below).
- An extra technical challenge arose because HOOMD applied the bounding potential within the specified cylinder, leading to excess volume compaction. To compensate, we slightly increased the input radius and length of the cylinder, ensuring the confining potential reached 0.3 k_B_T at the target radius and length.

(iv) Formation of a helical chromosome:

To model formation of helical mitotic chromosomes, we used a combination of potentials that weakly “nudged” the chain of loops into a helical path:

- We arranged the chromosome linearly along the axis of the confining cylinder by pulling its two ends to the opposite caps of the cylinder with a force of 1.0 [mass * length / time2].
- In the initial conformation, we arranged the particles of loop anchors in a regular helix with a specified number of turns.
- To maintain the number of helical turns of the chromosome throughout simulations, we added a “spool” potential. This potential, a built-in HOOMD Gaussian wall (epsilon=6 k_B_T, sigma=1.0 [length]), was applied radially from a cylindrical “spool” surface (radius = 2.0 [length]), and excluded backbone particles (i.e., the chain formed by loop anchors and “gap” segments between them) from the axial core of the system.
- To prevent the helix from unwinding from the tips, we fixed the angular positions of the chromosome’s two terminal particles (T1 and T2). We achieved this by adding four extra immovable particles: two on opposite ends of the cylinder axis (A1 and A2), and two more (P1 and P2) at the initial positions of T1 and T2. HOOMD torsional potentials were applied to the quadruplets T1-A1-A2-P1 and T2-A1-A2-P2 (setting k=100 kBT), fixing the angular positions of T1 and T2 without restricting radial motion.
- To maintain the consistent size of each helical turn, we additionally restricted the angle of one loop anchor per helical turn using the same technique as above.

The resulting model had 5 geometric parameters (Figure S9A):

1. average loop size, l_loop_ [bp]
2. the size of gaps between anchors of consecutive loops, L_gap_ [nm]
3. the linear density of chromatin along the cylinder axis, ρ_L_ [Mb/μm]
4. the length of a turn in Mb, l_turn_ [Mb]
5. the volume density of chromatin, ρ_V_ [Mb/μm^3^].
  The last parameter was used during the conversion of 3D coordinates into contact frequencies (see below):
6. “contact radius”, σ_C_ [nm]

The five geometric parameters, together with the genomic length of the simulated chromosome, l_chrom_ [Mb], and principles (i)-(iv) were sufficient to fully define the model, allowing the following geometric parameter to be derived.:

1. the length of the confining cylinder [nm], L_cyl_ = l_chrom_ /ρ_L_
2. the radius of the confining cylinder [nm], R_cyl_ = √(l_chrom_/ρ_V_/L_cyl_/π)
3. the pitch of the helix (i.e. the height of one complete helical turn) [nm], P = l_turn_ / ρ_L_
4. the expected number of loops in the chromosome, N_loops_ = l_chrom_ / l_loop_
5. the length of the chromosomal backbone (i.e., the chain formed by loop anchors and “gap” segments) [nm], L_bb_ = N_loop_ * (L_gap_+L_loopbond_)

Two of these parameters, (4) and (5), can be defined from experimental datasets. Previously, we demonstrated that models with helical backbones produce *in silico* Hi-C maps with 2nd diagonals at genomic separations equal the size of the turn, l_turn_ (*6*). Here, we found that this relationship required a minor correction, so to reproduce our 30-minute Hi-C dataset, where the 2nd diagonal is located at 16.4 Mb, we had to set (4) l_turn_ = 17 [Mb]. We relied on the SBF-SEM measurements (see above) to set the volume density of chromatin (5) ρ_V_ = 44 [Mb/μm^3^], which corresponds to the specific volume of (16.5 nm)^3^ per nucleosome or (1.65 [length])^3^ per simulated particle. The rest of the parameters (1)-(3) and (6) were identified via systematic sweeps and comparisons against Hi-C (see below).

#### Calculation of the simulated *P*(*s*) curve

To generate *in silico* Hi-C maps from simulated chromosome conformations, we found neighboring particle pairs in 3D using the Scipy KD-tree algorithm (115). Since KD-trees use strict distance cutoffs, we proposed a novel scheme to calculate more realistic, distance-dependent contact probabilities. To achieve this, we perturb each particle’s position by adding a random shift drawn from a multivariate Gaussian distribution (standard deviation σ_c_ along each axis). Following perturbation, we identify neighboring pairs within a 1.1 [length] distance cutoff. This process is repeated 100 times, and average contact frequencies between all particle pairs are calculated across the replicates. Finally, we calculated *in silico P*(*s*) curves from these contact frequencies, mirroring the procedure used for Hi-C pair analysis.

#### Model selection via comparison of simulated and experimental P(s) curves

To identify simulations best matching experimental Hi-C data on SMC3-AID/CAPH-AID cells at t=30 min, we systematically explored combinations of model parameters (1), (2), (3), and (6). Instead of directly varying linear chromatin density (ρ_L_), we swept over helical pitch (P), which is related to ρ_L_ through P = l_turn_ / ρ_L_.

For each parameter set, we conducted three simulation replicates. We averaged the resulting *P*(*s*) curves and calculated the root mean square (r.m.s.) deviation from the experimental *P*(*s*) in a log10-log10 scale. R.m.s was calculated for genomic distances (*s*) between 3 kb and 40 Mb, with both curves normalized to 1.0 at 3 kb. The top three parameter sets yielded by this procedure had l_loop_=400kb, L_gap_=80–120nm, P=400 nm, σ_C_=48nm.

#### Simulations of SMC3/CAPH2-depleted chromosomes

To model chromosomes formed solely by condensin I (SMC3-AID/CAPH2-AID cells), we modified our approach. Instead of cylindrical confinement (iii), we linearly extended the chromosome within periodic boundary conditions. The chromosome’s ends were fixed in space at a distance L_stretch_ = l_chrom_/ρ_L_. The periodic box length matched L_stretch_, and its width was adjusted to achieve the target volume density (ρ_V_=77Mb/μm^3^, as measured by SBF-SEM).

To reduce the number of simulations and the associated computational costs, we determined the four free parameters (l_loop_, L_gap_, ρ_L_, and σ_c_) in a two-step procedure, leveraging the fact that the shape of *P*(*s*) is independent of ρ_L_ for genomic distances s < 2 Mb (Fig S10A).

Step 1: Defining loop structure. We simulated chromosomes without linear extension, varying l_loop_, L_gap_, and σ_c_. Comparisons to experimental *P*(*s*) data for SMC3-AID/CAPH2-AID cells (3kb - 2Mb separation range) yielded the best estimates for l_loop_ and L_gap_.

Step 2: Defining linear extension. Using the values from Step 1, we then varied ρ_L_ and σ_c_. Matching *P*(*s*) behavior in the 3 kb - 15 Mb range allowed us to determine optimal ρ_L_ and σ_c_ values.

The top three parameter sets yielded by this procedure had l_loop_=100kb, L_gap_=10–40nm, ρ_L_=25 Mb/μm and σ_C_=48–51nm (Fig.S10B–C).

#### Simulations of SMC3-depleted chromosomes

In our models of chromosomes with both condensin I and II (SMC3-AID cells), we built nested loop structures. Our previous work suggested condensin II forms larger, stable loops subdivided by smaller, more dynamic condensin I loops (*6*). This nested structure was directly supported by our new Hi-C data in SMC3-depleted cells (see below).

We implemented a two-step modeling process. First, we generated a layer of loops with average length l_loop_^root^, as described previously. We then subdivided each of these into smaller loops (exponentially distributed with the average l_loop_^nested^). For each of these loop arrays, we specified its own length of gap segments, L_gap_^root^ and L_gap_^nested^.

Finally, since SMC3-AID chromosomes exhibit cylindrical shapes and 2nd Hi-C diagonals, we imposed the cylindrical confinement (iii) and helical backbone (iv) features used in our condensin II model.

This model had 8 parameters: l_loop_^root^, l_loop_^nested^, L_gap_^root^, L_gap_^nested^, ρ_L_ (or, pitch P), l_turn_, ρ_V,_ σ_C_. As previously, we took the target value of volume density from SBF-SEM data (ρ_V_= 77 Mb/μm^3^, Fig.6F–G), and l_turn_=10 Mb to reproduce the 2nd diagonal at 8.4 Mb.

Since a full 6-dimensional parameter sweep is computationally infeasible, we started with a model combining l_loop_^root^ and L_gap_^root^ values from condensin II simulations with l_loop_^nested^ and L_gap_^nested^ from condensin I simulations. Adjusting pitch (P) and σ_C_, we achieved an excellent fit to SMC3-AID Hi-C (t= 30 min) with P= 350 nm or 400 nm and σ_C_= 43 nm (Figure S10D, green curve in S10G).

We then wanted to test if the model based SMC3-AID data could independently support our previous finding of significant gaps between consecutive condensin II loops. Sweeping L_gap_^root^, P, and σ_C_ revealed that 80nm was indeed the global optimum for Hi-C fit (Figure S10D) and that L_gap_^root^ below 40nm do not yield well-matching P(s) curves. However, a second solution emerged with L_gap_^root^=40nm (Figure S10G, yellow curve). This alternative better matched optical microscopy data, predicting a narrower condensin distribution relative to DNA (compare Figure S10E to Figure 7I lower right and Figure S10F to Fig 3C lower right). Therefore, we propose L_gap_^root^=40nm as the solution reconciling both Hi-C and microscopy observations.

#### Simulations of chromosomes with overlapping condensin loops.

Finally, we explored whether our Hi-C and microscopy data on condensin II-only chromosomes (SMC3/CAPH2-AID) supported models where loop-extruding condensin II complexes could completely bypass each other. To model this scenario, we made two minor adjustments to our condensin II simulation approach:

(i) Independent Loop Placement: To reflect complete condensin II-condensin II bypassing, loop arrays were built using N_loop_ independently positioned loops with exponentially distributed lengths (average l_loop_) (for an example, see Figure S9F).

(iv) Enforced Helicity: With no continuous scaffold, helical conformation was maintained by pinning each loop anchor’s angular position using a torsional potential. The target angle for loop i was θ_loop_^i^ = π * (x_left_^i^+x_right_^i^) / l_turn_, (where x_left_^i^ and x_right_^i^ are the genomic positions of the two anchors). Potential stiffness was tuned to k_B_T energy at angular deviation of 1.2 radian.

Through iterative search in a parameter space, we identified one set of parameters yielding a strong Hi-C data match (Figure S9G): l_loop_= 900 kb with one loop per 360 kb of chromatin (in this model, l_chrom_ = 135 Mb, l_turn_= 17.5 Mb).

Our findings suggest that Hi-C data alone may not be sufficient to definitively distinguish between models with and without condensin II bypassing. Importantly, a crucial disagreement arose upon visualizing the 3D conformation of the modeled chromosome. Condensins II did not form a scaffold and appeared distributed across the entire chromosome width (Figure S9H), in stark contrast to microscopy images that clearly showed a distinct scaffold in SMC3-CAPH chromosomes (Figure 7C). This strong contradiction with the microscopy data argued against the possibility of complete loop extrusion bypass by condensin II.

#### Simulations of effects of cohesion on the internal structure of chromatids.

Finally, we aimed to quantify the potential effects of cohesion on the internal structure of individual sister chromatids.

To this end, we modified our models of SMC3-depleted chromosomes (see above):

- We selected a random set of particles at frequency fcoh as initial cohesion sites.
- We assumed that cohesive cohesins can slide along chromatin. We reasoned that in mature chromatids, cohesins will slide to loop tips, thus minimizing the tension imposed by cohesion onto loops. To model this effect, we relocated the initial cohesion sites to their final positions at the midpoint of corresponding loops.
- Finally, we pinned the resulting cohesion sites to the virtual sister chromatid interface on one side of the chromatid via a constant horizontal force of 0.1 kT/nm.

#### Estimation of loop extrusion speed from chromosome morphology dynamics.

Microscopy images revealed that chromosomes achieve their cylindrical shape in around 10 minutes. Concurrently, Hi-C analysis of both SMC3-AID and SMC3-AID/CAPH-AID cells shows that the peak in the *P*(*s*) derivative at the loop size reaches its maximum value at the same time point of 10 minutes. This combined evidence suggests that by 10 minutes, most chromatin has been extruded into loops by condensin II complexes, causing gaps between adjacent condensins to reach their near-minimal value.

We have previously demonstrated how gaps within the extruded loop array can be estimated as g=e^−λ/d^ (12), where g is the fraction of chromatin within gaps; λ is the extruder processivity, i.e. maximally possible loop size per extruder, λ=v_extrusion_ * time; d is average extruder spacing (d=l_chrom_/N_extruders_). Using this relation, we could calculate extrusion speed (v_extrusion_) from the gap fraction (g) and time: v_extrusion_ = - d * ln(g) / time

To estimate the extrusion speed, we could make two further assumptions. (i) Given the stable DNA binding of condensin II in mitosis (38) the size of condensin loops will be limited by their collisions, allowing us to infer d=l_loop_= 400 kb. (ii) The appearance of bottlebrush-shaped chromosomes in microscopy data by 10 minutes requires DNA in each gap to be 100–300% greater than the minimum 80 nm (assuming 1600 bp / 80 nm conversion). This corresponds to a gap fraction (g) of 0.004–0.012. Together, these numbers gave us an estimate of v_extrusion_ = 4–5 * 400kb / 10 min = 2.5–3 kb/sec.

## Supplementary Material

Supplemental Figures and Legends

Video 1

Video 2

Video 3

Video 4

Video 5

Video 6

Video 7

Video 8

Table S1

## Data Availability

Data produced in this paper are part of NCBI BioProject number PRJNA1091327. Hi-C data have been submitted to GEO and are publicly available (accession number: GSE262525). EM data has been submitted to the Electron Microscopy Public Image Archive: EMPIAR-11919 This article is subject to HHMI’s Open Access to Publications policy. HHMI lab heads have previously granted a nonexclusive CC BY 4.0 license to the public and a sublicensable license to HHMI in their research articles. Pursuant to those licenses, the author-accepted manuscript (AAM) of this article can be made freely available under a CC BY 4.0 license immediately upon publication.
