# Supplementary material for: Rules of engagement for condensins and cohesins guide mitotic chromosome formation
Source: Science. Author manuscript; Available in PMC 2025 May 28. (PMC12118822; doi:10.1126/science.adq1709)
Supplement: Supplemental Figures and Legends [file NIHMS2074603-supplement-Supplemental_Figures_and_Legends.pdf]

Science
AAAS

Supplementary Materials for

Rules of engagement for condensins and cohesins guide mitotic chromosome formation

Kumiko Samejima†, Johan H. Gibcus†, Sameer Abraham‡, Fernanda Cisneros-Soberanis‡, Itaru Samejima‡, Alison J. Beckett, Nina Pučeková, Maria Alba Abad, Bethan Medina-Pritchard, James R. Paulson, Linfeng Xie, A. Arockia Jeyaprakash, Ian A. Prior, Leonid A. Mirny*, Job Dekker*, Anton Goloborodko*, William C. Earnshaw*

†,‡ These authors contributed equally to this work

*Correspondence: LAM: Leonid@MIT.edu; JD: Job.Dekker@umassmed.edu; AG: anton.goloborodko@imba.oeaw.ac.at; WCE: bill.earnshaw@ed.ac.uk;

The PDF file includes:

Figs. S1 to S13
Tables S2 to S7

Other Supplementary Materials for this manuscript include the following:

Table S1
Movies S1 to S8

Figure S1

A Mitotic entry of WT CDK1as cells following release from 1NM-PP1 arrest

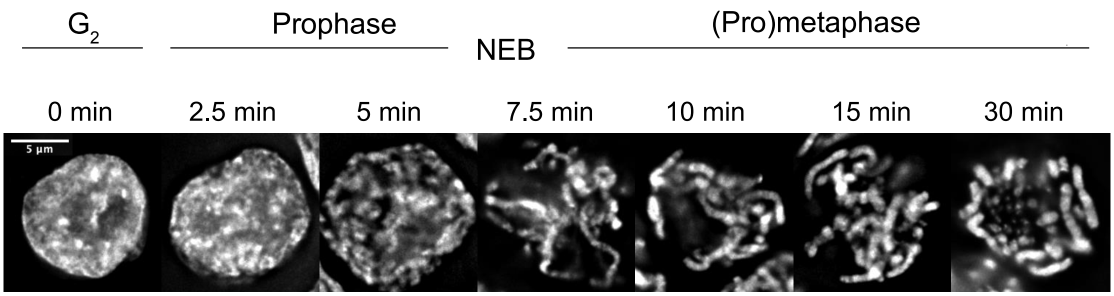

B Relative amounts of individual stages at each timepoint (n=5) in WT

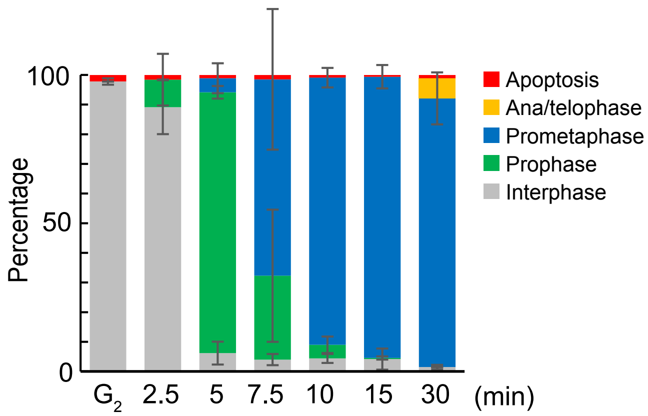

C Halo-H4 (spike-in protein)

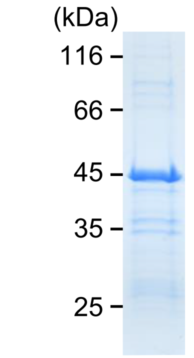

D Timing of mitotic entry (NEB) is similar with or without auxin in WT, SMC2-AID, SMC3-AID cells

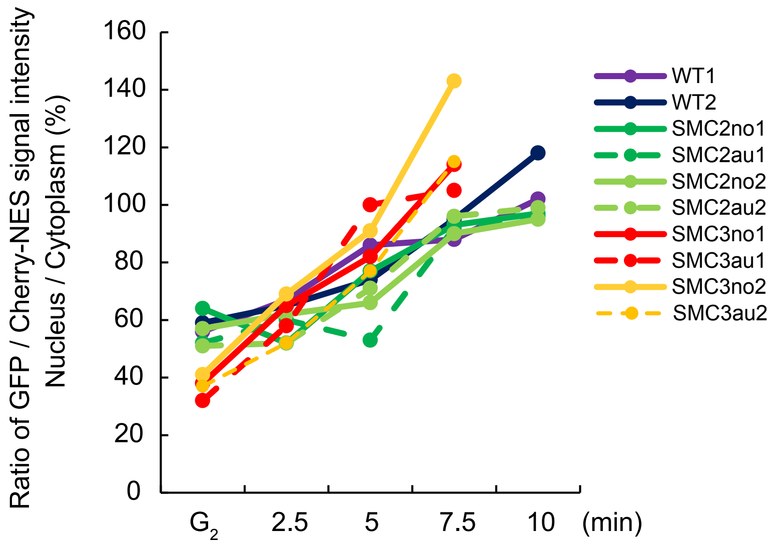

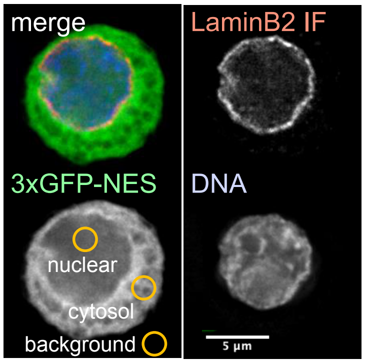

Fig. S1. Mitotic entry of CDK1^{as} cells following release from 1NM-PP1 arrest. (**A**) Representative images of DAPI-stained wild-type (WT) CDK1^{as} cells following release from G_2 arrest. Cells were initially fixed with 1% formaldehyde and then with an ice-cold methanol/acetic acid solution. Scale bar: 5 µm. (**B**) Relative populations of individual mitotic stages at each time point taken from Hi-C samples of WT CDK1^{as} cells. Average and standard deviation (STDEV) are depicted (n = 5). (**C**) Purified Recombinant Halo-tagged Histone H4 protein, labeled with heavy arginine and lysine and used as a spike-in control (**D**) Timing of nuclear envelope breakdown (NEB) in WT, SMC2-AID, and SMC3-AID cells expressing 3xGFP-NES or 3xCherry-NES with or without auxin. Ratio of GFP or Cherry signal average intensity within the nucleus and cytoplasm is shown. A representative image of a G_2 WT cell stained with Lamin B2 antibody, including representative yellow circles to measure the fluorescent intensities, is shown. The average of 20 cells is depicted for each time point with 2 biological replicates. Scale bar: 5 µm.

Figure S2

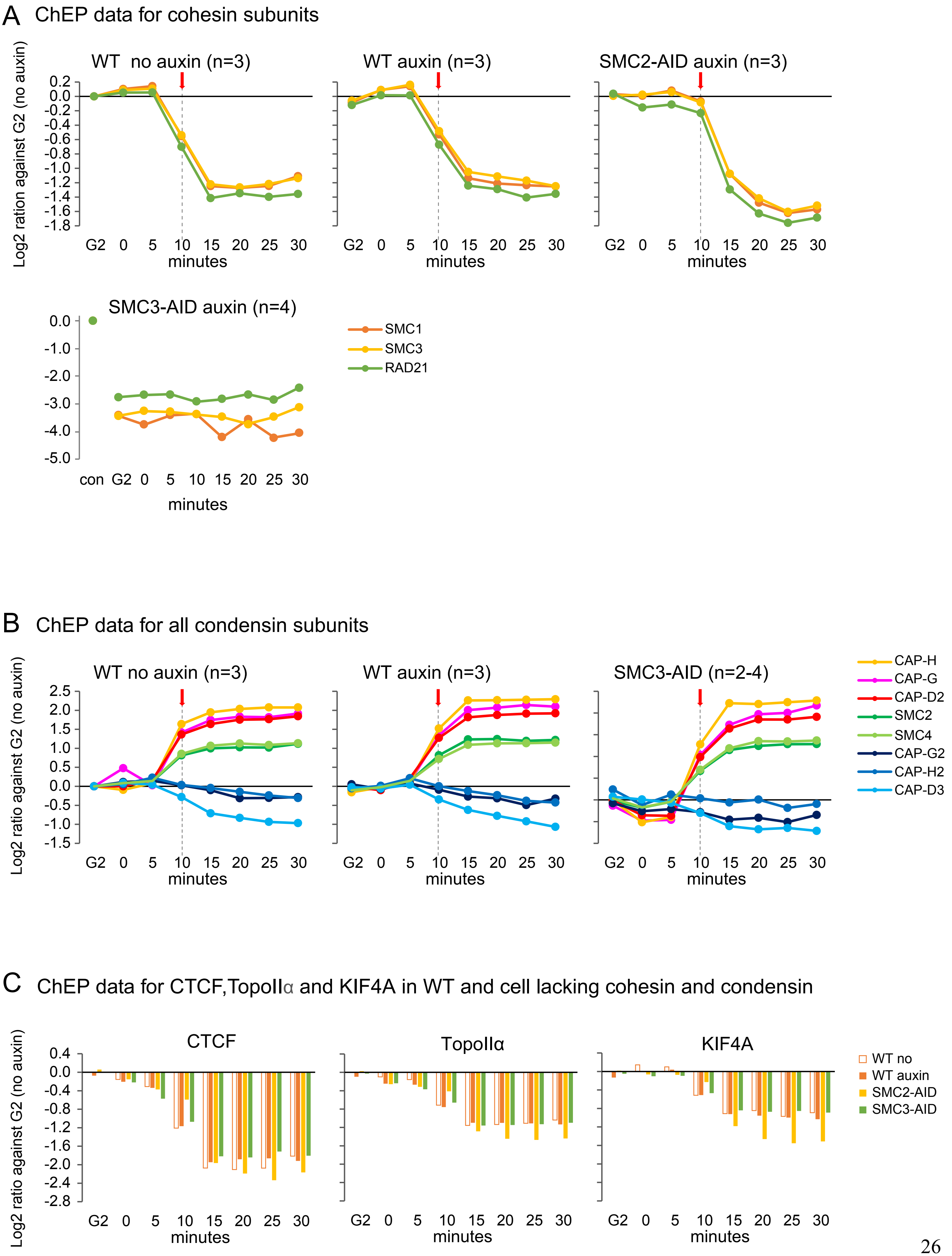
A ChEP data for cohesin subunits
WT no auxin (n=3)
WT auxin (n=3)
SMC2-AID auxin (n=3)
SMC3-AID auxin (n=4)
Log2 ration against G2 (no auxin)
minutes
SMC1
SMC3
RAD21
B ChEP data for all condensin subunits
WT no auxin (n=3)
WT auxin (n=3)
SMC3-AID (n=2-4)
Log2 ratio against G2 (no auxin)
CAP-H
CAP-G
CAP-D2
SMC2
SMC4
CAP-G2
CAP-H2
CAP-D3
C ChEP data for CTCF,TopoIIα and KIF4A in WT and cell lacking cohesin and condensin
CTCF
TopoIIα
KIF4A
WT no
WT auxin
SMC2-AID
SMC3-AID

Fig. S2. Quantification of key proteins associated with chromatin during mitotic entry. (**A**) ChEP data for individual core cohesin subunits (SMC1, SMC3, Rad21) in WT cells no auxin (n=3), WT + auxin (n=3), SMC2-AID + auxin (n=3), and SMC3-AID + auxin cells (n=4). Curves show the Log2 ratio normalized against G_2 without auxin for each cell line. Red arrows: timing of NEB. (**B**) ChEP data for individual condensin subunits in WT no auxin (n=3), WT + auxin (n=3), and SMC3-AID + auxin cells (n=2-4). Con (control) indicates G_2 without auxin. Four experiments were done, but as some condensin II subunits were not detected in some experiments, n=2-4 for SMC3-AID cells. Red arrows: timing of NEB. (**C**) ChEP data for CTCF, Topo IIα, and KIF4A, in WT cells with no auxin (n=3), WT + auxin (n=3), SMC2-AID + auxin (n=3), and SMC3-AID + auxin (n=4).

Figure S3

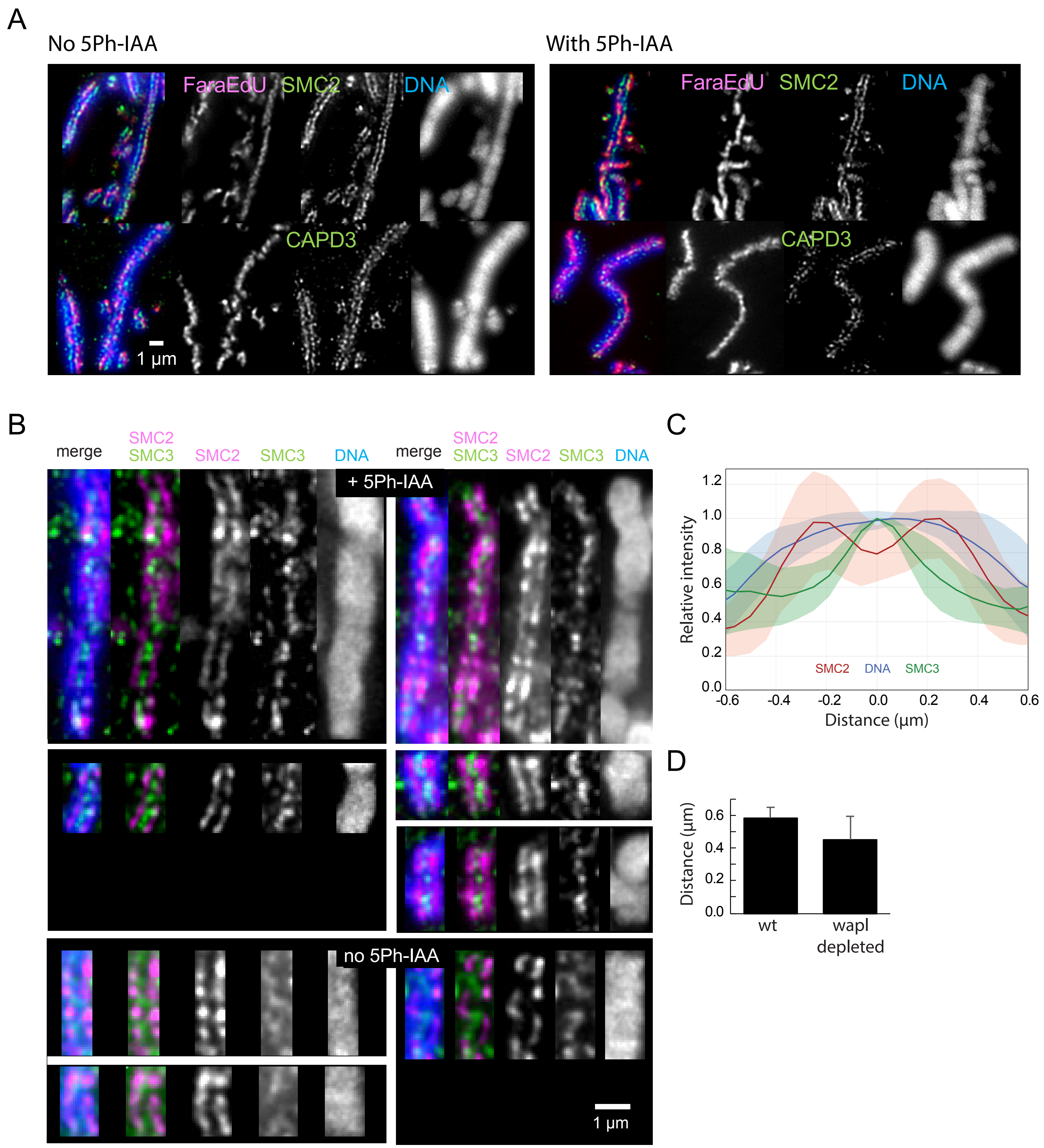
A
No 5Ph-IAA
With 5Ph-IAA
FaraEdU
SMC2
DNA
CAPD3
1 µm
B
merge
SMC2
SMC3
DNA
+ 5Ph-IAA
no 5Ph-IAA
1 µm
C
Relative intensity
Distance (µm)
SMC2
DNA
SMC3
1.2
1.0
0.8
0.6
0.4
0.2
0.0
-0.6
-0.4
-0.2
0.0
0.2
0.4
0.6
D
Distance (µm)
0.6
0.4
0.2
0.0
wt
wapl depleted

Fig. S3. Condensins localize along two separate axes flanking cohesin in sister chromatids of Wapl-depleted prometaphase cells. (**A**) Visualization of separated chromatid axes in chromosome spreads of Wapl-AID cells stained with or without 5Ph-IAA (auxin analog) treatment. Cells were grown for one cell cycle in the presence of F-ara-EdU, and then a second cycle without the drug. In the second mitosis, only one of each pair of sister chromatids is labeled with F-ara-EdU. Conventional chromosome spreads were prepared as described in the legend to Figure S7A. SMC2 or CAP-D3 were stained using specific antibodies. DNA was stained with DAPI. Scale bar 1 μm (**B**) Localization of cohesin (SMC3) and condensin (SMC2) in prometaphase wapl-AID:SMC2-Halo CDK1as cells 30 minutes after release from G2 block with or without 5Ph-IAA (control images). JFX549-Halo (1/10,000) or JFX646 (1/1,000) was added to visualize SMC2-Halo. Cells were fixed with 4% formaldehyde plus 0.5 % Triton to remove chromatin-unbound SMC3. SMC3 was visualized by immunostaining with SMC3 antibody. Maximum projection of ~3 stacked z-sections. Scale bar 1 μm (**C**) Relative fluorescence intensities of SMC3, SMC2-Halo, and DNA from line scans across chromosomes in a single z-section. Plots were centered around the position with the highest SMC3 intensity. Data obtained with 12 chromosomes. Shaded envelopes around the lines represent standard deviations. (**D**) Distance between condensin axis was measured from the data in C and in Fig 3A. average and STDEV is shown

Figure S4

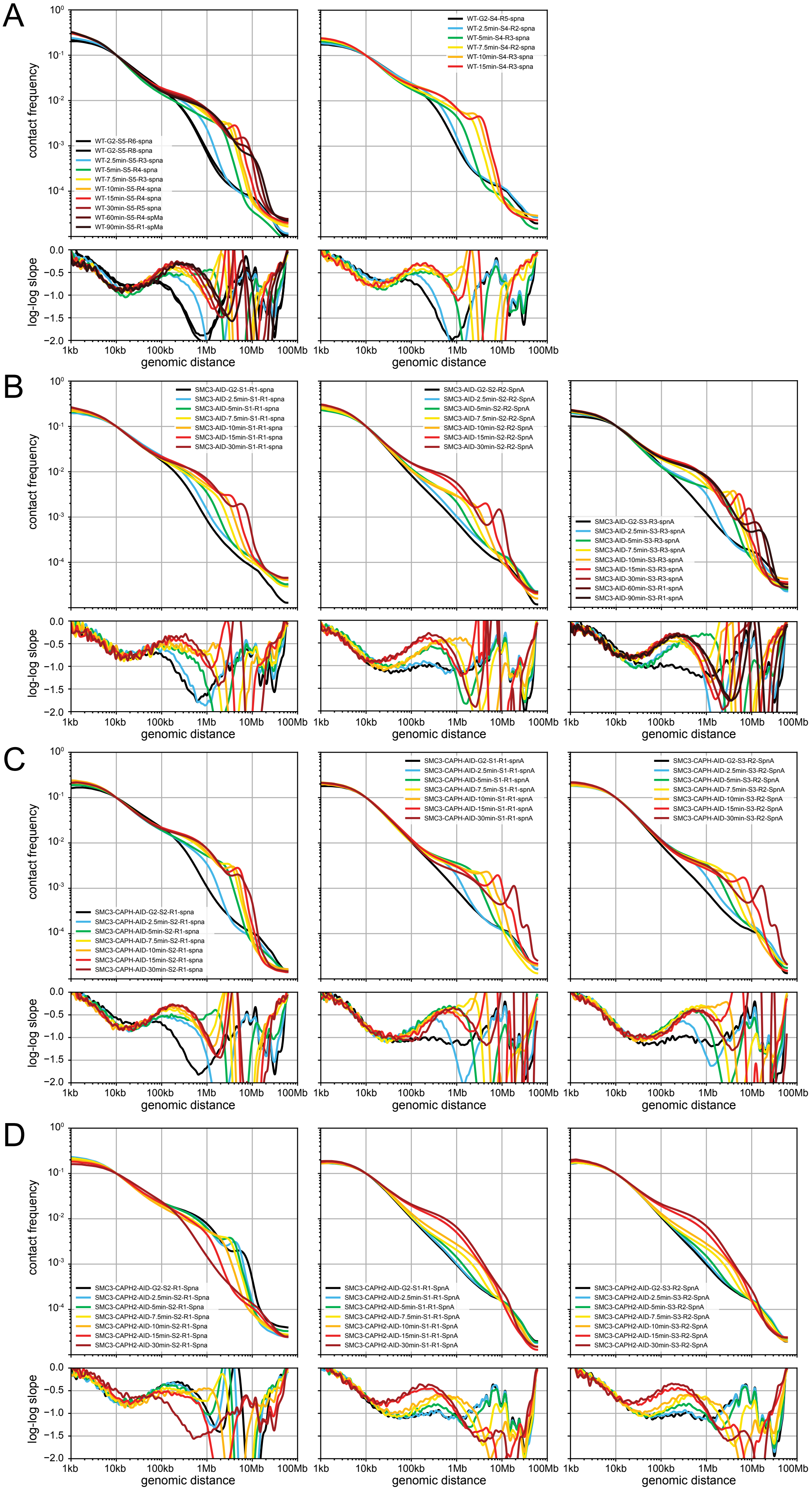
A
B
C
D
contact frequency
log-log slope
genomic distance
1kb
10kb
100kb
1Mb
10Mb
100Mb
WT-G2-S5-R6-spna
WT-G2-S5-R8-spna
WT-2.5min-S5-R3-spna
WT-5min-S5-R4-spna
WT-7.5min-S5-R3-spna
WT-10min-S5-R4-spna
WT-15min-S5-R4-spna
WT-30min-S5-R5-spna
WT-60min-S5-R4-spMa
WT-90min-S5-R1-spMa
WT-G2-S4-R5-spna
WT-2.5min-S4-R2-spna
WT-5min-S4-R3-spna
WT-7.5min-S4-R2-spna
WT-10min-S4-R3-spna
WT-15min-S4-R3-spna
SMC3-AID-G2-S1-R1-spna
SMC3-AID-2.5min-S1-R1-spna
SMC3-AID-5min-S1-R1-spna
SMC3-AID-7.5min-S1-R1-spna
SMC3-AID-10min-S1-R1-spna
SMC3-AID-15min-S1-R1-spna
SMC3-AID-30min-S1-R1-spna
SMC3-AID-G2-S2-R2-SpnA
SMC3-AID-2.5min-S2-R2-SpnA
SMC3-AID-5min-S2-R2-SpnA
SMC3-AID-7.5min-S2-R2-SpnA
SMC3-AID-10min-S2-R2-SpnA
SMC3-AID-15min-S2-R2-SpnA
SMC3-AID-30min-S2-R2-SpnA
SMC3-AID-G2-S3-R3-spnA
SMC3-AID-2.5min-S3-R3-spnA
SMC3-AID-5min-S3-R3-spnA
SMC3-AID-7.5min-S3-R3-spnA
SMC3-AID-10min-S3-R3-spnA
SMC3-AID-15min-S3-R3-spnA
SMC3-AID-30min-S3-R3-spnA
SMC3-AID-60min-S3-R1-spnA
SMC3-AID-90min-S3-R1-spnA
SMC3-CAPH-AID-G2-S2-R1-spna
SMC3-CAPH-AID-2.5min-S2-R1-spna
SMC3-CAPH-AID-5min-S2-R1-spna
SMC3-CAPH-AID-7.5min-S2-R1-spna
SMC3-CAPH-AID-10min-S2-R1-spna
SMC3-CAPH-AID-15min-S2-R1-spna
SMC3-CAPH-AID-30min-S2-R1-spna
SMC3-CAPH-AID-G2-S1-R1-spnA
SMC3-CAPH-AID-2.5min-S1-R1-spnA
SMC3-CAPH-AID-5min-S1-R1-spnA
SMC3-CAPH-AID-7.5min-S1-R1-spnA
SMC3-CAPH-AID-10min-S1-R1-spnA
SMC3-CAPH-AID-15min-S1-R1-spnA
SMC3-CAPH-AID-30min-S1-R1-spnA
SMC3-CAPH-AID-G2-S3-R2-SpnA
SMC3-CAPH-AID-2.5min-S3-R2-SpnA
SMC3-CAPH-AID-5min-S3-R2-SpnA
SMC3-CAPH-AID-7.5min-S3-R2-SpnA
SMC3-CAPH-AID-10min-S3-R2-SpnA
SMC3-CAPH-AID-15min-S3-R2-SpnA
SMC3-CAPH-AID-30min-S3-R2-SpnA
SMC3-CAPH2-AID-G2-S2-R1-Spna
SMC3-CAPH2-AID-2.5min-S2-R1-Spna
SMC3-CAPH2-AID-5min-S2-R1-Spna
SMC3-CAPH2-AID-7.5min-S2-R1-Spna
SMC3-CAPH2-AID-10min-S2-R1-Spna
SMC3-CAPH2-AID-15min-S2-R1-Spna
SMC3-CAPH2-AID-30min-S2-R1-Spna
SMC3-CAPH2-AID-G2-S1-R1-SpnA
SMC3-CAPH2-AID-2.5min-S1-R1-SpnA
SMC3-CAPH2-AID-5min-S1-R1-SpnA
SMC3-CAPH2-AID-7.5min-S1-R1-SpnA
SMC3-CAPH2-AID-10min-S1-R1-SpnA
SMC3-CAPH2-AID-15min-S1-R1-SpnA
SMC3-CAPH2-AID-30min-S1-R1-SpnA
SMC3-CAPH2-AID-G2-S3-R2-SpnA
SMC3-CAPH2-AID-2.5min-S3-R2-SpnA
SMC3-CAPH2-AID-5min-S3-R2-SpnA
SMC3-CAPH2-AID-7.5min-S3-R2-SpnA
SMC3-CAPH2-AID-10min-S3-R2-SpnA
SMC3-CAPH2-AID-15min-S3-R2-SpnA
SMC3-CAPH2-AID-30min-S3-R2-SpnA

Figure S4 (continued)

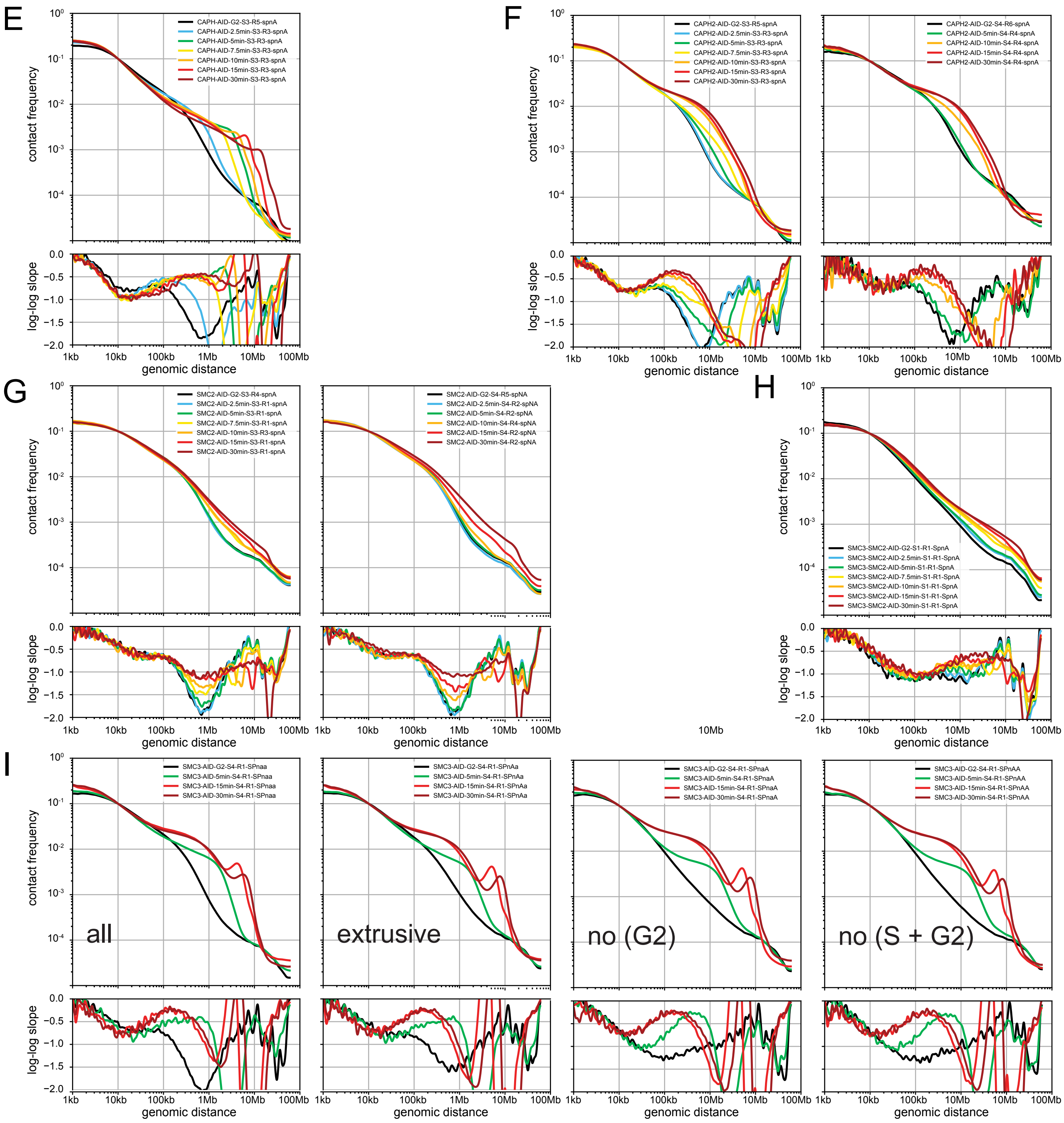

Fig. S4. Plots of contact frequency vs genomic separation, P(s) for all Hi-C libraries used in this study. (**A-I**) The contact frequency P(s) vs genomic separation (s) curves (upper) and their log-log derivatives (lower) for all cell lines.

Figure S5

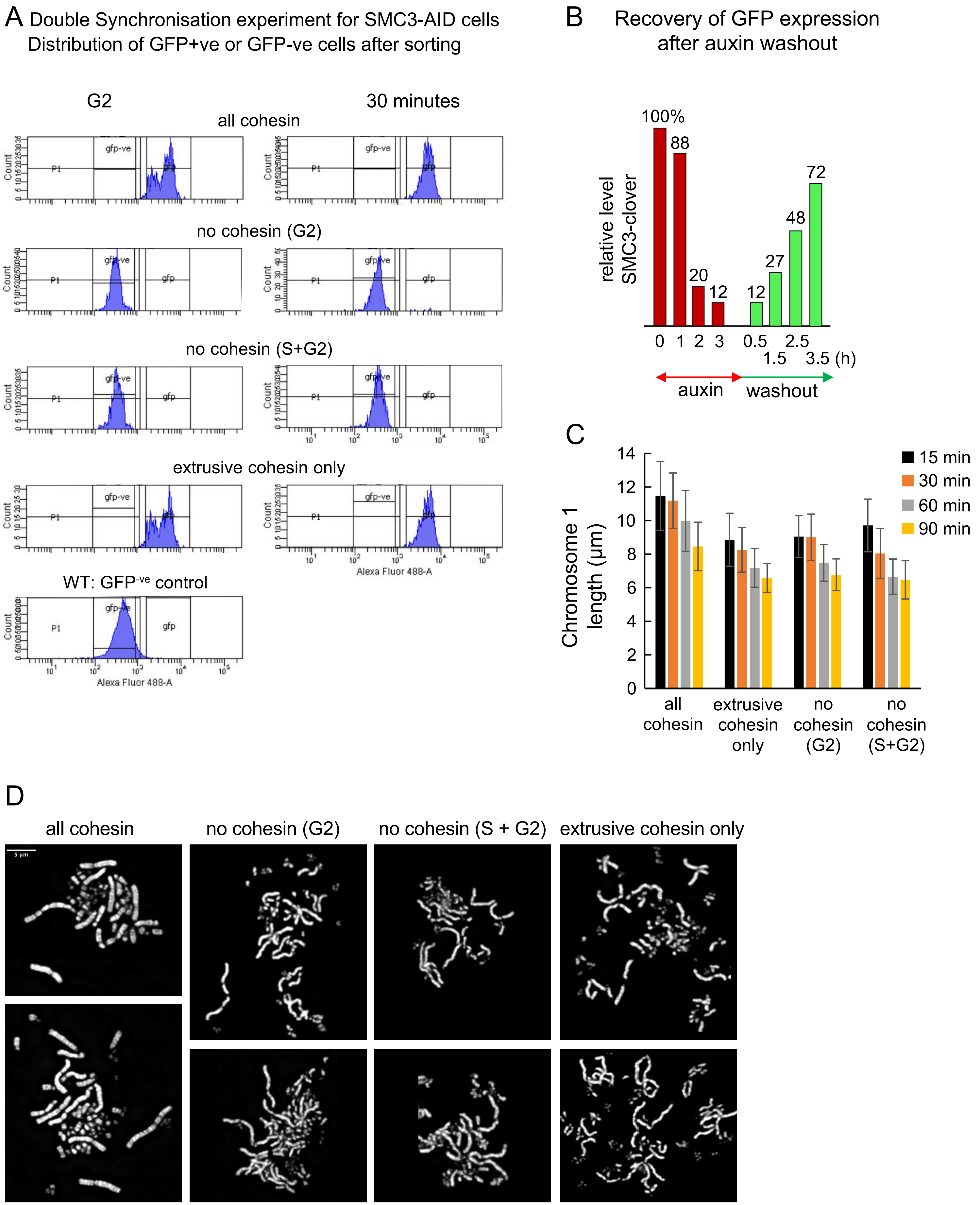
A Double Synchronisation experiment for SMC3-AID cells
Distribution of GFP+ve or GFP-ve cells after sorting
G2
30 minutes
all cohesin
no cohesin (G2)
no cohesin (S+G2)
extrusive cohesin only
WT: GFP-ve control
Count
P1
gfp-ve
gfp
Alexa Fluor 488-A
B Recovery of GFP expression
after auxin washout
relative level
SMC3-clover
100%
88
20
12
12
27
48
72
0 1 2 3 0.5 1.5 2.5 3.5 (h)
auxin
washout
C
Chromosome 1
length (µm)
14
12
10
8
6
4
2
0
15 min
30 min
60 min
90 min
all cohesin
extrusive cohesin only
no cohesin (G2)
no cohesin (S+G2)
D
all cohesin
no cohesin (G2)
no cohesin (S + G2)
extrusive cohesin only
5 µm

Fig. S5. Double synchronization experiment with SMC3-AID cells to obtain populations with only loop-extruding cohesin. (**A**) Level of SMC3-mAID-clover protein at G_2 and 30-minute time points in various samples after cell sorting detected by flow cytometry. These cells were used for Hi-C and microscopy analysis. Control WT cells (bottom left) do not express GFP, and the signal is due to autofluorescence (**B**) Recovery of SMC3-mAID-Clover protein expression after auxin washout in an asynchronous cell culture detected by recovery of GFP fluorescence. The median fluorescence intensity at each time point was normalized against that at time 0. (**C**) Length of Chromosome 1 in chromosome spreads of double-synchronized SMC3-AID cells at 15, 30, 60, 90-minute time points. Measurements represent the average and STDEV from 16 cells at each time point (n=3). (**D**) Conventional chromosome spreads of SMC3-AID cells stained with DAPI. T=30 min. Scale bar: 5 µm.

Figure S6

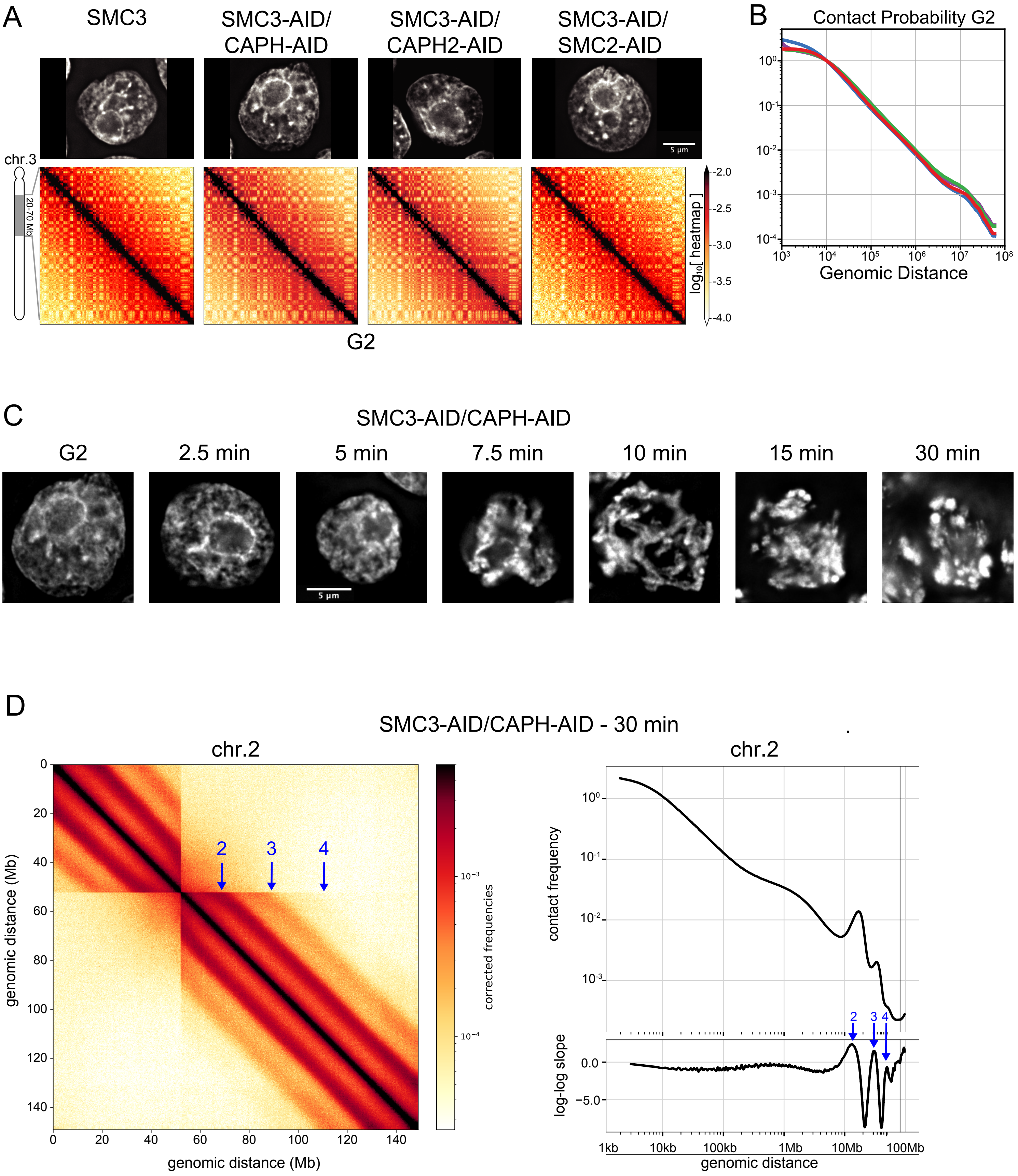
A
SMC3
SMC3-AID/ CAPH-AID
SMC3-AID/ CAPH2-AID
SMC3-AID/ SMC2-AID
5 μm
chr.3
20-70 Mb
log10[heatmap]
-2.0
-2.5
-3.0
-3.5
-4.0
G2
B
Contact Probability G2
Genomic Distance
C
SMC3-AID/CAPH-AID
G2
2.5 min
5 min
7.5 min
10 min
15 min
30 min
5 μm
D
SMC3-AID/CAPH-AID - 30 min
chr.2
genomic distance (Mb)
corrected frequencies
2
3
4
chr.2
contact frequency
log-log slope
0.0
−5.0
1kb
10kb
100kb
1Mb
10Mb
100Mb
genomic distance

Fig. S6. Organization of G_2 nuclei depleted of cohesin and condensin complexes. (**A**) SMC3-AID, SMC3-AID/CAP-H-AID, SMC3-AID/CAP-H2-AID, SMC3-AID/SMC2-AID G_2 cells plus auxin stained with DAPI and Hi-C heat maps of those cells. Scale bar 5 µm. (**B**) Contact frequency P(s) as a function of genomic separation (s) for Hi-C data shown in A. (**C**) Representative images of SMC3-AID/CAP-H-AID cells with auxin stained by DAPI at the indicated times after release from a G_2 arrest. Cells were initially fixed with 1% formaldehyde and then with an ice-cold methanol/acetic acid solution. Scale bar: 5 µm. **(D)** The 3rd and 4th diagonal can be observed on heatmaps for large chromosomes (left) and scaling plots (right). Example shown for chromosome 2.

Figure S7

A Chromosome spread (colcemid, hypotonic, methanol/acetic acid)

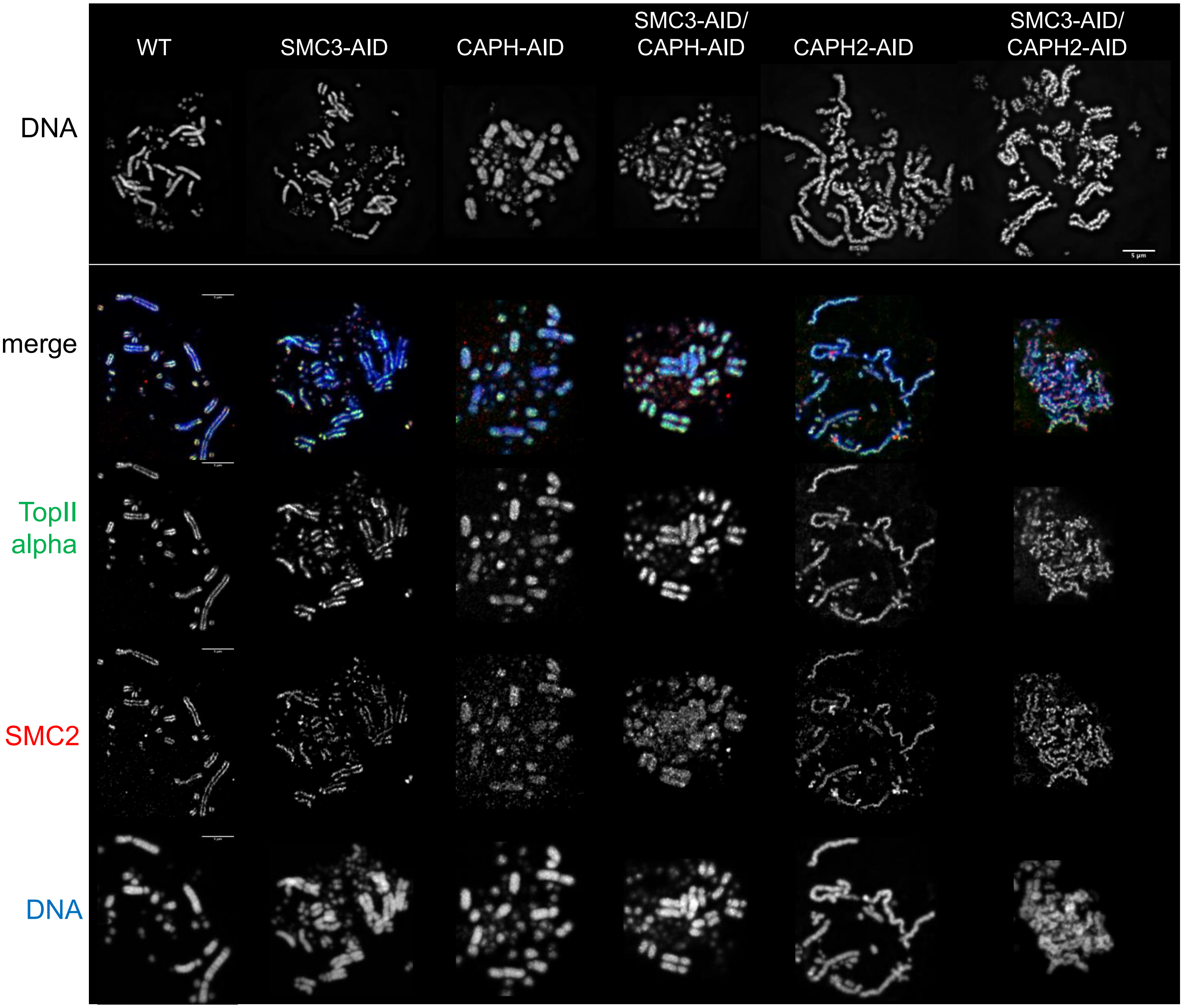

B Chromosome spread (native – no colcemid , no hypotonic, formaldehyde-fixed)

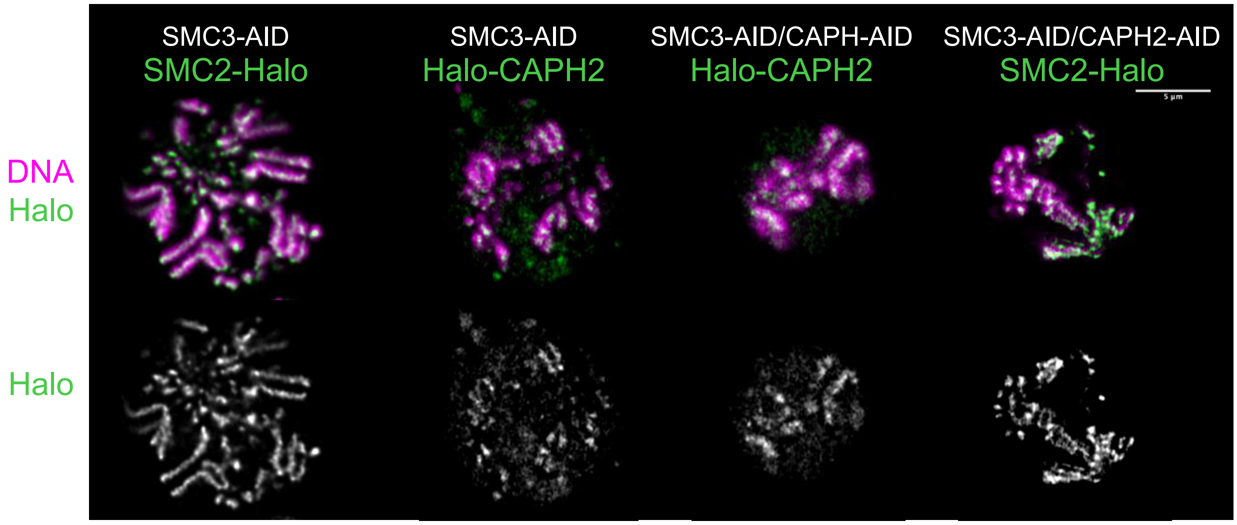

Fig. S7. Axial localisation of chromosome scaffold proteins in WT and various mutant cells varies depending upon sample preparation method. (**A**) Conventional chromosome spreads stained with DAPI and antibodies against Topo IIα and SMC2. Cells were treated with colcemid for 30 minutes after 1NM-PP1 washout (T=30 min), collected and then hypotonically swollen with 75 mM KCl for 10 minutes prior to fixation with cold methanol/acetic acid. A single Z section is shown. Note: Topo IIα and SMC2 localize diffusely throughout the entire chromosome in CAP-H-AID and SMC3-AID/CAP-H-AID cells after auxin treatment. Scale bar 5 µm. (**B**) For "Native" chromosome spreads, cells were rinsed with PBS and then fixed with 4% formaldehyde at T=30 min after release from G_2 arrest. Halo-JFX549 (10,000) was added to the culture >30 minutes prior to 1NM-PP1 washout to label Halo-tagged proteins. A single Z section is shown. Note: In contrast to (A) Halo-CAPH2 is not diffuse throughout the chromosome in SMC3-AID/CAP-H-AID cells, but is confined to a more central region. Scale bar: 5 µm.

Figure S8

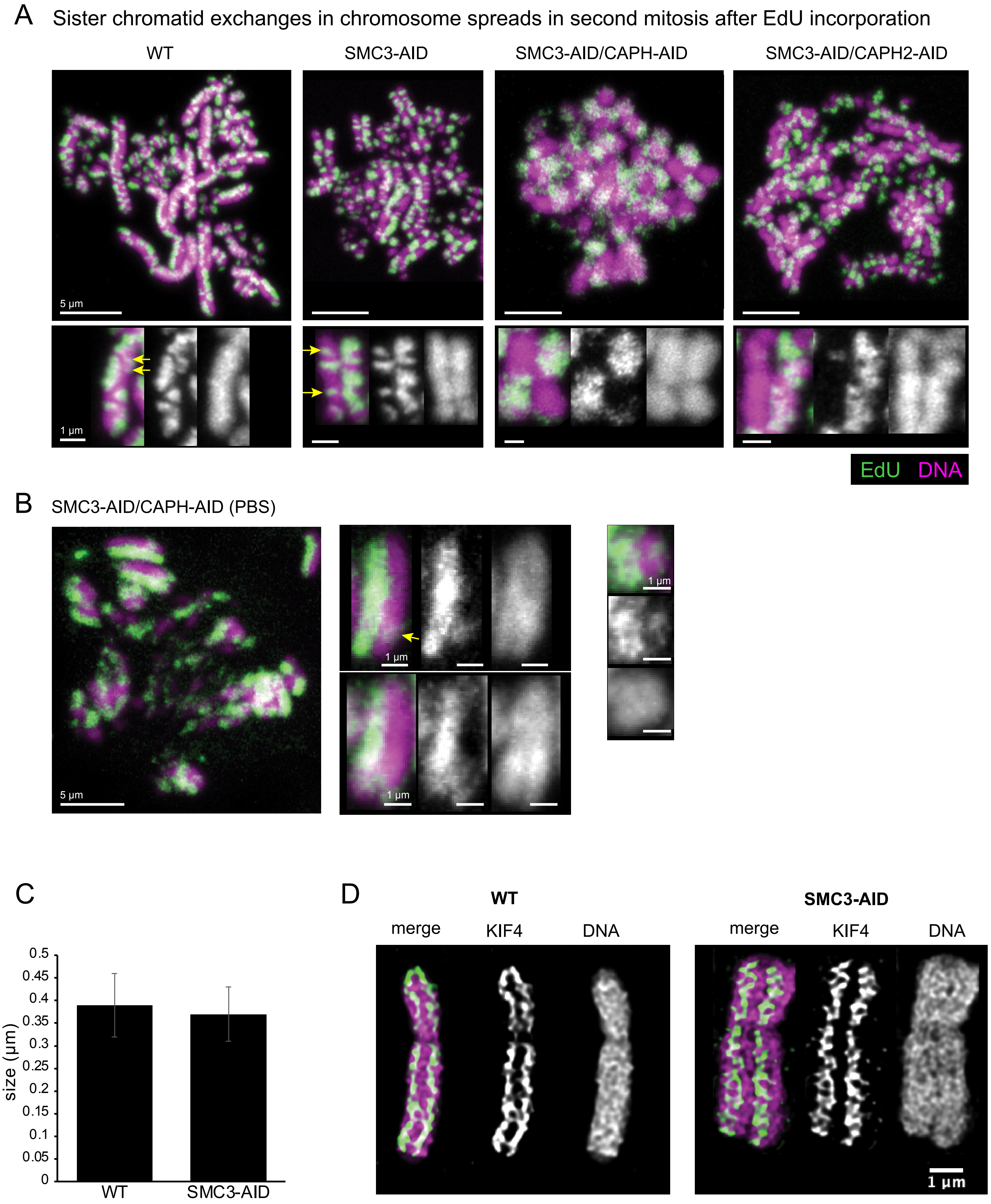
A Sister chromatid exchanges in chromosome spreads in second mitosis after EdU incorporation
WT
SMC3-AID
SMC3-AID/CAPH-AID
SMC3-AID/CAPH2-AID
5 µm
1 µm
EdU DNA
B SMC3-AID/CAPH-AID (PBS)
5 µm
1 µm
1 µm
1 µm
C
size (µm)
0.5
0.45
0.4
0.35
0.3
0.25
0.2
0.15
0.1
0.05
0
WT
SMC3-AID
D
WT
SMC3-AID
merge
KIF4
DNA
merge
KIF4
DNA
1 µm

Fig. S8. Sister chromatid exchanges in chromosome spreads in the second mitosis after EdU incorporation. (**A**) Chromosome spreads of mitotic cells stained with DAPI to visualize DNA and azide-488 to visualize EdU. Cells were grown for one cell cycle in the presence of EdU, and then a second cycle without the drug. In the second mitosis, only one of each pair of sister chromatids is labeled with EdU. Conventional chromosome spreads were prepared as described in the legend to Figure S7A. Scale bar 5 µm (whole cell), 1 µm (enlarged image). Arrow heads point the EdU positive regions which indicate single gyres. (**B**) Similar to A, but "native" chromosome spreads were prepared by fixing cells depleted of SMC3 and CAPH with methanol/acetic acid solution in the absence of colcemid and hypotonic treatment. These chromosomes are longer than those prepared by conventional spreading, so some sister chromatid exchanges can be observed. In these chromosomes, which contain only much larger chromatin condensin II-extruded loops, the sister chromatid exchanges are diffuse and cannot be measured accurately. **(C)** Height of EdU signal representing a single gyre. WT (n=94) SMC3-AID (n=88). Average: 0.39 µm and 0.37 µm. Standard deviation: 0.07 µm and 0.06 µm, respectively. **(D)** Super resolution images of WT and SMC3-depleted (SMC3-AID + auxin) conventional chromosome spreads stained with KIF4 antibody. scale bar 1µm.

Figure S9

Model fitting SMC3-AID/CAPH-AID

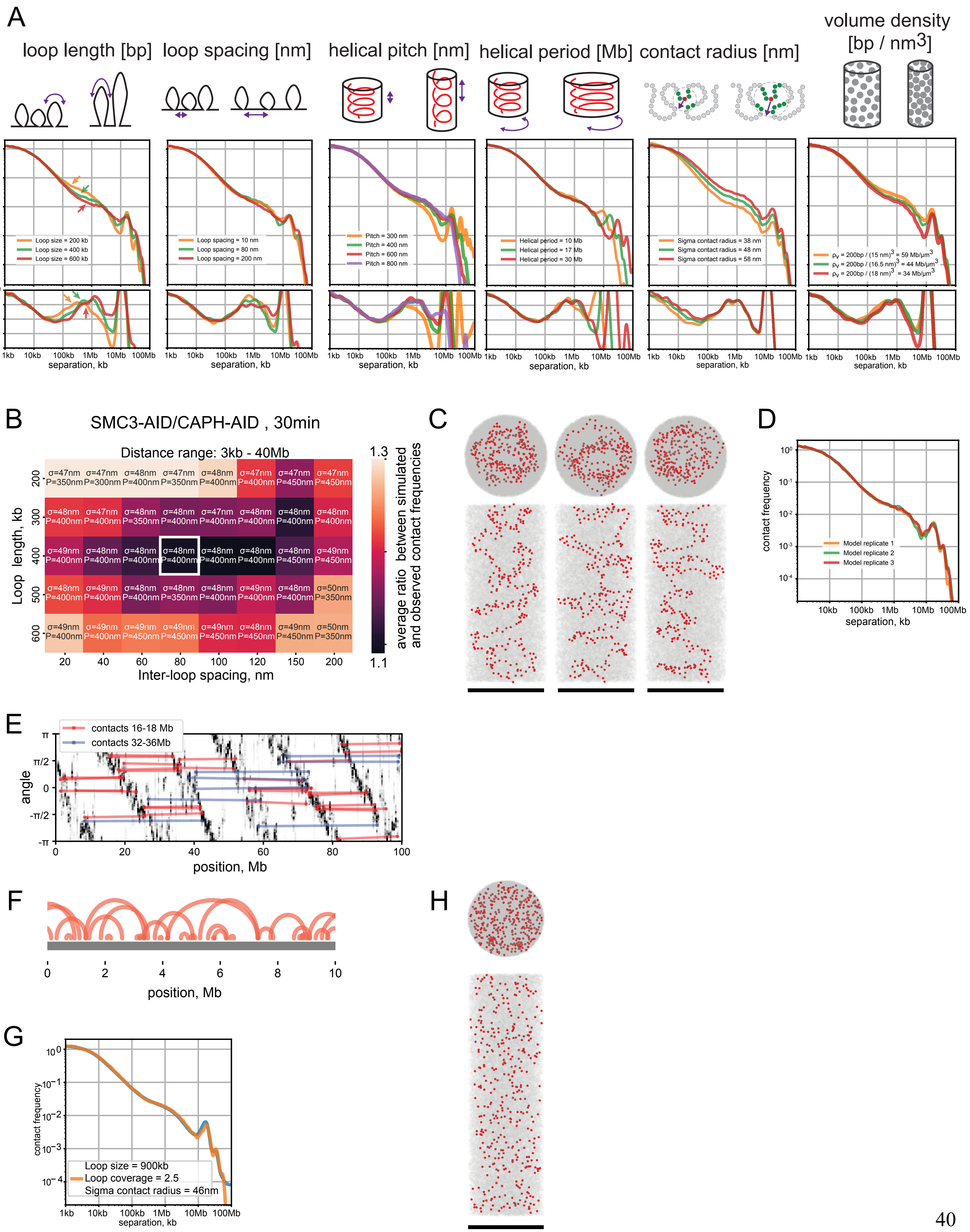

Fig. S9. Fitting a polymer model of condensin II-only (SMC3-AID/CAPH-AID) chromosomes. (**A**) The key six parameters of the model and their effect on the contact frequency P(s) vs genomic separation s curves. Top: the cartoon illustrating the effect of increasing the parameter on the polymer model. Middle: P(s) curves for several values of the parameter. Bottom: the slopes of P(s) curves in log-log space vs genomic separation s. In all models, unless specified, loop size=400kb, inter-loop spacing=80nm, helical pitch=400nm, helical period = 17Mb and sigma contact radius = 48nm. For slope calculation, P(s) was smoothed with sigma_log10 = 0.12. (**B**) The discrepancy between simulated and experimentally measured P(s) curves as a function of four model parameters. The color of each pixel shows the discrepancy of the best-fitting model from a group of models with two parameters (loop length and inter-loop spacing) specified by the x- and y- coordinates of the pixel. The annotation of each pixel shows the best-fitting values of the other two parameters (sigma contact radius and helical pitch) within the group specified by the x- and y- coordinates. The white square shows the parameters of the model selected for figure 7. (**C**) Chromosome conformation in three simulation replicates of the best-fitting model. Chromatin shown in gray, condensins II in red. (**D**) P(s) curves obtained from individual simulation replicates. (**E**) The 2D-histogram of the angular positions of chromatin particles (y-axis) vs their genomic position (x-axis) in the best-fitting model. The diagonal striped pattern originates from the helical organization of the chromosome. The colored lines indicate 30 randomly selected 3D contacts from the 2nd (red) and 3rd (blue) Hi-C diagonals. These lines illustrate that these diagonals are produced by interacting particles from consecutive gyres of the helix. (**F-H**) The best-fitting polymer model with overlapping condensin loops (F) can reproduce Hi-C P(s) (D) but lacks backbone-like staining of condensins (H).

Figure S10

Model fitting SMC3-AID/CAPH2-AID

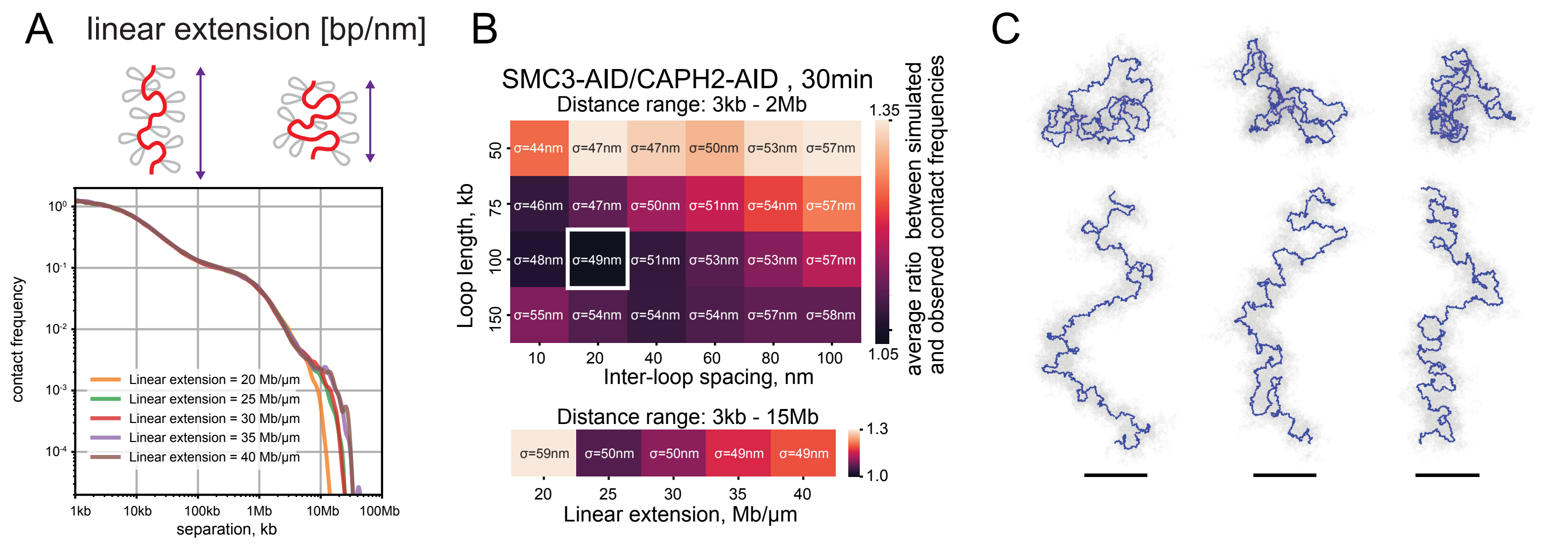

Model fitting SMC3-AID

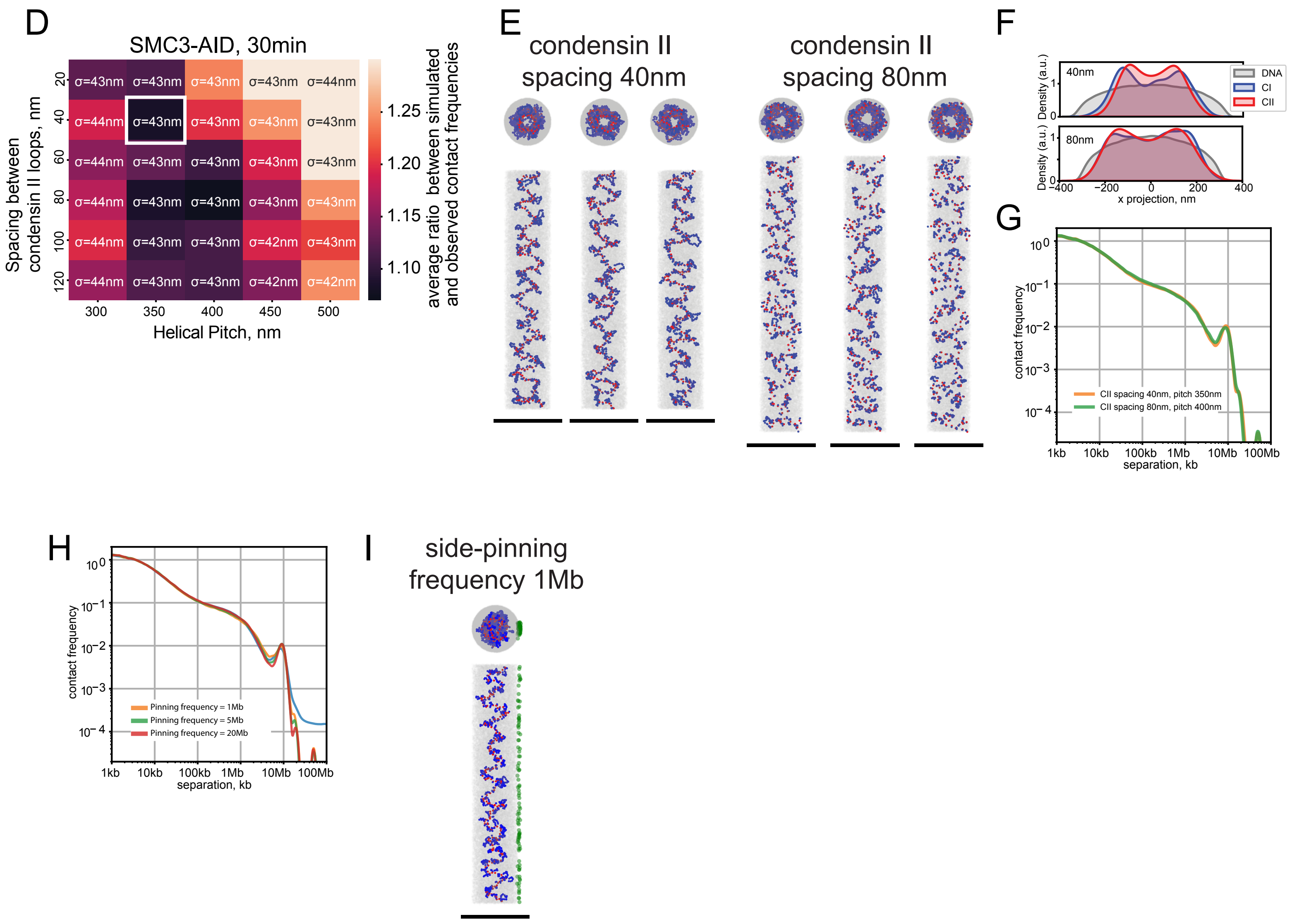

Fig. S10. Fitting polymer models of condensin I-only (SMC3-AID/CAPH-AID) and condensin I+II (SMC3-AID) chromosomes. (A-C) Fitting a model of condensin I-only chromosomes. (**A**) The effect of the linear extension of the chromosome on the contact frequency P(s) vs genomic separation s curve. In these simulations, loop size = 100kb, inter-loop spacing = 20nm, and sigma contact radius = 50nm. (**B**) The discrepancy between simulated and experimentally measured P(s) curves as a function of model parameters. Top: the discrepancy of P(s) in the 3kb-2Mb range in models without linear extension. The color of each pixel shows the discrepancy of the best-fitting model from a group of models with two parameters, loop length and inter-loop spacing, specified by the x- and y- coordinates of the pixel. The annotation of each pixel shows the best-fitting value of sigma contact radius. The white square shows the parameters of the model selected for figure 7. Bottom: the discrepancy of P(s) in the 3kb-15Mb range for models with fixed loop length and inter-loop spacing as a function of linear extension. (**C**) Chromosome conformation in three simulation replicates of the best-fitting model. Chromatin is shown in gray, condensins II in red. (**D-G**) Fitting a model of condensin I+II chromosomes. (D) The discrepancy between simulated and experimentally measured P(s) curves as a function of spacing between condensin II loops, helical pitch, and sigma contact radius. The white square shows the parameters of the model selected for figure 7. (E) Chromosome conformation in three simulation replicates of the best-fitting model with condensin II spacing of 40nm (left) and 80nm (right). (F, G) The cross-sectional distribution of condensins (F) and P(s) (G) in models as a function of condensin II spacing. (H) P(s) in simulations of cohesion via side-pinning of loop tips as a function of side-pinning frequency. (I) Chromosome conformation in cohesion models; green dots show the positions of pinned loop tips.

Figure S11

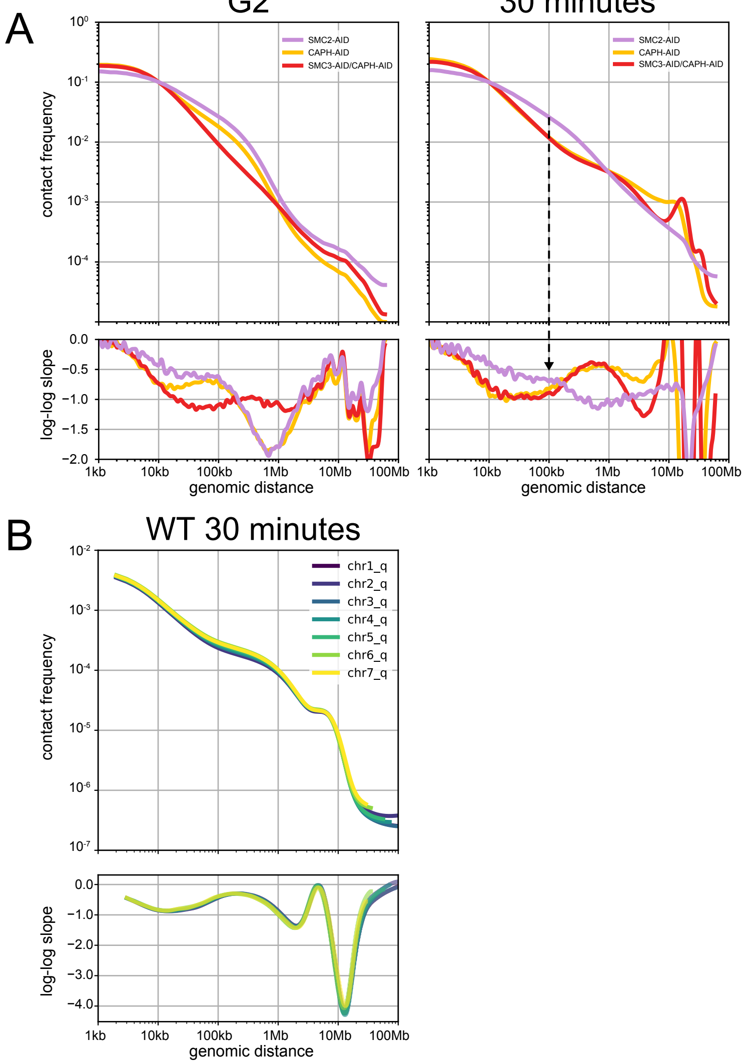

A
G2
30 minutes
SMC2-AID
CAPH-AID
SMC3-AID/CAPH-AID
contact frequency
log-log slope
genomic distance
1kb
10kb
100kb
1Mb
10Mb
100Mb
B
WT 30 minutes
chr1_q
chr2_q
chr3_q
chr4_q
chr5_q
chr6_q
chr7_q

Fig. S11. Condensin II removes cohesin loops. (**A**) The contact frequency *P(s)* vs genomic separation (*s)* curves for SMC2-AID cells (lacking both condensins, but having cohesins), CAPH-AID (having cohesins and condensin I), and SMC3-AID/CAPH-AID (having condensins II only) in G_2 (left) and prometaphase (right). The characteristic bump on the P(s) at ~100kb (black arrow) reveals the presence of cohesin loops in G_2 samples with intact cohesins (SMC2-AID and CAPH-AID), but not in the sample depleted of cohesin (SMC3-AID/CAPH-AID). In prometaphase, cohesin loops persist in the sample lacking both condensins (SMC2-AID), but cohesin loops are gone in the sample with active condensin II (CAPH-AID). (**B**) The contact frequency P(s) vs genomic separation (s) curves for long chromosome arms of the first 7 macro-chromosomes in WT cells (30 minutes in mitosis) shows that the position of the second diagonal and general distance decay does not vary between these macro-chromosomes.

Figure S12

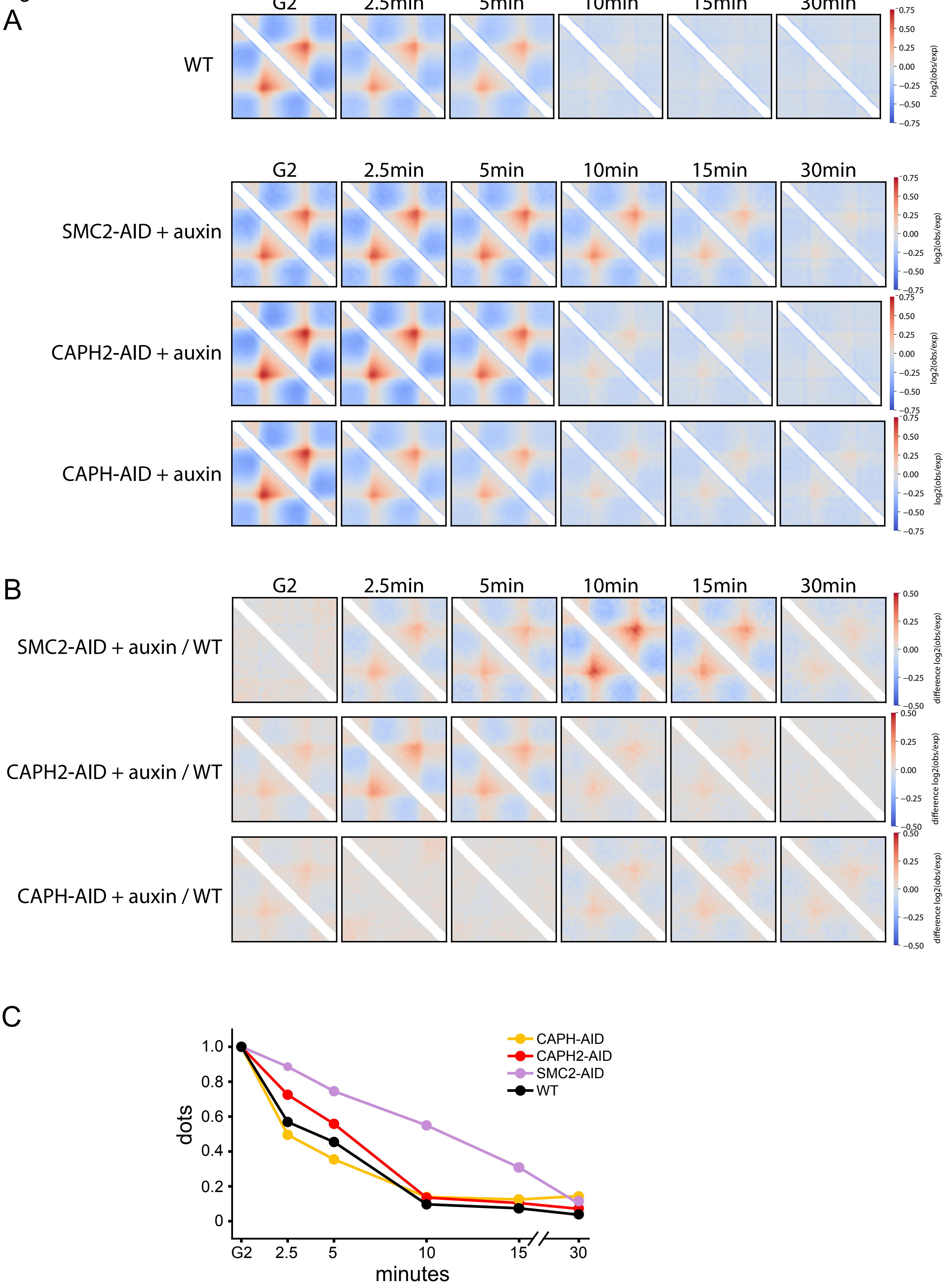

A
G2
2.5min
5min
10min
15min
30min
WT
SMC2-AID + auxin
CAPH2-AID + auxin
CAPH-AID + auxin
log2(obs/exp)
B
SMC2-AID + auxin / WT
CAPH2-AID + auxin / WT
CAPH-AID + auxin / WT
difference log2(obs/exp)
C
CAPH-AID
CAPH2-AID
SMC2-AID
WT
dots
minutes

Fig. S12. The dynamics of loop extrusion-related Hi-C features in SMC mutants. (**A**) Top: Average contact footprints (pileups) of G_2-detected 100-700kb TADs throughout prophase. The contact maps are normalized by the distance-dependent decay of contact frequency (observed/expected). Bottom: as above, in the mutants of both condensins (top row), condensin II (middle row), and condensin I (bottom row). (**B**) The ratio of observed/expected pileups in each of the three mutants vs the WT pileup at the matching timepoints. (C) The average intensity of G_2-detected dots throughout prophase in WT and three condensin mutants.

Figure S13

cell type	sorted	SMC2	CAP-H	CAP-H2	SMC3
SMC2-AID	no	undetected			
SMC3-AID	yes				undetected
SMC2-AID/SMC3-AID	yes	undetected			1%
SMC3-AID/CAPH-AID	yes	undetected			
SMC3-AID/CAPH2-AID	yes	undetected		undetected	
CAPH-AID	no		undetected		
CAPH2-AID	no			37%	

<u>Note</u>:
G2 blocked whole cell extracts were analysed.
GFP-ve G2 cells were sorted after cross-linking.
CAP-G2 protein was co-depleted (6%) in CAP-H2-AID cells.
The ratio (%) in mutant cells was calculated with the level in WT cells was set at 100%.

Distribution of GFP+ve or GFP-ve cells after sorting

WT: GFP^{-ve} control G2 (no sorting)

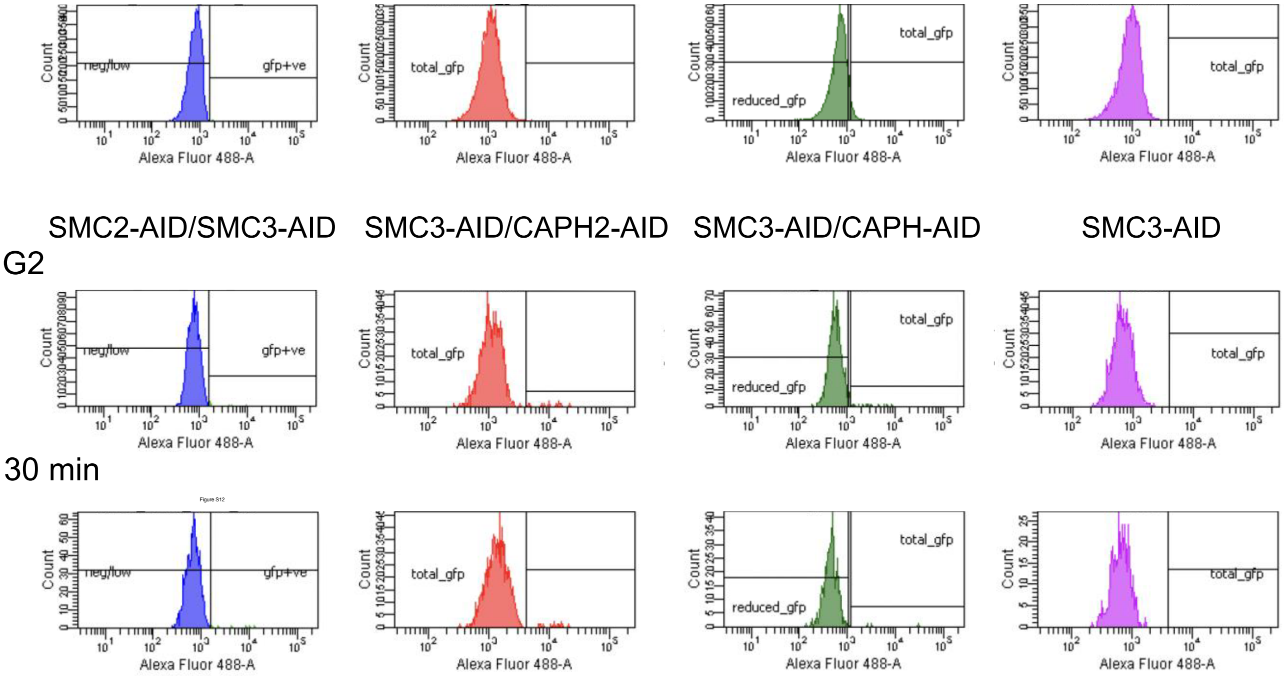

Fig. S13. Level of target protein depletion in cells used to prepare Hi-C samples. (**A**) Relative abundance of AID-GFP/clover tagged proteins in various mutant cells was compared to the abundance of the corresponding protein in wild type cells. Whole-cell mass spectrometry was performed on G_2 cells used to prepare Hi-C samples for the following genotypes: WT, SMC2-AID, CAP-H-AID, CAP-H2-AID, SMC2-AID/SMC3-AID, SMC3-AID, SMC3-AID/CAP-H-AID, and SMC3-AID/CAP-H2-AID. Target proteins were fused with an mAID tag and a clover (GFP derivative) tag. Depending on the efficiency of depletion as determined from flow cytometry (next panels) some cells were sorted for GFP after auxin addition. (**B**) Distribution of GFP-positive and GFP-negative cells at G_2 and at the 30 miniute time point after cell sorting. Control WT cells do not express GFP proteins, but the flow cytometer still detects auto fluorescence.

Table S2. The size of DAPI stained area in SMC2-AID cells. SMC2-AID cells were treated with auxin and nocodazole. DAPI stained areas (max projection) were measured using Fiji.

G_2	72	80.8
30 min	96	41.8

Table S3. Gyre size of chromosomes

**Experiment #**	Mbp/gyre		Mbp/gyre
**1**	8.99		7.27
**2**	9.84		6.75
**3**	10.6		
**4**	12.2		
**5**			8.2
**average**	10.41		7.41
**stdev**	1.36		0.73

Table S4. Measurements vs modeling of chromosome length and width

**LM**	length	7.7 ± 0.9	4.4 ± 0.5	13.6 ± 2.8
**EM**	length	6.3	5.3	12
**Simulations**	end to end	7	4.6	
**LM**	Tel — Tel	6.9 ± 0.8	3.7 ± 0.5	8.6 ± 1.4
**Simulations**	end to end			8
**EM**	width	0.8 ± 0.12	1.2 ± 0.17	0.6 ± 0.15
**Simulations**	width	0.6	1.1	0.6

Table S5: Second diagonal positions measured from P(s) derivative peaks (Mb)

**WT**		2.97	4.03	6.10		
**SMC3-AID**	2.86	3.82	5.19	8.43	11.44	13.95
**SMC3-CAPH-AID**			6.55	16.40		

Table S6: List of CDK1^{as} cell lines. All tags (mAID-Clover, clover, Halo) were knock-into the N-terminus or the C-terminus of the corresponding genes except CAP-H2-AID cells which utilized a cDNA to express CAP-H2-mAID-GFP. CAP-H2 gene was knocked out in this case. Previous AID tagged cell lines were made over conditional knock out cell lines (see (*1*)).

**wt**				
**SMC2-AID (new)**		mAID-Clover		OsTIR1
**SMC3-AID**		mAID-Clover		OsTIR1
**SMC2-AID/SMC3-AID**		mAID-Clover		OsTIR1
**CAP-H-AID (new)**		mAID-Clover		OsTIR1
**CAP-H2-AID (new)**			CAP-H2 cDNA-mAIDGFP	OsTIR1
**SMC3-AID/CAP-H2-AID**		mAID-Clover	CAP-H2 cDNA-mAIDGFP	OsTIR1
**SMC3-AID/CAP-H-AID**		mAID-Clover x2		OsTIR1
**SMC3-Halo**		Halo		
**CAP-H-Halo**		Halo		
**Halo-CAP-H2**	Halo			
**wt 3XGFP-NES Halo-LaminB1**	Halo		3XGFP-NES	
**SMC2-AID 3XCherry-NES**			3XCherry-NES	OsTIR1
**SMC3-AID 3XCherry-NES**			3XCherry-NES	OsTIR1
**SMC3-Clover/SMC2-Halo**		Clover, Halo		
**SMC3-AID/SMC2-Halo**		mAID-Clover, Halo		OsTIR1
**SMC3-AID/Halo-CAP-H2**	Halo	mAID-Clover		OsTIR1
**SMC3-AID/CAP-H2-AID/SMC2-Halo**		mAID-Clover, Halo	CAP-H2 cDNA-mAIDGFP	OsTIR1
**SMC3-AID/CAP-H-AID/Halo-CAPH2**	Halo	mAID-Clover x2		OsTIR1
**AID-wapl**	mAID-Clover			OsTIR1F74G
**AID-wapl /SMC2-Halo**	mAID-Clover	Halo		OsTIR1F74G

Table S7:List of oligos to construct guide RNA. CAPH2 knock out guide RNA is cloned into CRIPSR-Cas9 vector from Thermofisher Scientific. All others are cloned into pX330 (addgene).

**SMC2**	knockin	CACCGAGTGAAGCCAGCAACAACA
**SMC2**	knockin	AAACTGTTGTTGCTGGCTTCACTC
**SMC3**	knockin	CACCGCAATTTCGCTTAACCATGCG
**SMC3**	knockin	AAACCGCATGGTTAAGCGAAATTGC
**CAPH**	knockin	CACCGTCTGATGTCATTGTGAAACA
**CAPH**	knockin	AAACTGTTTCACAATGACATCAGAC
**CAPH2**	knockin	CACCGCGTGACTCCACATCCTCCA
**CAPH2**	knockin	AAACTGGAGGATGTGGAGTCACGC
**CAPH2**	knockout	TTCTTGGTGAGGTCACGGATGTTTT
**CAPH2**	knockout	ATCCGTGACCTCACCAAGAACGGTG
**lamin B1**	knockin	CACCGTCCCCTACCATCACGTCACG
**lamin B1**	knockin	AAACCGTGACGTGATGGTAGGGGAC
**wapl**	knockin	CACCGCCAAAATGACATCCCGATT
**wapl**	knockin	AAACAATCGGGATGTCATTTTGGC

Table S1. Cell line characteristics for all libraries used in this study

The name of the "Figure alias" (i.e. WT-2.5min-S4-R2-spna) is a combination of the following relevant information: cell type - timepoint - set - replicate - treatment. Sets of cells were derived from the same biological replicates and generally processed together. Set numbering continued from previously published sets (*6*). Treatments are sorting (S), palbociclib (P), nocodazole (N) and auxin (A), where capital letters indicate addition and small letters indicate omission thereof. Addition of MG132 was occasionally indicated with a capital M.

Movies 1-4: 3D reconstructions of SBF-SEM datasets of all DT40 chromosomes.

All datasets were modeled in Amira to differentiate chromosomes from the cytoplasm and then segmented to assign different colors to each individual chromosome. Movies are as follows: (**1**) Chromosome complement from wild-type DT40 cell; (**2**) Chromosome complement from SMC3-AID cell plus auxin; (**3**) Chromosome complement from SMC3-AID/CAP-H-AID cell plus auxin; (**4**) Chromosome complement from SMC3-AID/CAP-H2-AID cell plus auxin.

Movies 5-8: 3D reconstructions of SBF-SEM datasets for chromosome 1.
All datasets were modeled in Amira and segmented to assign different colors to each chromosome. Chromosome 1 was arbitrarily defined as here defined as the longest chromosome in each cell. Movies are as follows: (**5**) Chromosome 1 from wild-type DT40 cell; (**6**) Chromosome 1 from SMC3-AID cell plus auxin; (**7**) Chromosome 1 from SMC3-AID/CAP-H-AID cell plus auxin; (**8**) Chromosome 1 from SMC3-AID/CAP-H2-AID cell plus auxin.
